# Exploratory Mixed-Data Phenomapping and Risk-Enrichment Frameworks for Long-Term Mortality After Acute Coronary Syndrome: A Single-Center Cohort Study

**DOI:** 10.3390/medicina62081586

**Published:** 2026-08-18

**Authors:** Aykan Çelik, Uğur Kocabaş, Tuncay Kırış, Ali Efe Afşin, Harun Erdem, Elif Naz Uçak, Semih Babacan, Mustafa Karaca

**Affiliations:** Department of Cardiology, Faculty of Medicine, Ataturk Training and Research Hospital, Izmir Katip Celebi University, 35620 Izmir, Türkiye

**Keywords:** acute coronary syndrome, all-cause mortality, Gower distance, partitioning around medoids, phenomapping, risk enrichment

## Abstract

*Background and Objectives*: Long-term prognosis after acute coronary syndrome (ACS) may reflect multidomain baseline characteristics. We evaluated outcome-independent mixed-data phenotypes and post hoc additive frameworks for all-cause mortality. *Materials and Methods*: Of 3801 consecutive patients hospitalized with ACS during 2017–2024, 3561 formed the survival cohort after 240 patients lacking mortality status or valid follow-up were excluded. Gower–PAM phenomapping included 3491 patients after 70 patients with incomplete clustering variables were excluded. Cluster number was selected among k = 2–4 using prespecified outcome-independent silhouette, minimum-size, and bootstrap-stability criteria. AAR-3, CARE-4, and ACEF were compared in a common complete-case cohort of 3492 patients using an age-inclusive clinical reference model. *Results*: During a median follow-up of 3.35 years, 407 deaths occurred in the survival cohort. Three Gower–PAM phenotypes were selected (average silhouette width, 0.417), with crude mortality proportions of 12.1%, 4.2%, and 19.2% in phenotypes A, B, and C, respectively (global log-rank *p* < 0.001). The phenotype factor improved the primary age-inclusive model (joint *p* < 0.001), although only phenotype B differed from phenotype A after adjustment. The joint phenotype effect was attenuated after expanded clinical adjustment (*p* = 0.673), and agreement with k-means sensitivity analyses was low. In the common cohort, the first-year adjusted HRs were 1.78 per 1-point increase in AAR-3 (95% CI, 1.46–2.19), 2.08 per 1-point increase in CARE-4 (95% CI, 1.76–2.46), and 2.66 per 1-unit increase in ACEF (95% CI, 2.14–3.30). The corresponding >365-day landmark HRs were 1.56 (95% CI, 1.32–1.85), 1.66 (95% CI, 1.44–1.91), and 2.39 (95% CI, 1.95–2.92), respectively. Full-follow-up C-indices were 0.793 (95% CI, 0.772–0.814) with AAR-3, 0.815 (95% CI, 0.795–0.834) with CARE-4, and 0.812 (95% CI, 0.792–0.833) with ACEF, compared with 0.770 (95% CI, 0.747–0.792) for the age-inclusive reference model. None materially improved the model that already contained age, hemoglobin, eGFR, and LVEF continuously. *Conclusions*: Outcome-independent phenomapping identified exploratory, method-dependent patterns rather than ordered biological risk classes. The post hoc additive frameworks were internally associated with mortality but should not be regarded as validated prediction or treatment-directing tools. Prospective multicenter external validation is required before clinical application.

## 1. Introduction

Despite major advances in the management and secondary prevention of acute coronary syndrome (ACS), substantial heterogeneity in long-term outcomes remains after hospital discharge [1]. Established risk assessment incorporates demographic characteristics, comorbidities, hemodynamic status, anatomical disease burden, and procedural factors. Routinely measured characteristics such as age, anemia, renal dysfunction, reduced left ventricular ejection fraction, and inflammatory indices have also been individually associated with adverse cardiovascular outcomes [2,3,4,5,6,7,8,9,10,11,12]. However, the extent to which these characteristics co-occur in distinguishable baseline patterns after ACS remains incompletely characterized.

Data-driven phenotyping can describe heterogeneity within cardiovascular populations and has been applied particularly in heart failure and multimorbidity settings [13,14,15,16]. Its application to real-world ACS cohorts remains limited. A further methodological challenge is that routine clinical datasets contain both continuous measurements and categorical characteristics. Clustering results may therefore depend on the selected dissimilarity measure, variable coding, and clustering algorithm. Mixed-data approaches such as Gower dissimilarity with partitioning around medoids can accommodate these variable types without treating binary characteristics as continuous Euclidean measurements. Nevertheless, any resulting clusters remain exploratory statistical partitions; within-method stability does not establish biological mechanisms, reproducibility across methods, or transportability to other populations.

Simple additive summaries may offer an interpretable way to describe the accumulation of abnormalities across routinely assessed domains. In the present study, AAR-3 and CARE-4 were constructed post hoc after examination of the dominant domains represented in the phenomapping analysis. They were not prespecified clinical prediction scores and were evaluated in the same institutional cohort in which they were formulated. Their equal weighting and thresholds were selected for clinical interpretability rather than through statistical optimization. Consequently, their evaluation requires age-inclusive reference models, direct comparison within the same complete-case cohort, and assessment against models containing the underlying variables continuously.

The objectives of this study were therefore to: (1) identify outcome-independent patterns of baseline multidomain characteristics using Gower-PAM mixed-data clustering; (2) describe the associations between the resulting phenotypes and long-term all-cause mortality; and (3) evaluate the exploratory AAR-3 and CARE-4 frameworks against an age-inclusive clinical reference model and the established ACEF comparator in a common complete-case cohort. We additionally examined whether the proposed summaries contributed information beyond their underlying continuous domains and assessed the internal consistency of the findings using multiple imputation, time-specific survival analyses, temporal-period analyses, and cross-validated decision-curve analysis.

## 2. Materials and Methods

### 2.1. Study Design and Population

This retrospective, single-center cohort study included consecutive patients hospitalized with acute coronary syndrome (ACS) at a tertiary cardiovascular center between January 2017 and December 2024. The initial cohort comprised 3801 patients. For time-to-event analyses, 240 patients were excluded because mortality status was unavailable or follow-up duration was missing or non-positive. The mutually exclusive exclusion categories were missing mortality status only (*n* = 102), missing follow-up duration only (*n* = 37), non-positive follow-up duration only (*n* = 60), missing mortality status with non-positive follow-up duration (*n* = 24), and missing mortality status with missing follow-up duration (*n* = 17). The final survival cohort therefore comprised 3561 patients with ascertainable mortality status and valid positive follow-up.

The primary mixed-data phenomapping analysis required complete data for age, hemoglobin, estimated glomerular filtration rate (eGFR), left ventricular ejection fraction (LVEF), log-transformed neutrophil-to-lymphocyte ratio (NLR) and systemic immune-inflammation index (SII), diabetes mellitus, hypertension, atrial fibrillation, and current smoking. Seventy patients in the survival cohort lacked at least one of these variables; therefore, 3491 patients were included in the phenomapping analysis. Primary head-to-head comparisons of AAR-3, CARE-4, ACEF, and the continuous biological-domain model were conducted in a common complete-case cohort of 3492 patients with complete data for age, sex, ACS subtype, diabetes mellitus, hypertension, atrial fibrillation, current smoking, prior coronary artery disease, creatinine, hemoglobin, eGFR, and LVEF.

Variable-level missingness was summarized for the initial cohort. To assess the potential for selection bias, baseline characteristics were compared between patients included in and excluded from the survival cohort using standardized mean differences, with conventional hypothesis tests reported descriptively.

Sex was recorded as a biological variable from electronic health records and was included in descriptive and multivariable analyses. Gender identity was not available in the retrospective dataset.

The study was approved by the institutional review board of Izmir Katip Celebi University, Ataturk Training and Research Hospital Ethics Committee (approval number: 0507; date: 11 June 2026) and was conducted in accordance with the Declaration of Helsinki. Due to the retrospective observational design, informed consent was waived by the institutional ethics committee.

### 2.2. Clinical Data Collection

Baseline demographic, clinical, laboratory, and echocardiographic data were obtained from institutional electronic medical records. For laboratory variables, baseline values were defined as the first available measurements obtained at presentation for the index ACS hospitalization. LVEF was obtained from transthoracic echocardiography performed within the first 24–48 h of the index hospitalization. Accordingly, all variables used in the primary phenomapping and vulnerability-framework analyses represented admission or early index-hospitalization assessments rather than longitudinal or post-discharge measurements.

ACS presentations were classified as ST-segment elevation myocardial infarction (STEMI), non-ST-segment elevation myocardial infarction (NSTEMI), or unstable angina according to contemporary definitions and guideline-based clinical classification [1,17]. The evaluated biological and clinical variables included age at the index hospitalization, hemoglobin, creatinine, estimated glomerular filtration rate (eGFR), LVEF, neutrophil-to-lymphocyte ratio (NLR), systemic immune-inflammation index (SII), diabetes mellitus, hypertension, atrial fibrillation, current smoking, and prior coronary artery disease. The eGFR was calculated using the CKD-EPI equation [18]. NLR was calculated by dividing the absolute neutrophil count by the absolute lymphocyte count, whereas SII was calculated as platelet count × neutrophil count/lymphocyte count [6,8]. Because NLR and SII had right-skewed distributions, they were logarithmically transformed before clustering analyses.

Detailed discharge pharmacotherapy, longitudinal medication adherence, completeness of revascularization, procedural complexity, cardiac rehabilitation participation, and serial biomarker trajectories were not systematically retrievable throughout the study period. Therefore, these variables were not included in the primary analyses, and their potential influence was considered when interpreting the results.

### 2.3. Outcome Definition

The primary endpoint was all-cause mortality during follow-up. Vital status and date of death were ascertained using hospital electronic medical records and the national health information system. For patients with a documented death, follow-up ended on the recorded date of death. Patients without a documented death in either source were confirmed to be alive at their last available follow-up date, and their observations were right-censored on that date. No assumption was made regarding their vital status beyond the censoring date.

### 2.4. Unsupervised Phenomapping Analysis

To identify multidomain phenotypic patterns in data containing both continuous and categorical variables, the primary unsupervised phenomapping analysis was performed using Gower dissimilarity and partitioning around medoids (PAM). The analysis included six continuous variables—age, hemoglobin, eGFR, LVEF, log-transformed NLR, and log-transformed SII—and four binary categorical variables—diabetes mellitus, hypertension, atrial fibrillation, and current smoking. NLR and SII were transformed using log(1 + x). The four binary variables—diabetes mellitus, hypertension, atrial fibrillation, and current smoking—were encoded as two-level nominal factors in the Gower calculation; no asymmetric-binary specification was used. Accordingly, ordinary nominal matching was applied, such that both joint absence and joint presence contributed to similarity. No custom variable weights were specified, and all ten clustering variables therefore received equal unit weight. Equal weighting was chosen to preserve a transparent, outcome-independent unsupervised specification and to avoid introducing subjective or outcome-informed variable weighting. Because Gower dissimilarity places variable-specific contributions on comparable scales, this approach also prevents differences in raw measurement units from determining the distance calculation. Mortality status and follow-up duration were not included in the clustering input.

Candidate PAM solutions with k = 2, 3, and 4 clusters were evaluated using an outcome-independent selection procedure. For each candidate solution, internal separation was quantified using the average silhouette width, and cluster stability was assessed using 100 bootstrap resamples and cluster-specific Jaccard coefficients. Candidate solutions were considered eligible if every cluster contained at least 10% of the phenomapping cohort and every cluster had a bootstrap Jaccard coefficient of at least 0.75. Among eligible solutions, the number of clusters was selected by maximizing the average silhouette width, with the smaller value of k preferred only in the event of an exact tie. Mortality and follow-up information were not used to select the number of clusters, construct the clusters, order them, or assign phenotype labels.

After the cluster solution had been finalized, neutral phenotype labels were assigned in descending order of cluster size as PAM phenotypes A, B, and C. Clinical characteristics and mortality outcomes were examined only after the clustering procedure and phenotype labeling had been completed. As method-sensitivity analyses, clustering was repeated using standardized mixed-variable k-means and standardized continuous-only k-means with the number of clusters fixed to that selected by the primary PAM analysis and 50 random initializations. Internal separation was compared using average silhouette widths, and patient-level agreement between clustering methods was quantified using the adjusted Rand index.

### 2.5. AAR-3 and CARE-4 Vulnerability Burden Frameworks

AAR-3 and CARE-4 were constructed as exploratory, post hoc vulnerability-burden frameworks after inspection of the multidomain patterns identified in the phenomapping analysis. They were intended to translate the recurrent age-related, hematologic, renal, and cardiac functional domains into transparent summary measures using routinely available clinical variables. The frameworks were not prespecified before phenomapping, algorithmically derived from the cluster assignments, optimized against mortality outcomes, or developed as treatment-directing prediction tools. Because they were constructed and evaluated within the same cohort, their analyses should be considered exploratory and hypothesis-generating.

AAR-3 (Age–Anemia–Renal dysfunction) was calculated as the sum of three binary components: age ≥ 75 years, anemia, and eGFR < 60 mL/min/1.73 m^2^. The age threshold of ≥75 years was selected as a clinically interpretable indicator of advanced age rather than derived through optimization against cluster separation or mortality outcomes. Anemia was defined according to sex-specific World Health Organization thresholds as hemoglobin <13.0 g/dL in men and <12.0 g/dL in women [19]. Renal dysfunction was defined using the clinically established eGFR threshold of <60 mL/min/1.73 m^2^ [20]. Each component contributed 1 point, producing an AAR-3 burden ranging from 0 to 3.

CARE-4 (Cardiac dysfunction–Age–Renal dysfunction–Anemia) extended AAR-3 by adding LVEF < 40%, a guideline-recognized threshold for reduced left ventricular systolic function [21]. Each of the four components contributed 1 point, producing a CARE-4 burden ranging from 0 to 4. Equal weighting was chosen to preserve transparency, parsimony, and ease of interpretation and was not intended to imply that the four components had identical biological or prognostic effects. No regression coefficients, outcome-derived weights, or data-dependent cutoffs were used to calculate either framework.

AAR-3 could be calculated in 3557 patients with available age, hemoglobin, and eGFR data, whereas CARE-4 could be calculated in 3505 patients with additional LVEF availability. These framework-specific cohorts were used for descriptive analyses and category-based Kaplan–Meier analyses. Primary head-to-head comparisons of AAR-3, CARE-4, and ACEF, including analyses of their incremental prognostic value, were conducted in the common complete-case cohort of 3492 patients defined above.

### 2.6. Survival, Prognostic Performance, and Internal Robustness Analyses

Time-to-event distributions were estimated using the Kaplan–Meier method and compared using the log-rank test. Kaplan–Meier analyses were performed for AAR-3 and CARE-4 burden categories and for the PAM-derived phenotypes. Associations with all-cause mortality were evaluated using Cox proportional hazards regression with Efron handling of tied event times. PAM phenotype associations were examined in unadjusted models and in age-inclusive clinical models incorporating age, sex, ACS subtype, and prior coronary artery disease. An expanded sensitivity model additionally included diabetes mellitus, hypertension, atrial fibrillation, and current smoking.

Primary head-to-head comparisons of AAR-3, CARE-4, and ACEF were performed in the common complete-case cohort of 3492 patients with 394 deaths. Model B was designated as the primary clinical reference model and included chronological age, sex, ACS subtype, diabetes mellitus, hypertension, atrial fibrillation, current smoking, and prior coronary artery disease. Each vulnerability framework was added separately to Model B to quantify its association and incremental prognostic contribution beyond chronological age and the conventional clinical covariates. Model A, which contained the same clinical covariates but omitted chronological age, was retained only as a historical sensitivity analysis to facilitate comparison with the originally submitted models. Model C extended Model B by incorporating continuous hemoglobin, eGFR, and LVEF and was used to determine whether the simplified vulnerability frameworks contributed information beyond their underlying continuous biological components. ACEF was calculated as age in years divided by LVEF in percentage points, with 1 additional point assigned when serum creatinine was >2.0 mg/dL [22].

Nested models were compared using likelihood-ratio tests. Model performance was summarized using Harrell’s C-index, Akaike information criterion, and Bayesian information criterion. For the primary common-cohort Model B comparisons, uncertainty in discrimination was quantified using 95% confidence intervals. Confidence intervals for individual C-indices were derived from their model-based standard errors, whereas 95% confidence intervals for changes in C-index were estimated using 2000 paired patient-level nonparametric bootstrap resamples, with all compared models refitted within the same resampled cohort. The paired bootstrap was also used to quantify the difference in C-index between the CARE-4- and ACEF-extended Model B models. Proportional hazards assumptions were assessed at both the individual-variable and global-model levels using tests based on scaled Schoenfeld residuals. Because evidence of non-proportionality was observed in some overall models, associations were additionally estimated separately for the first year and the period beyond 1 year. For the 0–365-day analysis, follow-up was censored at 365 days. For the late-period analysis, a 365-day landmark approach was used among patients who were alive and remained under observation beyond 1 year, with analysis time beginning at the landmark. These time-window-specific estimates were emphasized when interpreting variables for which the proportional hazards assumption was not fully satisfied, whereas overall hazard ratios were interpreted as average associations across follow-up.

As a sensitivity analysis for missing covariate data, multiple imputation by chained equations was performed within the 3561-patient survival cohort under a missing-at-random assumption. Patients excluded because of unavailable mortality status or invalid follow-up were not reintroduced through imputation. Twenty imputed datasets were generated using 20 iterations. Predictive mean matching was used for age, creatinine, eGFR, hemoglobin, and LVEF; polytomous logistic regression was used for ACS subtype; and binary logistic regression was used for diabetes mellitus, hypertension, atrial fibrillation, current smoking, and prior coronary artery disease. The event indicator and Nelson-Aalen cumulative hazard were included as auxiliary predictors, whereas raw follow-up duration was not used as an imputation predictor. Survival outcomes were not imputed. AAR-3, CARE-4, and ACEF were recalculated after imputation within each completed dataset. Cox regression coefficients and confidence intervals were combined using Rubin’s rules, and pooled incremental model comparisons were performed using D1 tests. Convergence was assessed using trace plots, autocorrelation diagnostics, and logged imputation events. Detailed convergence diagnostics are presented in Supplementary Figure S1 and Table S8.

Adjusted restricted cubic spline analyses were performed in the common complete-case cohort to assess the continuous functional forms of age, hemoglobin, eGFR, and LVEF. Four knots were placed at the 5th, 35th, 65th, and 95th percentiles of each exposure. The exposure-specific median was used as the hazard-ratio reference. The age model was adjusted for sex, ACS subtype, diabetes mellitus, hypertension, atrial fibrillation, current smoking, and prior coronary artery disease; the hemoglobin, eGFR, and LVEF models were additionally adjusted for chronological age. Overall and nonlinear associations were evaluated separately. Curves were displayed from the 1st to the 99th percentile, with 95% confidence intervals and rug plots showing the observed exposure distributions.

As secondary internal-validation analyses, the framework-specific AAR-3 and CARE-4 models underwent bootstrap optimism correction using 500 bootstrap resamples and 1-year calibration using 300 bootstrap resamples. Discrimination was also examined using 10-fold cross-validation repeated 200 times. These supportive framework-specific models were adjusted for sex, ACS subtype, diabetes mellitus, hypertension, atrial fibrillation, current smoking, and history of coronary artery disease. Chronological age was not entered separately because age is a component of both AAR-3 and CARE-4. These validation estimates were considered supportive and were not used as the primary head-to-head comparison between AAR-3, CARE-4, and ACEF. Bootstrap stability of the primary Gower–PAM cluster solution was assessed as described in Section 2.4.

A censoring-aware survival decision-curve analysis was performed for 1-year all-cause mortality in the common complete-case cohort. Absolute 1-year risk predictions were generated out of fold using stratified 10-fold cross-validation repeated 20 times, such that each patient received predictions only from models fitted without that patient. The resulting 20 out-of-fold predictions for each patient were averaged. Unlike the originally applied binary-outcome approach, patients censored before 365 days were retained, and censoring was handled through the survival endpoint. Net benefit was evaluated across threshold probabilities from 1% to 20% in increments of 0.5% for Model B, Model B plus AAR-3, Model B plus CARE-4, Model B plus ACEF, and Model B incorporating continuous hemoglobin, eGFR, and LVEF. Decision-curve findings were interpreted as exploratory decision-analytic results and not as evidence supporting clinical implementation or treatment decisions.

As an additional internal temporal robustness analysis, the common complete-case cohort was divided into two non-overlapping calendar-period subcohorts: an earlier period comprising admissions from January 2017 to December 2019 and a later period comprising admissions from January 2020 to December 2024. The 2020 cut-point was selected pragmatically to divide the study population into two chronologically distinct periods of broadly comparable size while retaining sufficient observations in each period for stable model estimation. The cut-point was not selected on the basis of mortality rates, effect estimates, C-indices, p-values, or other model-performance results. This analysis was intended as an internal assessment of temporal robustness rather than as a formal derivation–validation split. Within each period, separate Cox models evaluated AAR-3, CARE-4, and ACEF using an age-inclusive clinical reference model containing age, sex, ACS subtype, diabetes mellitus, hypertension, atrial fibrillation, and history of coronary artery disease. Current smoking was omitted from both period-specific models because the 2017–2019 common cohort included only 25 current smokers and none experienced the outcome, resulting in complete separation; the same covariate set was used in both periods to preserve comparability. Results were summarized using hazard ratios with 95% confidence intervals, Harrell’s C-index, and likelihood-ratio tests. The proportional hazards assumption was evaluated using variable-level and global Schoenfeld residual tests.

### 2.7. Statistical Analysis

Continuous variables were presented as mean ± standard deviation or median with interquartile range according to distribution characteristics. Categorical variables were expressed as counts and percentages. For two-group comparisons, Student’s *t*-test or Mann–Whitney U test was used for continuous variables, and Pearson’s chi-square test was used for categorical variables. For comparisons across more than two vulnerability burden categories, one-way analysis of variance or the Kruskal–Wallis test was used for continuous variables, and Pearson’s chi-square test was used for categorical variables. A two-sided *p*-value < 0.05 was considered statistically significant. Because several secondary, sensitivity, and exploratory analyses were performed, these analyses were interpreted as supportive rather than confirmatory, and no formal multiplicity correction was applied.

All analyses were performed using the R programming language (R Foundation for Statistical Computing, Vienna, Austria, version 4.6.0) within the RStudio environment version 2026.04.0 (Posit PBC, Boston, MA, USA).

### 2.8. Patient and Public Involvement

Patients or members of the public were not involved in the design, conduct, reporting, interpretation, or dissemination plans of this retrospective study.

### 2.9. Reporting Guidelines

This study was conducted and reported in accordance with the STROBE statement (File S1) [23]. Elements of TRIPOD+AI relevant to transparent reporting of data-driven phenotyping, vulnerability framework operationalization, and prognostic performance assessment were considered where applicable; however, this study was not designed as a formal de novo clinical prediction model development study or external validation study (File S2) [24].

### 2.10. Use of Generative Artificial Intelligence in Manuscript Preparation

Generative artificial intelligence was used only for language refinement and structural editing during manuscript preparation. It was not used for study design, data collection, data generation, statistical analysis, or scientific interpretation. All generated outputs were critically reviewed and revised by the authors.

## 3. Results

### 3.1. Study Population

**Figure 1:**
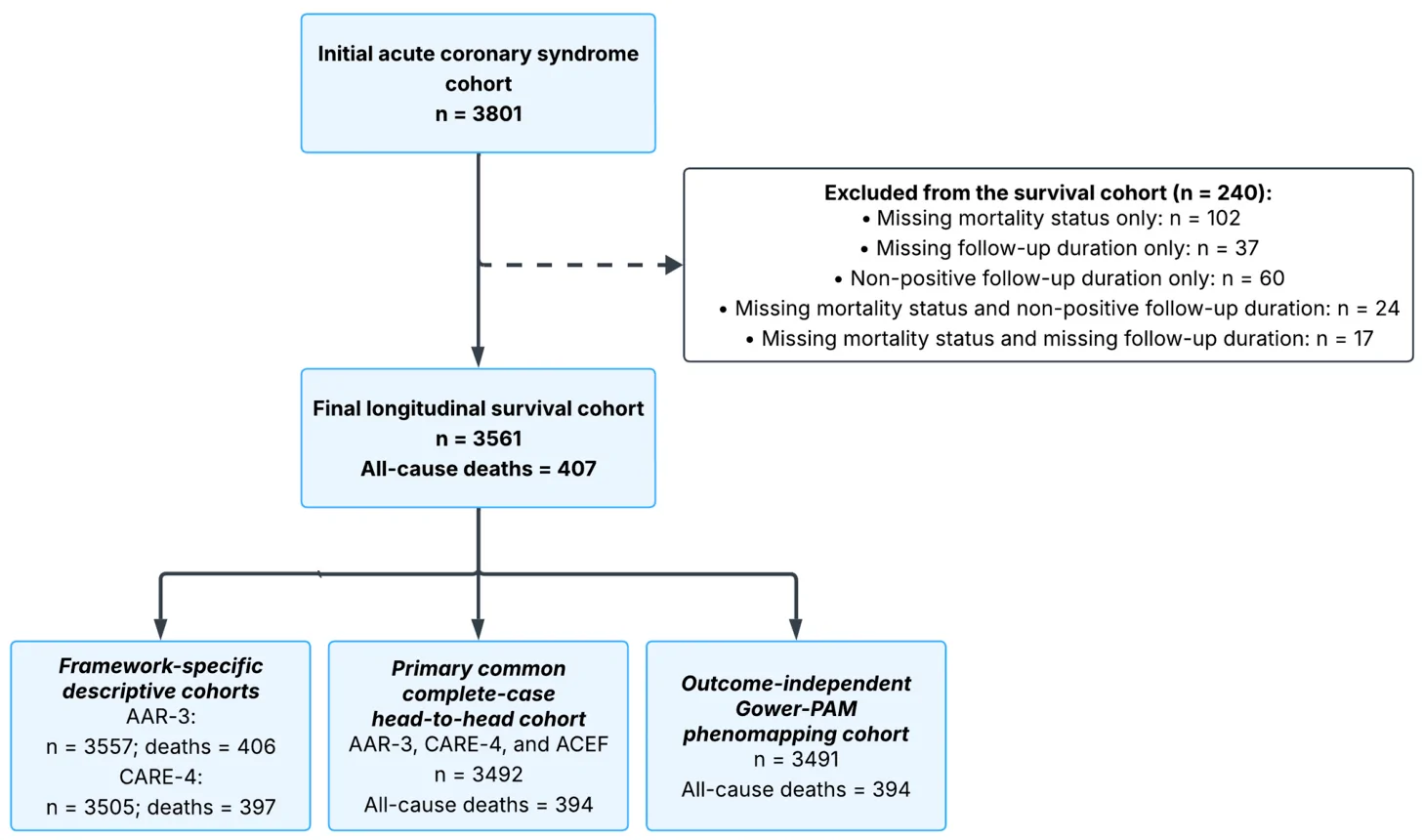
Study flow diagram. Of 3801 consecutive patients hospitalized with acute coronary syndrome, 240 were excluded according to five mutually exclusive categories, leaving 3561 patients in the survival cohort. The diagram also shows the framework-specific descriptive cohorts, the outcome-independent Gower-PAM phenomapping cohort, and the common complete-case cohort used for the primary head-to-head comparison.

The initial cohort consisted of 3801 consecutive patients hospitalized with acute coronary syndrome. After exclusion of 240 patients with unavailable mortality status or missing or non-positive follow-up duration, the final survival cohort comprised 3561 patients. The five mutually exclusive exclusion categories are presented in Figure 1. During a median follow-up of 3.35 years, 407 all-cause deaths occurred.

Variable-level missingness in the initial and survival cohorts is reported in Supplementary Table S1. Compared with patients included in the survival cohort, excluded patients were older (61.3 versus 56.8 years), more frequently female (30.0% versus 23.8%), and had higher prevalences of diabetes mellitus (27.5% versus 17.6%) and hypertension (47.1% versus 26.3%), together with a lower median eGFR (71.0 versus 95.7 mL/min/1.73 m^2^). Complete comparisons, including standardized mean differences, are provided in Supplementary Table S2. These differences indicate that the exclusions were unlikely to have occurred completely at random and should be considered when interpreting potential selection bias.

The outcome-independent Gower-PAM phenomapping cohort included 3491 patients with 394 deaths. Framework-specific descriptive and category-based analyses included 3557 patients with 406 deaths for AAR-3 and 3505 patients with 397 deaths for CARE-4. The primary head-to-head comparison of AAR-3, CARE-4, and ACEF was performed in the common complete-case cohort of 3492 patients with 394 deaths (Figure 1).

### 3.2. Baseline Characteristics According to Vulnerability Burden

Baseline clinical and biological characteristics across CARE-4 burden categories are presented in Table 1. Because age, anemia/hemoglobin, eGFR, and LVEF directly define CARE-4, differences in these variables across CARE-4 categories are structurally expected and should be interpreted descriptively rather than as independent inferential findings. Higher CARE-4 categories were also accompanied by differences in several noncomponent characteristics, including comorbidity burden and inflammatory indices. Observed all-cause mortality increased from 3.4% in patients with CARE-4 = 0 to 51.4% in patients with CARE-4 = 4. Because the CARE-4 = 4 category contained only 35 patients, its mortality estimate should be interpreted cautiously.

A similar descriptive gradient was observed across AAR-3 categories. Because age, anemia/hemoglobin, and eGFR directly define AAR-3, differences in these variables are likewise structurally expected. Observed all-cause mortality increased from 4.8% in patients with AAR-3 = 0 to 42.4% in patients with AAR-3 = 3.

### 3.3. Outcome-Independent Gower-PAM Phenotypes

Among the candidate solutions, the average silhouette width was 0.402 for k = 2, 0.417 for k = 3, and 0.412 for k = 4. The three-cluster solution was selected because it had the highest average silhouette width among the solutions satisfying the prespecified cluster-size and stability criteria. All three clusters contained more than 10% of the phenomapping cohort, with bootstrap Jaccard similarities of 0.964, 1.000, and 0.893, and no cluster dissolution across 100 resamples. Although all four clusters in the k = 4 solution also exceeded 10% of the cohort, one cluster had a bootstrap Jaccard similarity of 0.703 and failed the prespecified stability criterion. Mortality and follow-up information were not used for cluster-number selection or phenotype labeling.

Neutral phenotype labels were assigned in descending order of cluster size. Phenotype A included 1969 patients (56.4%), phenotype B included 919 patients (26.3%), and phenotype C included 603 patients (17.3%). Phenotype B comprised younger patients with higher hemoglobin levels, lower inflammatory indices, and current smoking in all members, whereas phenotype C was characterized by older age, lower estimated glomerular filtration rate, higher inflammatory indices, and high prevalences of diabetes and hypertension. Detailed phenotype characteristics are provided in Supplementary Table S5, with clustering diagnostics and sensitivity analyses presented in Supplementary Tables S4 and S6.

After completion of the outcome-independent clustering procedure, all-cause mortality was 12.1% in phenotype A, 4.2% in phenotype B, and 19.2% in phenotype C (global log-rank *p* < 0.001). The primary Gower-PAM solution showed greater internal separation than the standardized mixed-variable and continuous-only k-means sensitivity analyses, with average silhouette widths of 0.417, 0.192, and 0.219, respectively. However, patient-level agreement was low, with adjusted Rand indices of 0.049 and 0.026. The phenotypes should therefore be interpreted as exploratory, method-dependent patterns rather than ordered or externally reproducible biological risk classes.

### 3.4. Survival Analyses

**Figure 2:**
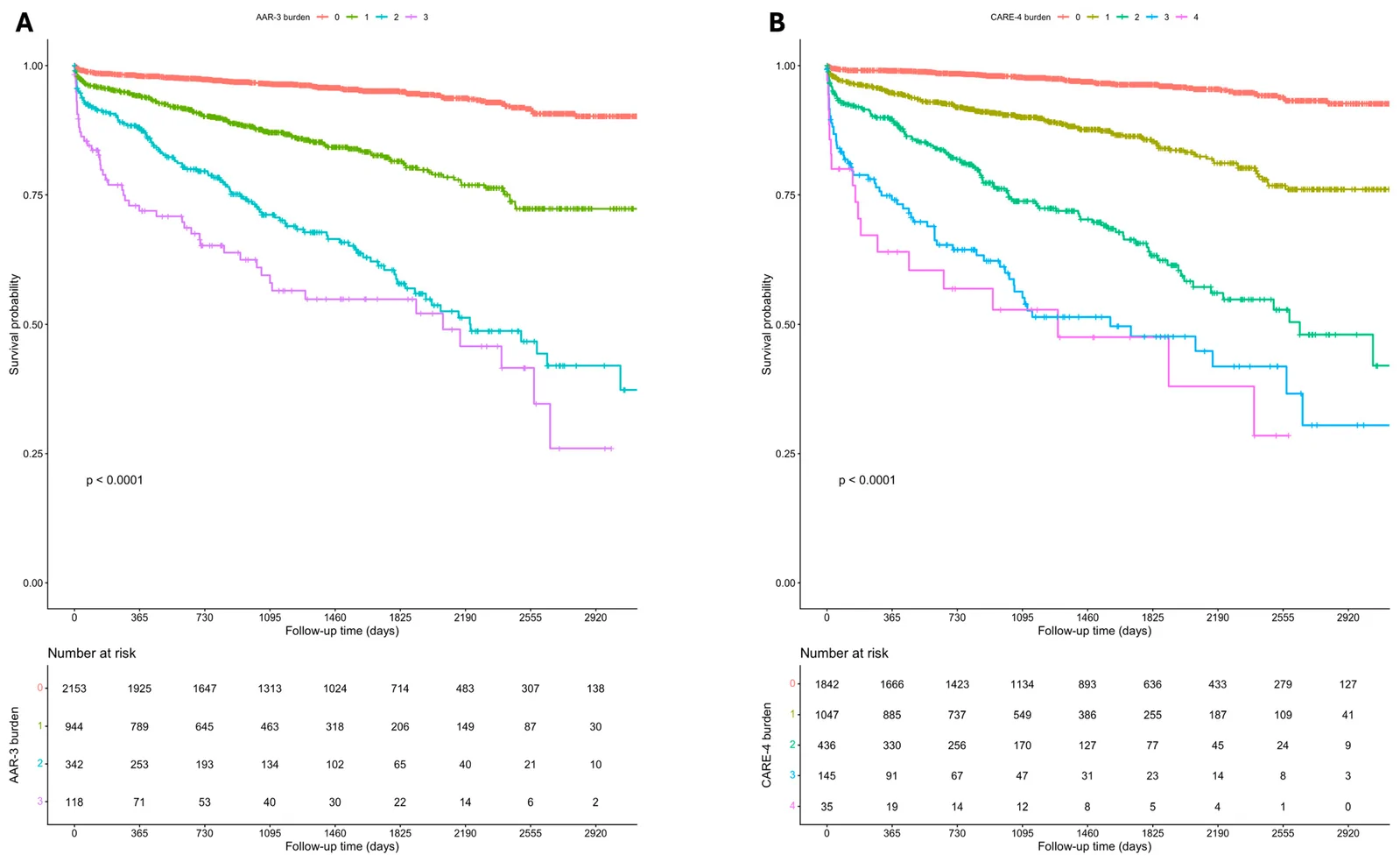
Kaplan–Meier curves according to vulnerability-burden frameworks. (**A**) Kaplan–Meier curves for long-term all-cause mortality according to AAR-3 burden categories. (**B**) Kaplan–Meier curves for long-term all-cause mortality according to CARE-4 burden categories. Increasing vulnerability burden was associated with progressively lower survival probabilities in both frameworks (log-rank *p* < 0.001 for both). The highest CARE-4 burden category included a small number of patients and should therefore be interpreted cautiously.

**Figure 3:**
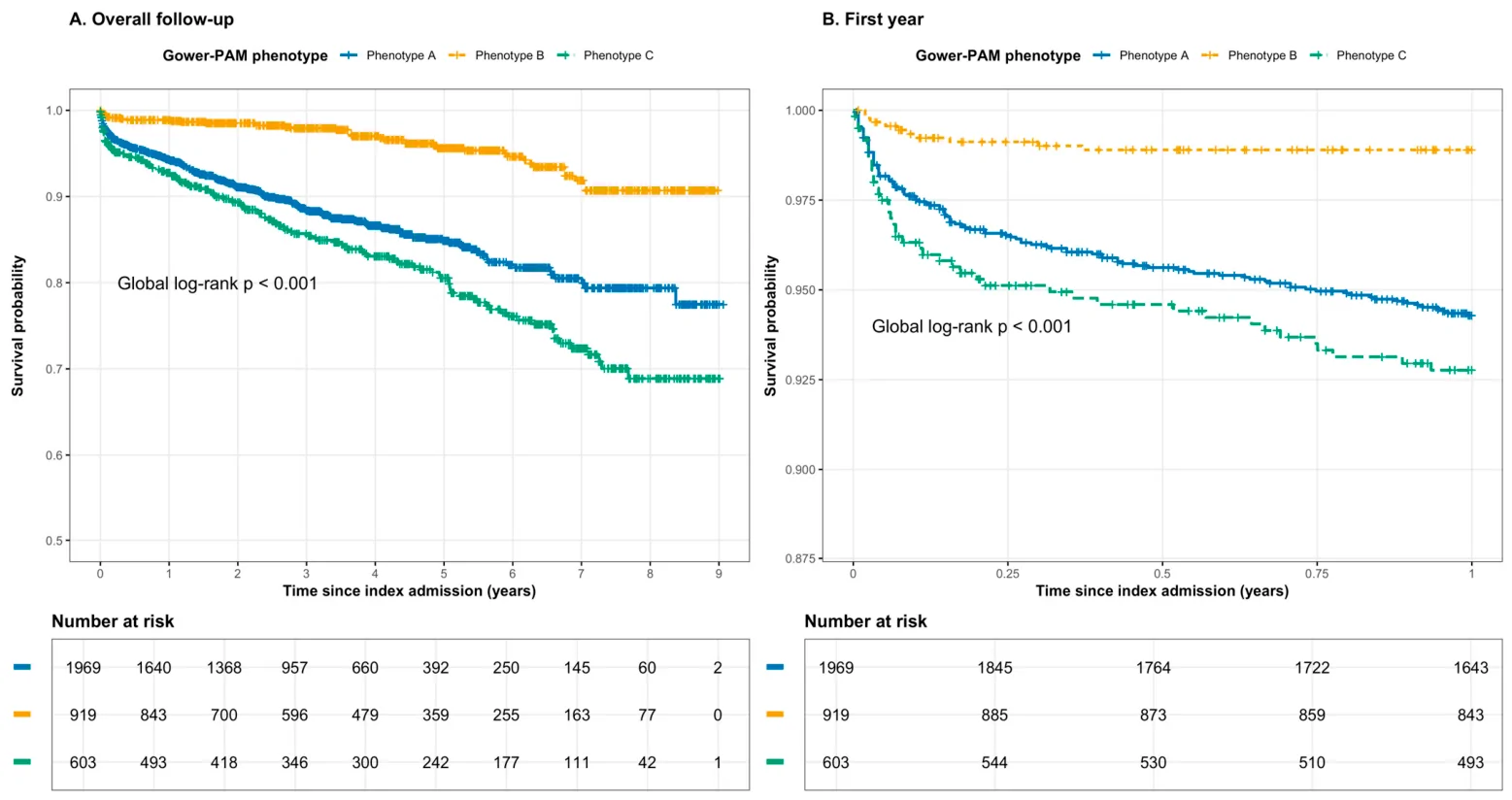
Kaplan–Meier survival curves according to the outcome-independent Gower-PAM phenotypes. The phenotypes were derived from 10 prespecified mixed continuous and categorical baseline variables without using mortality or follow-up information. Panel (**A**) shows survival during overall follow-up, and Panel (**B**) shows survival during the first year. Numbers at risk are presented below each panel, and vertical tick marks indicate censored observations. Survival differed among phenotypes in both panels (global log-rank *p* < 0.001). Phenotype labels were assigned neutrally according to cluster size and do not represent ordered risk categories.

Kaplan–Meier analyses demonstrated progressively worse long-term survival across increasing AAR-3 and CARE-4 burden categories (both log-rank *p* < 0.001; Figure 2). Survival also differed among the three outcome-independent Gower-PAM phenotypes during both overall follow-up and the first year (both global log-rank *p* < 0.001; Figure 3).

Using phenotype A as the reference, the unadjusted overall hazard ratios were 0.28 (95% CI, 0.20–0.39) for phenotype B and 1.35 (95% CI, 1.08–1.68) for phenotype C. In the primary age-inclusive model adjusted for age, sex, acute coronary syndrome subtype, and coronary artery disease history, addition of the phenotype factor significantly improved model fit (joint likelihood-ratio *p* < 0.001). Phenotype B remained associated with lower mortality than phenotype A (HR 0.45, 95% CI, 0.32–0.64; *p* < 0.001), whereas phenotype C did not differ independently from phenotype A (HR 0.92, 95% CI, 0.73–1.16; *p* = 0.494).

Although the proportional hazards test for the phenotype factor was nonsignificant in the primary model (*p* = 0.114), the global model test indicated modest nonproportionality (*p* = 0.011). First-year and landmark estimates were therefore considered the principal time-specific results. For phenotype B versus phenotype A, the adjusted HR was 0.34 (95% CI, 0.18–0.66; *p* = 0.001) during the first year and 0.52 (95% CI, 0.34–0.79; *p* = 0.002) beyond 365 days. Phenotype C was not independently associated with mortality in either period, with adjusted HRs of 0.96 (95% CI, 0.66–1.40; *p* = 0.846) during the first year and 0.90 (95% CI, 0.67–1.22; *p* = 0.493) beyond 365 days. The overall estimates should consequently be interpreted as average associations across follow-up.

In an expanded age-inclusive clinical sensitivity model additionally including diabetes, hypertension, atrial fibrillation, and smoking status, the phenotype factor no longer improved model fit (joint likelihood-ratio *p* = 0.673). The adjusted HRs were 0.97 (95% CI, 0.47–1.98; *p* = 0.922) for phenotype B and 1.21 (95% CI, 0.78–1.88; *p* = 0.385) for phenotype C. This attenuation indicates that the observed prognostic separation was substantially related to the clinical characteristics contributing to the clustering solution and supports a cautious, exploratory interpretation of the phenotypes.

### 3.5. Age-Inclusive Head-to-Head Analysis of AAR-3, CARE-4, and ACEF

The primary head-to-head analysis evaluated AAR-3, CARE-4, and ACEF in the same common complete-case cohort of 3492 patients, in whom 394 deaths occurred. Using the age-inclusive clinical reference model (Model B), AAR-3 was associated with mortality with an adjusted HR of 1.65 per 1-point increase (95% CI, 1.45–1.87), CARE-4 with an adjusted HR of 1.81 per 1-point increase (95% CI, 1.63–2.01), and ACEF with an adjusted HR of 2.48 per 1-unit increase (95% CI, 2.14–2.87; all *p* < 0.001). Harrell’s C-index was 0.770 (95% CI, 0.747–0.792) for the reference model and increased to 0.793 (95% CI, 0.772–0.814) with AAR-3, 0.815 (95% CI, 0.795–0.834) with CARE-4, and 0.812 (95% CI, 0.792–0.833) with ACEF. The corresponding increases in C-index were 0.0231 (95% paired-bootstrap CI, 0.0119–0.0354), 0.0449 (95% CI, 0.0292–0.0603), and 0.0425 (95% CI, 0.0284–0.0567), respectively. The direct difference between the CARE-4- and ACEF-extended models was 0.0024 (95% paired-bootstrap CI, −0.0109 to 0.0150), indicating numerically close discrimination without evidence of a clearly distinguishable difference in this cohort. All likelihood-ratio comparisons with the reference model remained significant (all *p* < 0.001; Table 2).

Because the global proportional-hazards tests were significant in the full-follow-up Model B analyses, the period-specific estimates were treated as the principal effect estimates. During the first year, the analysis included 3492 patients and 161 deaths. The adjusted HR per 1-point increase was 1.78 for AAR-3 (95% CI, 1.46–2.19) and 2.08 for CARE-4 (95% CI, 1.76–2.46), whereas the adjusted HR per 1-unit increase in ACEF was 2.66 (95% CI, 2.14–3.30; all *p* < 0.001). In the 365-day landmark analysis among 2977 patients who remained alive and under observation beyond the first year, with 233 subsequent deaths, the corresponding HRs were 1.56 for AAR-3 (95% CI, 1.32–1.85), 1.66 for CARE-4 (95% CI, 1.44–1.91), and 2.39 for ACEF (95% CI, 1.95–2.92; all *p* < 0.001). The associations persisted beyond the first year, although the estimates were attenuated. No evidence of non-proportionality was identified for the score terms within either time window; complete period-specific effect estimates and variable-level proportional hazards assessments are provided in Supplementary Table S11.

In the continuous-component sensitivity model (Model C), which included age, hemoglobin, eGFR, and LVEF continuously, the reference C-index was 0.828. Addition of AAR-3 (HR, 1.05; 95% CI, 0.87–1.26; *p* = 0.626), CARE-4 (HR, 1.12; 95% CI, 0.96–1.32; *p* = 0.155), or ACEF (HR, 0.90; 95% CI, 0.64–1.26; *p* = 0.551) did not significantly improve model fit. Changes in C-index were also minimal, ranging from −0.0001 to 0.0013. These findings indicate that the simplified scores summarize information contained in their underlying biological domains but do not provide additional prognostic information once those components are modeled continuously.

### 3.6. Sensitivity and Internal Robustness Analyses

**Figure 4:**
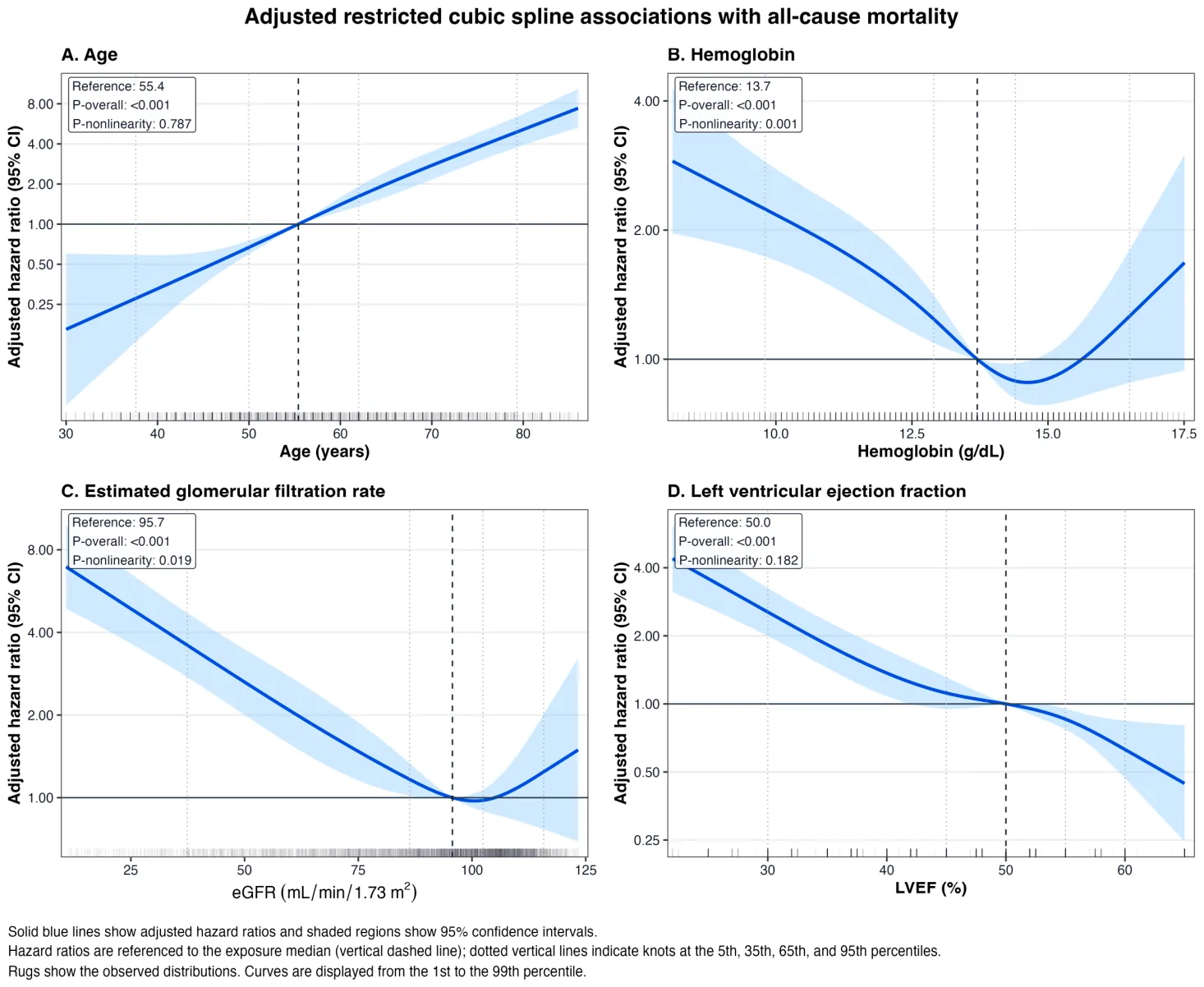
Adjusted restricted cubic spline associations of age, hemoglobin, estimated glomerular filtration rate, and left ventricular ejection fraction with all-cause mortality in the common complete-case cohort (*n* = 3492; 394 deaths). Solid blue lines represent adjusted hazard ratios, and shaded regions represent 95% confidence intervals. Hazard ratios were referenced to the exposure-specific medians: 55.4 years for age, 13.7 g/dL for hemoglobin, 95.7 mL/min/1.73 m^2^ for estimated glomerular filtration rate, and 50% for left ventricular ejection fraction. Vertical dashed lines indicate the reference values, and dotted vertical lines indicate knots placed at the 5th, 35th, 65th, and 95th percentiles. Rug plots show the observed exposure distributions, and curves are displayed from the 1st to the 99th percentile. The age model was adjusted for sex, acute coronary syndrome subtype, diabetes mellitus, hypertension, atrial fibrillation, current smoking, and prior coronary artery disease; the other models were additionally adjusted for age. *p*-values for nonlinearity were 0.787 for age, 0.001 for hemoglobin, 0.019 for estimated glomerular filtration rate, and 0.182 for left ventricular ejection fraction.

Adjusted restricted cubic spline analyses demonstrated significant overall associations of age, hemoglobin, eGFR, and LVEF with all-cause mortality (all overall *p* < 0.001). Evidence of nonlinearity was present for hemoglobin (*p* for nonlinearity = 0.001) and eGFR (*p* for nonlinearity = 0.019), whereas no evidence of nonlinearity was identified for age (*p* = 0.787) or LVEF (*p* = 0.182). The exposure-specific medians used as hazard-ratio references were 55.4 years for age, 13.7 g/dL for hemoglobin, 95.7 mL/min/1.73 m^2^ for eGFR, and 50% for LVEF (Figure 4).

Bootstrap optimism correction and repeated cross-validation showed limited optimism and generally stable discrimination for the framework-specific AAR-3 and CARE-4 models. Detailed validation results are provided in Supplementary Table S15, with the corresponding time-dependent ROC and bootstrap-corrected calibration plots presented in Supplementary Figures S2 and S3. In the multiple-imputation sensitivity analysis, 20 completed datasets were generated using 20 iterations within the 3561-patient survival cohort. The Rubin-pooled adjusted HRs were 1.65 per 1-point increase in AAR-3 (95% CI, 1.45–1.88), 1.81 per 1-point increase in CARE-4 (95% CI, 1.63–2.01), and 2.42 per 1-unit increase in ACEF (95% CI, 2.09–2.81). The corresponding complete-case estimates were 1.65, 1.81, and 2.48, respectively. Mean Harrell C-indices across the imputed datasets were 0.794 for AAR-3, 0.816 for CARE-4, and 0.812 for ACEF, closely corresponding to the complete-case estimates of 0.793, 0.815, and 0.812. Pooled incremental tests remained significant for all three score additions. No imputation-related events were logged, and the convergence diagnostics did not indicate material instability. Detailed convergence diagnostics are presented in Supplementary Figure S1 and Table S8. These findings support robustness to missing covariate data under the missing-at-random assumption but do not address potential selection bias arising from patients excluded because of missing mortality status or invalid follow-up. Complete spline specifications and numerical results are provided in Supplementary Table S12.

The selected three-phenotype Gower-PAM solution was internally stable within the prespecified clustering method, with bootstrap Jaccard similarities of 0.964, 1.000, and 0.893 and no cluster dissolution across 100 resamples. However, agreement with the standardized mixed-variable and continuous-only k-means sensitivity analyses was low, with adjusted Rand indices of 0.049 and 0.026, respectively. Thus, the bootstrap results support within-method stability but not reproducibility of individual phenotype assignments across different clustering algorithms or external cohorts.

The phenotype factor improved the primary age-inclusive model adjusted for age, sex, acute coronary syndrome subtype, and coronary artery disease history. However, only phenotype B differed significantly from phenotype A, whereas phenotype C did not. Furthermore, after diabetes, hypertension, atrial fibrillation, and smoking status were added in the expanded clinical sensitivity model, the joint phenotype effect was no longer significant (likelihood-ratio *p* = 0.673). These findings indicate that the phenotype-associated survival differences substantially overlapped with the underlying clinical characteristics and support an exploratory rather than independently predictive interpretation of the phenomapping results.

In exploratory inflammatory-marker sensitivity analyses, high neutrophil-to-lymphocyte ratio and high systemic immune-inflammation index remained associated with mortality when added separately to CARE-4-based models. These findings suggest that inflammatory indices may contain prognostic information not fully summarized by the CARE-4 components. However, these secondary analyses were not designed to establish incremental clinical utility, causal biological pathways, or an expanded treatment-directing score.

In the internal temporal robustness analysis of the common complete-case cohort, the earlier-period subcohort included 1535 patients and 208 deaths, whereas the later-period subcohort included 1957 patients and 186 deaths. In the 2017–2019 subcohort, AAR-3 (HR per 1-point increase, 1.61; 95% CI, 1.34–1.94), CARE-4 (HR, 1.80; 95% CI, 1.55–2.09), and ACEF (HR per 1-unit increase, 2.66; 95% CI, 2.16–3.27) were independently associated with mortality (all *p* < 0.001). Addition of AAR-3, CARE-4, and ACEF increased the C-index of the reference model from 0.703 to 0.727, 0.762, and 0.767, respectively. Corresponding associations were maintained in the 2020–2024 subcohort for AAR-3 (HR, 1.68; 95% CI, 1.39–2.02), CARE-4 (HR, 1.83; 95% CI, 1.56–2.15), and ACEF (HR, 2.30; 95% CI, 1.86–2.84; all *p* < 0.001). The respective C-indices increased from 0.795 to 0.816, 0.830, and 0.820. Likelihood-ratio tests were significant for all score additions. No evidence of non-proportionality was observed for the score terms or global models. Nominal variable-level departures were observed for ACS subtype in the earlier-period models and for sex in the later-period models; complete Schoenfeld-residual test results are provided in Supplementary Table S13. These findings support the internal temporal robustness of the associations but do not constitute external validation.

Repeated cross-validated survival decision-curve analysis was conducted in the common complete-case cohort of 3492 patients, including 161 deaths within 365 days. The censoring-aware approach retained the 351 patients censored before 365 days. Across the full prespecified threshold range of 1–20%, net-benefit differences were small and not uniformly positive for all expanded models at the lowest thresholds. At 1.5% and 2.0%, the AAR-3-extended model showed essentially similar but slightly lower net benefit than the clinical reference model (Δ net benefit, −0.0001 and −0.0004, respectively), whereas the other expanded models were similar to or above the reference. From 2.5% onward, all models incorporating AAR-3, CARE-4, ACEF, or the continuous biological variables provided greater net benefit than the age-inclusive clinical reference model. At a 5% threshold probability, net benefit was 0.0180 for the reference model, compared with 0.0202 for AAR-3, 0.0227 for CARE-4, 0.0230 for ACEF, and 0.0246 for the continuous biological-domain model. At a 10% threshold, the corresponding values were 0.0064, 0.0092, 0.0111, 0.0121, and 0.0127, respectively. The continuous biological-domain model generally provided the greatest net benefit, whereas CARE-4 retained an advantage over the reference model throughout the evaluated range. These internally cross-validated findings describe exploratory decision-analytic performance and do not establish clinical utility or support use of any threshold for clinical decision-making without independent external validation (Supplementary Table S14 and Figure S4).

## 4. Discussion

### 4.1. Principal Findings

In this single-center retrospective cohort, baseline multidomain characteristics obtained from routine clinical assessment were associated with long-term all-cause mortality after acute coronary syndrome. Outcome-independent Gower-PAM analysis identified three internally stable phenotypic patterns. However, agreement with alternative clustering algorithms was low, and the phenotype effect was attenuated after expanded clinical adjustment. The phenotypes should therefore be interpreted as exploratory descriptions of co-occurring characteristics rather than discrete biological classes or externally reproducible risk groups.

In the common complete-case cohort and against an age-inclusive clinical reference model, AAR-3, CARE-4, and ACEF were each associated with mortality and improved model fit. CARE-4 and ACEF yielded numerically close C-index estimates, and the paired-bootstrap 95% confidence interval for their difference included zero; this finding should not be interpreted as evidence of statistical equivalence. Both frameworks showed larger increases in C-index than AAR-3.

However, none of the scores materially improved a model that already included age, hemoglobin, estimated glomerular filtration rate, and left ventricular ejection fraction as continuous variables. Multiple-imputation, temporal-period, and cross-validated decision-curve analyses supported the internal consistency of the score-based findings, but they do not establish external validity or clinical utility.

### 4.2. Baseline Multidomain Characteristics and Residual Risk After ACS

Despite advances in acute coronary syndrome management and secondary prevention, substantial heterogeneity in long-term outcomes remains after hospital discharge [1,3,25]. Conventional assessment incorporates demographic characteristics, clinical comorbidities, hemodynamic status, anatomical disease burden, and procedural factors. The present findings indicate that baseline renal, hematologic, cardiac functional, inflammatory, and comorbidity-related characteristics also co-occur in clinically distinguishable patterns and are associated with subsequent mortality.

Phenotype C combined older age, lower hemoglobin and estimated glomerular filtration rate, greater inflammatory burden, and high prevalences of diabetes and hypertension and had the highest unadjusted mortality. However, phenotype C did not differ independently from phenotype A after age-inclusive adjustment, and the overall phenotype effect was attenuated in the expanded clinical model. Phenotype B comprised younger patients with lower inflammatory burden and was strongly separated by smoking status. Its lower observed mortality should not be interpreted as a protective association of smoking, because cluster membership simultaneously represented multiple correlated characteristics. These findings demonstrate statistical co-occurrence and prognostic association but do not establish an ordered vulnerability spectrum, a unified biological syndrome, or causal interaction among the included domains.

### 4.3. Biological Context of the Included Domains

The variables included in the phenomapping and score-based analyses have established clinical associations with cardiovascular outcomes. Renal dysfunction has been associated with inflammation, endothelial dysfunction, neurohormonal activation, and increased cardiovascular risk [26,27]. Anemia may reflect impaired oxygen delivery, comorbidity burden, and reduced physiological reserve in patients with acute coronary syndromes [28,29]. Reduced left ventricular ejection fraction indicates impaired cardiac function, while inflammatory indices such as the neutrophil-to-lymphocyte ratio and systemic immune-inflammation index have been associated with adverse cardiovascular outcomes [6,8,21,30,31]. These routinely available measurements may therefore provide complementary information regarding the baseline condition of patients hospitalized with acute coronary syndrome.

These established associations provide biological context for the observed statistical patterns but do not demonstrate the mechanisms responsible for mortality in the present cohort. The study did not directly measure inflammatory pathways, physiological reserve, frailty, interactions among biological domains, or longitudinal changes in these variables. Accordingly, the clustering results should be interpreted as showing the co-occurrence of baseline characteristics rather than proving that the included abnormalities form a single pathophysiological entity. Prospective studies incorporating direct frailty measures, serial biomarker assessment, and cause-specific outcomes would be required to examine the proposed biological relationships more directly.

### 4.4. Phenomapping and Data-Driven Cardiovascular Phenotyping

Phenomapping approaches have been used to describe heterogeneity in cardiovascular populations, particularly in heart failure and multimorbidity settings [13,14,15,16,32]. In the present study, Gower-PAM clustering was used as an exploratory descriptive method rather than as a prediction algorithm or treatment-directing tool. The three-phenotype solution was internally stable within the selected method, but its low agreement with alternative clustering approaches indicates that individual phenotype assignments were sensitive to analytical choices. Consequently, the phenotypes should not be regarded as fixed or externally reproducible patient classes.

AAR-3 and CARE-4 were developed post hoc after examination of the dominant domains represented in the phenomapping analysis and were evaluated in the same institutional cohort. Their equal weighting and clinically familiar thresholds were selected for simplicity and interpretability rather than derived through statistical optimization. This creates partial circularity and increases the possibility of optimistic performance estimates. AAR-3 and CARE-4 should therefore be considered exploratory vulnerability-burden summaries rather than validated prediction scores.

In the age-inclusive common complete-case analysis, CARE-4 improved model fit and discrimination compared with the clinical reference model. Its C-index was numerically close to that of ACEF (0.815 versus 0.812), with a paired-bootstrap difference of 0.0024 (95% CI, −0.0109 to 0.0150). This interval included zero but should not be interpreted as demonstrating statistical equivalence between the two frameworks.

However, CARE-4 did not materially improve a model that already included age, hemoglobin, estimated glomerular filtration rate, and left ventricular ejection fraction continuously. Cross-validated decision-curve analysis similarly showed that the continuous-domain model generally provided the greatest net benefit. These findings suggest that CARE-4 may summarize information contained in its underlying domains, but they do not demonstrate additional biological information or clinical utility beyond comprehensive continuous-variable modeling. Any potential role as a simplified communication or risk-enrichment framework requires prospective external validation.

### 4.5. Clinical Implications for Post-ACS Care

The present study did not evaluate a management strategy based on AAR-3, CARE-4, or Gower-PAM phenotype membership. It therefore cannot determine whether closer surveillance, cardiac rehabilitation, medication optimization, additional laboratory monitoring, or altered resource allocation would improve outcomes in patients with higher vulnerability-burden scores. No score threshold was linked prospectively to a clinical action, and treatment effects were not assessed. These approaches should not replace guideline-directed assessment or established acute coronary syndrome risk tools.

Direct comparison with GRACE was not possible because several required variables, including Killip class and standardized admission hemodynamic measurements, were not systematically available in the retrospective records. ACEF was therefore included as an available established comparator, but this does not establish equivalence to a comprehensive acute coronary syndrome risk model.

The clinical implications of the present findings are consequently limited to hypothesis generation. Prospective multicenter studies should externally evaluate discrimination, calibration, and clinical net benefit; compare the proposed frameworks directly with established acute coronary syndrome scores; and determine whether their performance remains stable across different populations and treatment practices. Only after such validation should interventional studies examine whether score-informed follow-up pathways improve patient outcomes or resource use.

### 4.6. Early and Late Associations

In the age-inclusive common-cohort analyses, AAR-3, CARE-4, and ACEF were associated with mortality during both the first year and the period beyond 365 days. During the first year, the adjusted HRs were 1.78 per 1-point increase in AAR-3, 2.08 per 1-point increase in CARE-4, and 2.66 per 1-unit increase in ACEF. The corresponding landmark HRs were 1.56, 1.66, and 2.39, respectively. The estimates were numerically larger during the first year, with more evident attenuation for AAR-3 and CARE-4 beyond 365 days. This pattern may indicate that the baseline domains summarized by these frameworks are particularly relevant during the early period after acute coronary syndrome. However, the analyses did not evaluate the biological mechanisms responsible for the temporal differences, and the estimates from the two time windows should not be interpreted as directly comparable causal effects.

The Gower-PAM results did not support a simple ordered phenotype risk gradient. Phenotype B was associated with lower mortality than phenotype A during both periods, whereas phenotype C did not differ independently from phenotype A in either the first-year or landmark analysis. These findings further support treating the phenotypes as exploratory patterns of co-occurring characteristics rather than as low-, intermediate-, and high-risk biological states.

Because the proportional hazards assumption was not fully satisfied for all model covariates, the first-year and landmark estimates were emphasized instead of relying solely on an average overall HR. The landmark estimates apply only to patients who remained alive and under observation beyond 365 days and should therefore not be interpreted as directly comparable causal effects in an unchanged population. Furthermore, all vulnerability-related variables were measured at baseline; no serial measurements were available to determine whether individual patient characteristics or risk states changed during follow-up.

### 4.7. Strengths and Limitations

This study included a large consecutive real-world cohort of patients hospitalized with acute coronary syndrome and had a median follow-up exceeding 3 years. The analyses incorporated routine hematologic, renal, inflammatory, cardiac functional, and clinical variables. Methodological strengths included outcome-independent mixed-data clustering, prespecified quantitative cluster-selection criteria, bootstrap assessment of cluster stability, age-inclusive common-cohort model comparisons, multiple imputation, time-specific survival analyses, and cross-validated decision-curve analysis.

Several limitations should nevertheless be acknowledged. 

First, this was a retrospective observational study conducted at a single tertiary cardiovascular center in Türkiye. Residual confounding cannot be excluded, and the findings may not generalize to hospitals with different patient characteristics, treatment practices, follow-up systems, or resource settings.

Second, 240 records were excluded from the survival cohort because mortality status or valid follow-up information was unavailable. The excluded group was older, included a higher proportion of women, had lower estimated glomerular filtration rates, and had higher prevalences of diabetes and hypertension than the included group. The exclusions were therefore unlikely to have occurred completely at random and may have preferentially removed higher-risk patients. Multiple imputation addressed missing covariate data within the survival cohort but could not correct potential selection bias resulting from missing outcome or follow-up information.

Third, patients without a recorded death were known to be alive through their last documented follow-up assessment and were censored at that time. Vital status after that date was unknown. Although mortality was assessed using hospital electronic records and the national health information system, informative censoring cannot be excluded. In addition, the landmark analysis beyond 365 days was restricted to patients who survived and remained under observation through the first year and is therefore subject to survivor-selection effects.

Fourth, treatment practices may have changed during the 2017–2024 study period. Detailed information on revascularization completeness, procedural complexity, discharge pharmacotherapy, medication adherence, lipid-lowering intensity, cardiac rehabilitation participation, and changes in treatment during follow-up was not comprehensively available. The earlier-versus-later period analysis was conducted within the same institution and should be interpreted as a temporal robustness assessment rather than external validation. Unmeasured treatment differences and secular changes may have contributed to the observed associations.

Fifth, the endpoint was all-cause mortality. Cause-specific mortality and nonfatal cardiovascular events were not systematically available, preventing evaluation of whether the associations were driven primarily by cardiovascular or noncardiovascular outcomes. Consequently, the findings describe associations with overall prognosis and cannot identify specific disease mechanisms.

Sixth, the analyses used measurements obtained during the index hospitalization. Left ventricular ejection fraction was obtained within the first 24–48 h but may have been influenced by acute loading conditions, image quality, and interobserver or intraobserver variability. Laboratory and echocardiographic measurements were not repeated longitudinally. Direct measures of frailty, functional capacity, nutritional status, cognition, or sarcopenia were also unavailable. References to physiological reserve or frailty should therefore be considered interpretive clinical context rather than directly measured study findings.

Seventh, phenomapping was exploratory. Although the selected Gower-PAM solution was stable within the applied method, agreement with the mixed-variable and continuous-only k-means sensitivity analyses was low. Equal unit weighting should not be interpreted as implying equal empirical influence of all clustering variables, because their distributions and prevalences may differentially affect the resulting partition. Smoking status strongly influenced the selected partition, and phenotype B consisted entirely of patients recorded as smokers. Its lower observed mortality must not be interpreted as a protective effect of smoking and likely reflects the younger age and different distribution of other baseline characteristics in that phenotype [33,34]. More generally, the three phenotypes should not be interpreted as definitive biological classes, ordered risk groups, or mechanistically established disease subtypes.

Eighth, AAR-3 and CARE-4 were constructed post hoc from domains examined in the same cohort and were evaluated internally. Their apparent performance may therefore be optimistic, despite the common-cohort, multiple-imputation, bootstrap, and cross-validation analyses. Direct comparison with GRACE was not possible because all variables required for its calculation were not systematically available. The scores should not be considered validated prediction tools or treatment-directing algorithms.

Finally, no independent external validation cohort was available. Bootstrap resampling, repeated cross-validation, multiple imputation, and analyses across two study periods assess internal consistency but cannot establish transportability to other institutions. Prospective multicenter studies should externally validate the score-based findings, evaluate calibration and clinical net benefit, compare the frameworks with established acute coronary syndrome risk models, and determine whether any score-informed management strategy improves patient outcomes.

## 5. Conclusions

In this single-center retrospective cohort of patients hospitalized with acute coronary syndrome, routinely available baseline demographic, hematologic, renal, inflammatory, cardiac functional, and clinical characteristics were associated with long-term all-cause mortality. Outcome-independent Gower-PAM analysis identified three internally stable patterns of co-occurring characteristics. However, phenotype membership was sensitive to the clustering method, and the overall phenotype effect was attenuated after expanded clinical adjustment. The phenotypes should therefore be interpreted as exploratory descriptive patterns rather than independently predictive biological classes or ordered risk groups.

In the age-inclusive common complete-case cohort, AAR-3, CARE-4, and ACEF were each associated with mortality and improved model fit. CARE-4 and ACEF yielded numerically close discrimination estimates in this cohort, although the analysis was not designed to establish statistical equivalence. None of the scores materially improved a model that already included their underlying continuous biological domains. AAR-3 and CARE-4 were developed post hoc and should be considered exploratory risk-enrichment summaries rather than validated clinical prediction tools. Independent prospective multicenter external validation and comparison with established acute coronary syndrome risk models are required before either framework can be considered for clinical risk stratification, treatment decisions, or other clinical implementation.

## Figures and Tables

**Table 1:** Baseline characteristics across CARE-4 burden categories.

Variable	Overall *n* = 3505	CARE-4 = 0 *n*= 1842	CARE-4 = 1 *n* = 1047	CARE-4 = 2 *n* = 436	CARE-4 = 3 *n* = 145	CARE-4 = 4 *n* = 35	*p*-Value
Age, years	56.94 (12.98)	51.70 (10.08)	58.27 (11.84)	68.03 (12.46)	74.91 (10.33)	80.84 (3.55)	<0.001
Female sex, *n* (%)	841 (24.0)	286 (15.5)	302 (28.8)	168 (38.5)	67 (46.2)	18 (51.4)	<0.001
ACS type, *n* (%)							<0.001
NSTEMI	1031 (29.4)	500 (27.2)	295 (28.2)	167 (38.3)	53 (36.6)	16 (45.7)	
STEMI	1994 (56.9)	1059 (57.5)	614 (58.6)	229 (52.5)	76 (52.4)	16 (45.7)	
Unstable angina	479 (13.7)	282 (15.3)	138 (13.2)	40 (9.2)	16 (11.0)	3 (8.6)	
Diabetes mellitus, *n* (%)	618 (17.6)	283 (15.4)	173 (16.5)	114 (26.1)	41 (28.3)	7 (20.0)	<0.001
Hypertension, *n* (%)	917 (26.2)	441 (24.0)	251 (24.0)	162 (37.2)	49 (33.8)	14 (40.0)	<0.001
Atrial fibrillation, *n* (%)	89 (2.5)	25 (1.4)	23 (2.2)	31 (7.1)	8 (5.5)	2 (5.7)	<0.001
Current smoking, *n* (%)	1060 (30.3)	738 (40.1)	241 (23.1)	64 (14.7)	14 (9.7)	3 (8.6)	<0.001
Prior coronary artery disease, *n* (%)	894 (25.5)	399 (21.7)	279 (26.7)	153 (35.1)	48 (33.1)	15 (42.9)	0.010
Hemoglobin, g/dL	13.70 [12.20–14.90]	14.50 [13.70–15.40]	12.60 [11.40–14.10]	11.50 [10.40–12.70]	11.00 [9.90–11.80]	11.10 [10.25–11.85]	<0.001
Creatinine, mg/dL	0.86 [0.76–1.06]	0.84 [0.76–0.97]	0.84 [0.74–1.00]	1.15 [0.81–1.58]	1.58 [1.13–1.96]	1.33 [1.19–1.64]	<0.001
eGFR, mL/min/1.73 m^2^	95.70 [76.00–106.20]	101.20 [89.90–108.70]	94.40 [76.40–105.25]	59.25 [43.32–88.00]	43.00 [29.70–54.90]	44.70 [37.70–52.75]	<0.001
Glucose, mg/dL	123.00 [100.00–164.00]	119.00 [98.25–156.00]	124.00 [102.00–170.00]	133.00 [102.00–181.00]	137.00 [107.00–185.00]	128.50 [104.75–182.75]	<0.001
NLR	2.87 [1.88–4.64]	2.47 [1.69–3.76]	3.14 [2.03–4.95]	3.96 [2.58–6.27]	4.78 [3.26–7.45]	3.48 [2.47–5.72]	<0.001
SII	701.73 [446.97–1197.08]	604.89 [414.52–954.25]	793.37 [469.90–1273.00]	968.19 [591.58–1544.61]	1233.27 [748.98–1910.89]	820.62 [480.75–1278.44]	<0.001
LVEF, %	50.00 [42.50–60.00]	55.00 [48.00–60.00]	50.00 [37.50–55.00]	45.00 [35.00–55.00]	40.00 [35.00–50.00]	35.00 [30.00–35.00]	<0.001
All-cause death, *n* (%)	397 (11.3)	63 (3.4)	131 (12.5)	121 (27.8)	64 (44.1)	18 (51.4)	<0.001
Follow-up duration, years	3.33 [1.77–5.27]	3.89 [2.14–5.78]	3.18 [1.74–4.94]	2.37 [1.02–4.31]	1.73 [0.35–3.62]	1.26 [0.28–3.56]	<0.001

**Footnote:** Values are presented as mean (standard deviation), median [interquartile range], or number (%), as appropriate. *p*-values were calculated using one-way analysis of variance or Kruskal–Wallis test for continuous variables and Pearson’s chi-square test for categorical variables. CARE-4 was calculated as the sum of age ≥ 75 years, WHO-defined anemia, estimated glomerular filtration rate < 60 mL/min/1.73 m^2^ and left ventricular ejection fraction < 40%, with each component contributing 1 point. Prior coronary artery disease was defined as any recorded nonzero coronary artery disease history category. Acute coronary syndrome subtype was missing in one patient; percentages were calculated using available data. Because age, anemia/hemoglobin, eGFR, and LVEF are components of CARE-4, comparisons and *p*-values for these variables are descriptive and should not be interpreted as inferential tests independent of score construction. The CARE-4 = 4 category contained only 35 patients and should be interpreted cautiously. **Abbreviations:** ACS, acute coronary syndrome; eGFR, estimated glomerular filtration rate; LVEF, left ventricular ejection fraction; NLR, neutrophil-to-lymphocyte ratio; NSTEMI, non-ST-segment elevation myocardial infarction; SII, systemic immune-inflammation index; STEMI, ST-segment elevation myocardial infarction.

**Table 2:** Age-inclusive head-to-head comparison of AAR-3, CARE-4, and ACEF for all-cause mortality in the common complete-case cohort (*n* = 3492; 394 deaths).

Model/Score Addition	Adjusted HR (95% CI)	*p*-Value	Harrell’s C-Index (95% CI)	∆ C-Index	AIC	BIC	Likelihood-Ratio *p*-Value
Panel A. Primary analysis: Model B age-inclusive clinical reference							
Reference model	-	-	0.770 (0.747–0.792)	0.0000	5680.2	5715.9	-
+ AAR-3	1.65 (1.45–1.87)	<0.001	0.793 (0.772–0.814)	0.0231 (0.0119–0.0354)	5625.4	5665.2	<0.001
+ CARE-4	1.81 (1.63–2.01)	<0.001	0.815 (0.795–0.834)	0.0449 (0.0292–0.0603)	5568.8	5608.6	<0.001
+ ACEF	2.48 (2.14–2.87)	<0.001	0.812 (0.792–0.833)	0.0425 (0.0284–0.0567)	5565.7	5605.5	<0.001
Panel B. Continuous-component sensitivity analysis: Model C							
Reference model	-	-	0.828 (0.808–0.847)	0.0000	5500.6	5548.3	-
+ AAR-3	1.05 (0.87–1.26)	0.626	0.828 (0.809–0.847)	0.0003	5502.4	5554.0	0.626
+ CARE-4	1.12 (0.96–1.32)	0.155	0.829 (0.810–0.848)	0.0013	5500.6	5552.3	0.156
+ ACEF	0.90 (0.64–1.26)	0.551	0.827 (0.808–0.847)	−0.0001	5502.2	5553.9	0.550

**Footnote:** All models were fitted in the same common complete-case cohort. Hazard ratios for AAR-3 and CARE-4 are reported per 1-point increase, whereas the ACEF hazard ratio is reported per 1-unit increase. Model B included age, sex, ACS subtype, diabetes mellitus, hypertension, atrial fibrillation, current smoking, and history of coronary artery disease. Model C included all Model B covariates together with continuous hemoglobin, estimated glomerular filtration rate, and left ventricular ejection fraction. Harrell’s C-index is presented with its model-based 95% confidence interval. For the primary Model B comparisons, values in parentheses accompanying Δ C-index represent 95% confidence intervals obtained from 2000 paired patient-level nonparametric bootstrap resamples. Δ C-index values for the secondary Model C sensitivity analysis are presented as point estimates only. The paired-bootstrap difference between the CARE-4- and ACEF-extended Model B models was 0.0024 (95% CI, −0.0109 to 0.0150); this interval should not be interpreted as establishing statistical equivalence. Likelihood-ratio p-values compare each score-extended model with its corresponding reference model. Lower AIC and BIC values indicate better model fit. Because the global proportional-hazards assumption was not satisfied over the complete follow-up period, these overall hazard ratios represent average associations over time; the principal first-year and landmark estimates are reported in the text and Supplementary Table S11. **Abbreviations:** ACEF, Age, Creatinine, and Ejection Fraction; AIC, Akaike information criterion; BIC, Bayesian information criterion; CI, confidence interval; eGFR, estimated glomerular filtration rate; HR, hazard ratio; LVEF, left ventricular ejection fraction.

## Data Availability

The de-identified data supporting the findings of this study are not publicly available because of institutional and ethical restrictions governing patient-level clinical data. The data may be made available by the corresponding author upon reasonable request, subject to institutional approval and compliance with applicable data-protection regulations. The analytical code may be made available under the same conditions.

## Supplementary Materials

The following supporting information can be downloaded at https://www.mdpi.com/article/10.3390/medicina62081586/s1: Figure S1: Multiple-imputation convergence trace plots; Figure S2: Time-dependent ROC curves for 1-year mortality for the framework-specific AAR-3 and CARE-4 models; Figure S3: Bootstrap-corrected calibration plots for the framework-specific AAR-3 and CARE-4 models; Figure S4: Cross-validated survival decision curves for 1-year all-cause mortality; Table S1: Variable-level missingness in the initial and survival-analysis cohorts; Table S2: Comparison of baseline characteristics between patients included in and excluded from the survival analysis; Table S3: Baseline characteristics according to AAR-3 burden categories; Table S4: Outcome-independent Gower–PAM cluster-number selection, cluster sizes, and bootstrap stability; Table S5: Characteristics and post-clustering clinical outcomes of the selected Gower–PAM phenotypes; Table S6: Survival associations, proportional hazards assessments, and sensitivity analyses for the Gower–PAM phenotypes; Table S7: Selected univariable Cox proportional hazards analyses for long-term all-cause mortality; Table S8: Multiple-imputation input missingness, pooled score effects, model performance, incremental tests, and convergence diagnostics; Table S9: Full common-cohort hierarchical comparison of AAR-3, CARE-4, and ACEF, including effect estimates, discrimination, model fit, and incremental tests; Table S10: Variable-level proportional hazards assessments for the primary full-follow-up common-cohort models; Table S11: First-year and >365-day landmark associations, discrimination, and proportional hazards assessments; Table S12: Specifications and results of the adjusted restricted cubic spline analyses; Table S13: Internal temporal-period robustness analyses and proportional hazards assessments in the common complete-case cohort; Table S14: Cross-validated survival decision-curve results at selected threshold probabilities and distribution of out-of-fold predicted risks; Table S15: Internal validation of the framework-specific AAR-3 and CARE-4 models; File S1: The STROBE reporting checklist; File S2: TRIPOD-AI checklist.

## References

[1] Byrne R.A., Rossello X., Coughlan J.J., Barbato E., Berry C., Chieffo A., Claeys M.J., Dan G.A., Dweck M.R., Galbraith M. (2023). 2023 ESC Guidelines for the management of acute coronary syndromes. Eur. Heart J..

[2] Ridker P.M. (2020). From CANTOS to CIRT to COLCOT to Clinic: Will All Atherosclerosis Patients Soon Be Treated With Combination Lipid-Lowering and Inflammation-Inhibiting Agents?. Circulation.

[3] Lawler P.R., Bhatt D.L., Godoy L.C., Luscher T.F., Bonow R.O., Verma S., Ridker P.M. (2021). Targeting cardiovascular inflammation: Next steps in clinical translation. Eur. Heart J..

[4] Damman K., Valente M.A., Voors A.A., O’Connor C.M., van Veldhuisen D.J., Hillege H.L. (2014). Renal impairment, worsening renal function, and outcome in patients with heart failure: An updated meta-analysis. Eur. Heart J..

[5] Anand A., Cudmore S., Robertson S., Stephen J., Haga K., Weir C.J., Murray S.A., Boyd K., Gunn J., Iqbal J. (2020). Frailty assessment and risk prediction by GRACE score in older patients with acute myocardial infarction. BMC Geriatr..

[6] Angkananard T., Anothaisintawee T., McEvoy M., Attia J., Thakkinstian A. (2018). Neutrophil Lymphocyte Ratio and Cardiovascular Disease Risk: A Systematic Review and Meta-Analysis. Biomed. Res. Int..

[7] Kiris T., Celik A., Varis E., Akan E., Akyildiz Z.I., Karaca M., Nazli C., Dogan A. (2017). Association of Lymphocyte-to-Monocyte Ratio with the Mortality in Patients with ST-Elevation Myocardial Infarction Who Underwent Primary Percutaneous Coronary Intervention. Angiology.

[8] Yang Y.L., Wu C.H., Hsu P.F., Chen S.C., Huang S.S., Chan W.L., Lin S.J., Chou C.Y., Chen J.W., Pan J.P. (2020). Systemic immune-inflammation index (SII) predicted clinical outcome in patients with coronary artery disease. Eur. J. Clin. Investig..

[9] Silverberg D.S., Wexler D., Blum M., Iaina A. (2003). The cardio renal anemia syndrome: Correcting anemia in patients with resistant congestive heart failure can improve both cardiac and renal function and reduce hospitalizations. Clin. Nephrol..

[10] Fox K.A., Dabbous O.H., Goldberg R.J., Pieper K.S., Eagle K.A., Van de Werf F., Avezum A., Goodman S.G., Flather M.D., Anderson F.A. (2006). Prediction of risk of death and myocardial infarction in the six months after presentation with acute coronary syndrome: Prospective multinational observational study (GRACE). BMJ.

[11] Ibanez B., James S., Agewall S., Antunes M.J., Bucciarelli-Ducci C., Bueno H., Caforio A.L.P., Crea F., Goudevenos J.A., Halvorsen S. (2018). 2017 ESC Guidelines for the management of acute myocardial infarction in patients presenting with ST-segment elevation: The Task Force for the management of acute myocardial infarction in patients presenting with ST-segment elevation of the European Society of Cardiology (ESC). Eur. Heart J..

[12] McKechnie D.G.J., Papacosta A.O., Lennon L.T., Ramsay S.E., Whincup P.H., Wannamethee S.G. (2021). Associations between inflammation, cardiovascular biomarkers and incident frailty: The British Regional Heart Study. Age Ageing.

[13] Shah S.J., Katz D.H., Selvaraj S., Burke M.A., Yancy C.W., Gheorghiade M., Bonow R.O., Huang C.C., Deo R.C. (2015). Phenomapping for novel classification of heart failure with preserved ejection fraction. Circulation.

[14] Segar M.W., Patel K.V., Ayers C., Basit M., Tang W.H.W., Willett D., Berry J., Grodin J.L., Pandey A. (2020). Phenomapping of patients with heart failure with preserved ejection fraction using machine learning-based unsupervised cluster analysis. Eur. J. Heart Fail..

[15] Ahmad T., Pencina M.J., Schulte P.J., O’Brien E., Whellan D.J., Pina I.L., Kitzman D.W., Lee K.L., O’Connor C.M., Felker G.M. (2014). Clinical implications of chronic heart failure phenotypes defined by cluster analysis. J. Am. Coll. Cardiol..

[16] Deo R.C. (2015). Machine Learning in Medicine. Circulation.

[17] Thygesen K., Alpert J.S., Jaffe A.S., Chaitman B.R., Bax J.J., Morrow D.A., White H.D., ESC Scientific Document Group (2019). Fourth universal definition of myocardial infarction (2018). Eur. Heart J..

[18] Inker L.A., Eneanya N.D., Coresh J., Tighiouart H., Wang D., Sang Y., Crews D.C., Doria A., Estrella M.M., Froissart M. (2021). New Creatinine- and Cystatin C–Based Equations to Estimate GFR without Race. N. Engl. J. Med..

[19] World Health Organization (2024). Guideline on Haemoglobin Cutoffs to Define Anaemia in Individuals and Populations.

[20] (2024). Kidney Disease: Improving Global Outcomes, C.K.D.W.G. KDIGO 2024 Clinical Practice Guideline for the Evaluation and Management of Chronic Kidney Disease. Kidney Int..

[21] McDonagh T.A., Metra M., Adamo M., Gardner R.S., Baumbach A., Bohm M., Burri H., Butler J., Celutkiene J., Chioncel O. (2022). 2021 ESC Guidelines for the diagnosis and treatment of acute and chronic heart failure: Developed by the Task Force for the diagnosis and treatment of acute and chronic heart failure of the European Society of Cardiology (ESC). With the special contribution of the Heart Failure Association (HFA) of the ESC. Eur. J. Heart Fail..

[22] Ranucci M., Castelvecchio S., Menicanti L., Frigiola A., Pelissero G. (2009). Risk of assessing mortality risk in elective cardiac operations: Age, creatinine, ejection fraction, and the law of parsimony. Circulation.

[23] von Elm E., Altman D.G., Egger M., Pocock S.J., Gotzsche P.C., Vandenbroucke J.P., Initiative S. (2014). The Strengthening the Reporting of Observational Studies in Epidemiology (STROBE) Statement: Guidelines for reporting observational studies. Int. J. Surg..

[24] Collins G.S., Moons K.G.M., Dhiman P., Riley R.D., Beam A.L., Van Calster B., Ghassemi M., Liu X., Reitsma J.B., van Smeden M. (2024). TRIPOD+AI statement: Updated guidance for reporting clinical prediction models that use regression or machine learning methods. BMJ.

[25] Ridker P.M. (2016). Residual inflammatory risk: Addressing the obverse side of the atherosclerosis prevention coin. Eur. Heart J..

[26] Jankowski J., Floege J., Fliser D., Bohm M., Marx N. (2021). Cardiovascular Disease in Chronic Kidney Disease: Pathophysiological Insights and Therapeutic Options. Circulation.

[27] Matsushita K., van der Velde M., Astor B.C., Woodward M., Levey A.S., de Jong P.E., Coresh J., Gansevoort R.T., Chronic Kidney Disease Prognosis Consortium (2010). Association of estimated glomerular filtration rate and albuminuria with all-cause and cardiovascular mortality in general population cohorts: A collaborative meta-analysis. Lancet.

[28] Kunadian V., Mehran R., Lincoff A.M., Feit F., Manoukian S.V., Hamon M., Cox D.A., Dangas G.D., Stone G.W. (2014). Effect of anemia on frequency of short- and long-term clinical events in acute coronary syndromes (from the Acute Catheterization and Urgent Intervention Triage Strategy Trial). Am. J. Cardiol..

[29] Farhan S., Baber U., Mehran R. (2016). Anemia and Acute Coronary Syndrome: Time for Intervention Studies. J. Am. Heart Assoc..

[30] Dentali F., Nigro O., Squizzato A., Gianni M., Zuretti F., Grandi A.M., Guasti L. (2018). Impact of neutrophils to lymphocytes ratio on major clinical outcomes in patients with acute coronary syndromes: A systematic review and meta-analysis of the literature. Int. J. Cardiol..

[31] Adamstein N.H., MacFadyen J.G., Rose L.M., Glynn R.J., Dey A.K., Libby P., Tabas I.A., Mehta N.N., Ridker P.M. (2021). The neutrophil-lymphocyte ratio and incident atherosclerotic events: Analyses from five contemporary randomized trials. Eur. Heart J..

[32] Krittanawong C., Virk H.U.H., Bangalore S., Wang Z., Johnson K.W., Pinotti R., Zhang H., Kaplin S., Narasimhan B., Kitai T. (2020). Machine learning prediction in cardiovascular diseases: A meta-analysis. Sci. Rep..

[33] Gupta T., Kolte D., Khera S., Harikrishnan P., Mujib M., Aronow W.S., Jain D., Ahmed A., Cooper H.A., Frishman W.H. (2016). Smoker’s Paradox in Patients with ST-Segment Elevation Myocardial Infarction Undergoing Primary Percutaneous Coronary Intervention. J. Am. Heart Assoc..

[34] Redfors B., Furer A., Selker H.P., Thiele H., Patel M.R., Chen S., Udelson J.E., Ohman E.M., Eitel I., Granger C.B. (2020). Effect of Smoking on Outcomes of Primary PCI in Patients with STEMI. J. Am. Coll. Cardiol..

