# Supplementary material for: Exploratory Mixed-Data Phenomapping and Risk-Enrichment Frameworks for Long-Term Mortality After Acute Coronary Syndrome: A Single-Center Cohort Study
Source: Medicina (Kaunas). 2026 Aug 18;62(8):1586. doi: 10.3390/medicina62081586 (PMC13515590; doi:10.3390/medicina62081586)
Supplement: Supplementary file 1 [file medicina-62-01586-s001.zip › Supplementary figures and tables.pdf]

Supplementary Materials

Supplementary Tables

Supplementary Table S1. Variable-level missingness in the initial and survival-analysis cohorts

Supplementary Table S2. Comparison of baseline characteristics between patients included in and excluded from the survival analysis

Supplementary Table S3. Baseline characteristics according to AAR-3 burden categories

Supplementary Table S4. Outcome-independent Gower–PAM cluster-number selection, cluster sizes, and bootstrap stability

Supplementary Table S5. Characteristics and post-clustering clinical outcomes of the selected Gower–PAM phenotypes

Supplementary Table S6. Survival associations, proportional hazards assessments, and sensitivity analyses for the Gower–PAM phenotypes

Supplementary Table S7. Selected univariable Cox proportional hazards analyses for long-term all-cause mortality

Supplementary Table S8. Multiple-imputation input missingness, pooled score effects, model performance, incremental tests, and convergence diagnostics

Supplementary Table S9. Full common-cohort hierarchical comparison of AAR-3, CARE-4, and ACEF, including effect estimates, discrimination, model fit, and incremental tests

Supplementary Table S10. Variable-level proportional hazards assessments for the primary full-follow-up common-cohort models

Supplementary Table S11. First-year and >365-day landmark associations, discrimination, and proportional hazards assessments

Supplementary Table S12. Specifications and results of the adjusted restricted cubic spline analyses

Supplementary Table S13. Internal temporal-period robustness analyses and proportional hazards assessments in the common complete-case cohort

Supplementary Table S14. Cross-validated survival decision-curve results at selected threshold probabilities and distribution of out-of-fold predicted risks

Supplementary Table S15. Internal validation of the framework-specific AAR-3 and CARE-4 models

Supplementary Figures

Supplementary Figure S1. Multiple-imputation convergence trace plots

Supplementary Figure S2. Time-dependent ROC curves for 1-year mortality for the framework-specific AAR-3 and CARE-4 models

Supplementary Figure S3. Bootstrap-corrected calibration plots for the framework-specific AAR-3 and CARE-4 models

Supplementary Figure S4. Cross-validated survival decision curves for 1-year all-cause mortality

Supplementary Table S1. Variable-level missingness in the initial and survival-analysis cohorts

Variable	Initial cohort (n = 3801), available n	Initial cohort, missing n (%)	Survival cohort (n = 3561), available n	Survival cohort, missing n (%)
Mortality status	3658	143 (3.76)	3561	0 (0.00)
Follow-up duration	3747	54 (1.42)	3561	0 (0.00)
Age	3783	18 (0.47)	3560	1 (0.03)
Sex	3801	0 (0.00)	3561	0 (0.00)
ACS subtype	3799	2 (0.05)	3560	1 (0.03)
Diabetes mellitus	3793	8 (0.21)	3558	3 (0.08)
Hypertension	3793	8 (0.21)	3558	3 (0.08)
Atrial fibrillation	3794	7 (0.18)	3558	3 (0.08)
Current smoking	3786	15 (0.39)	3553	8 (0.22)
Coronary artery disease history	3795	6 (0.16)	3558	3 (0.08)
Creatinine	3799	2 (0.05)	3560	1 (0.03)
eGFR (CKD-EPI)	3779	22 (0.58)	3559	2 (0.06)
Hemoglobin	3796	5 (0.13)	3558	3 (0.08)
WHO-defined anemia	3794	7 (0.18)	3558	3 (0.08)
LVEF	3563	238 (6.26)	3508	53 (1.49)
Glucose	3309	492 (12.94)	3074	487 (13.68)
White blood cell count	3795	6 (0.16)	3557	4 (0.11)
Neutrophil count	3797	4 (0.11)	3559	2 (0.06)
Lymphocyte count	3796	5 (0.13)	3559	2 (0.06)
Monocyte count	3796	5 (0.13)	3558	3 (0.08)
Platelet count	3793	8 (0.21)	3555	6 (0.17)
NLR	3796	5 (0.13)	3559	2 (0.06)
MLR	3795	6 (0.16)	3558	3 (0.08)
SII	3792	9 (0.24)	3555	6 (0.17)
LDL cholesterol	3044	757 (19.92)	2926	635 (17.83)
HDL cholesterol	3139	662 (17.42)	3019	542 (15.22)
Triglycerides	3127	674 (17.73)	3007	554 (15.56)
Total cholesterol	3136	665 (17.50)	3016	545 (15.30)
C-reactive protein	2251	1550 (40.78)	2037	1524 (42.80)
Urea	3791	10 (0.26)	3553	8 (0.22)
Sodium	1504	2297 (60.43)	1504	2057 (57.76)
Potassium	1476	2325 (61.17)	1476	2085 (58.55)
Aspartate aminotransferase	3458	343 (9.02)	3249	312 (8.76)
Alanine aminotransferase	3602	199 (5.24)	3366	195 (5.48)
Mean platelet volume	3762	39 (1.03)	3525	36 (1.01)

Footnote: Available values and variable-level missingness are reported before multiple imputation. The survival-analysis cohort comprised patients with ascertainable mortality status and valid positive follow-up duration. Percentages were calculated using the corresponding cohort denominator.
Abbreviations: ACS, acute coronary syndrome; CKD-EPI, Chronic Kidney Disease Epidemiology Collaboration; eGFR, estimated glomerular filtration rate; HDL, high-density lipoprotein; LDL, low-density lipoprotein; LVEF, left ventricular ejection fraction; MLR, monocyte-to-lymphocyte ratio; NLR, neutrophil-to-lymphocyte ratio; SII, systemic immune-inflammation index; WHO, World Health Organization.

Supplementary Table S2. Comparison of baseline characteristics between patients included in and excluded from the survival analysis

Characteristic	Included (n = 3561)	Excluded (n = 240)	Standardized mean difference	p-value
Age, years	56.80 (13.00)	61.30 (14.81)	0.323	<0.001
Female sex, n (%)	846 (23.8)	72 (30.0)	0.141	0.035
ACS subtype, n (%)			0.268	<0.001
STEMI	2017 (56.6)	109 (45.4)		
NSTEMI	1047 (29.4)	77 (32.1)		
Unstable angina	496 (13.9)	53 (22.1)		
Missing	1 (0.0)	1 (0.4)		
Diabetes mellitus, yes, n (%)	628 (17.6)	66 (27.5)	0.318	<0.001
Missing	3 (0.1)	5 (2.1)		
Hypertension, yes, n (%)	937 (26.3)	113 (47.1)	0.503	<0.001
Missing	3 (0.1)	5 (2.1)		
Atrial fibrillation, yes, n (%)	91 (2.6)	11 (4.6)	0.205	<0.001
Missing	3 (0.1)	4 (1.7)		
Current smoking, yes, n (%)	1089 (30.6)	88 (36.7)	0.265	<0.001
Missing	8 (0.2)	7 (2.9)		
Prior coronary artery disease, yes, n (%)	913 (25.6)	89 (37.1)	0.296	<0.001
Missing	3 (0.1)	3 (1.2)		
WHO-defined anemia, yes, n (%)	1109 (31.1)	79 (32.9)	0.178	<0.001
Missing	3 (0.1)	4 (1.7)		
Hemoglobin, g/dL	13.46 (2.06)	13.15 (2.18)	0.145	0.026
Creatinine, mg/dL	0.86 [0.76–1.07]	1.07 [0.83–1.50]	0.333	<0.001
eGFR, mL/min/1.73 m²	95.70 [76.00–106.30]	71.00 [47.75–96.25]	0.646	<0.001
Glucose, mg/dL	122.50 [100.00–164.75]	144.00 [110.00–233.00]	0.402	<0.001
NLR	2.86 [1.88–4.61]	2.68 [1.65–5.65]	0.256	0.905
SII	700.65 [446.45–1193.45]	690.83 [441.78–1348.50]	0.256	0.513
LVEF, %	49.38 (10.06)	49.63 (11.60)	0.023	0.855

Footnote: Values are presented as mean (standard deviation), median [interquartile range], or number (%), as appropriate. Percentages were calculated using the full corresponding cohort denominator, and missing categories are shown explicitly. Student's t-test was used for normally distributed continuous variables, the Mann–Whitney U test for non-normally distributed continuous variables, and Pearson's chi-square test for categorical variables. P-values are descriptive and were not adjusted for multiple comparisons. Standardized mean differences quantify the magnitude of between-group imbalance; an absolute value ≥0.10 is commonly considered evidence of potentially relevant imbalance. Prior coronary artery disease was defined as any recorded nonzero coronary artery disease history category. **Abbreviations**: ACS, acute coronary syndrome; eGFR, estimated glomerular filtration rate; LVEF, left ventricular ejection fraction; NLR, neutrophil-to-lymphocyte ratio; NSTEMI, non-ST-segment elevation myocardial infarction; SII, systemic immune-inflammation index; STEMI, ST-segment elevation myocardial infarction; WHO, World Health Organization.

Supplementary Table S3. Baseline characteristics according to AAR-3 burden categories

Variable	Overall (n = 3557)	AAR-3 = 0 (n = 2153)	AAR-3 = 1 (n = 944)	AAR-3 = 2 (n = 342)	AAR-3 = 3 (n = 118)	p-value
Age, years	56.82 (12.99)	51.73 (10.07)	60.29 (11.82)	71.21 (11.74)	80.13 (3.48)	<0.001
Female sex, n (%)	846 (23.8)	322 (15.0)	318 (33.7)	138 (40.4)	68 (57.6)	<0.001
ACS subtype, n (%)						<0.001
NSTEMI	1045 (29.4)	568 (26.4)	281 (29.8)	151 (44.2)	45 (38.1)	
STEMI	2017 (56.7)	1271 (59.1)	536 (56.8)	149 (43.6)	61 (51.7)	
Unstable angina	494 (13.9)	313 (14.5)	127 (13.5)	42 (12.3)	12 (10.2)	
Diabetes mellitus, n (%)	626 (17.6)	324 (15.1)	174 (18.5)	98 (28.7)	30 (25.4)	<0.001
Hypertension, n (%)	935 (26.3)	507 (23.6)	245 (26.0)	141 (41.2)	42 (35.6)	<0.001
Atrial fibrillation, n (%)	90 (2.5)	29 (1.3)	25 (2.7)	28 (8.2)	8 (6.8)	<0.001
Current smoking, n (%)	1089 (30.7)	862 (40.1)	174 (18.5)	44 (12.9)	9 (7.7)	<0.001
Prior coronary artery disease, n (%)	910 (25.6)	477 (22.2)	271 (28.7)	123 (36.0)	39 (33.1)	<0.001
Hemoglobin, g/dL	13.70 [12.20–14.90]	14.50 [13.70–15.40]	12.00 [11.00–12.90]	11.20 [10.20–12.38]	11.00 [9.80–11.57]	<0.001
Creatinine, mg/dL	0.86 [0.76–1.07]	0.84 [0.76–0.97]	0.84 [0.73–1.06]	1.37 [0.93–1.82]	1.48 [1.19–1.78]	<0.001
eGFR, mL/min/1.73 m²	95.70 [76.00–106.30]	101.10 [89.40–108.70]	91.40 [71.50–103.30]	51.30 [36.50–70.32]	43.05 [31.40–51.72]	<0.001
Glucose, mg/dL	123.00 [100.00–165.00]	121.00 [99.00–160.00]	122.00 [99.50–166.50]	134.00 [103.00–176.00]	134.00 [108.00–188.00]	0.001
NLR	2.86 [1.88–4.61]	2.52 [1.70–3.98]	3.23 [2.18–5.08]	4.17 [2.53–6.60]	3.96 [2.85–6.38]	<0.001
SII	700.65 [446.33–1194.20]	621.06 [414.05–1019.43]	809.17 [509.65–1311.08]	962.78 [558.46–1535.77]	1097.48 [646.90–1534.00]	<0.001
LVEF, %	50.00 [42.50–60.00]	50.00 [45.00–60.00]	50.00 [42.00–60.00]	50.00 [40.00–58.00]	45.00 [37.00–52.00]	<0.001
All-cause death, n (%)	406 (11.4)	104 (4.8)	141 (14.9)	111 (32.5)	50 (42.4)	<0.001
Follow-up duration, years	3.35 [1.79–5.26]	3.81 [2.09–5.66]	2.94 [1.63–4.67]	2.32 [0.98–4.36]	1.88 [0.36–4.03]	<0.001

Footnote: Values are presented as mean (standard deviation), median [interquartile range], or number (%), as appropriate. P-values were calculated using one-way analysis of variance or the Kruskal–Wallis test for continuous variables and Pearson's chi-square test for categorical variables. AAR-3 was calculated as the sum of age ≥75 years, WHO-defined anemia, and eGFR <60 mL/min/1.73 m², with each component contributing 1 point. Because age, anemia/hemoglobin, and eGFR directly define the AAR-3 categories, differences and p-values for these variables are structurally expected and should be interpreted descriptively rather than as independent inferential findings. Prior coronary artery disease

was defined as any recorded nonzero coronary artery disease history category. ACS subtype was missing in one patient; percentages were calculated using available data. **Abbreviations**: AAR-3, Age–Anemia–Renal dysfunction; ACS, acute coronary syndrome; eGFR, estimated glomerular filtration rate; LVEF, left ventricular ejection fraction; NLR, neutrophil-to-lymphocyte ratio; NSTEMI, non-ST-segment elevation myocardial infarction; SII, systemic immune-inflammation index; STEMI, ST-segment elevation myocardial infarction; WHO, World Health Organization.

Supplementary Table S4. Outcome-independent Gower–PAM cluster-number selection, cluster sizes, and bootstrap stability

Panel A. Comparison of candidate cluster solutions

Candidate k	Average silhouette width	Cluster sizes, n (%)	Minimum cluster size, %	Minimum bootstrap Jaccard similarity	Mean bootstrap Jaccard similarity	Eligible	Selected
**2**	0.402	2431 (69.6); 1060 (30.4)	30.4	1.000	1.000	Yes	No
**3**	0.417	1969 (56.4); 919 (26.3); 603 (17.3)	17.3	0.893	0.952	Yes	Yes
**4**	0.412	1925 (55.1); 391 (11.2); 677 (19.4); 498 (14.3)	11.2	0.703	0.851	No	No

Panel B. Cluster size and stability of the selected three-phenotype solution

Phenotype	n	Percentage of phenomapping cohort	Bootstrap Jaccard similarity	Cluster dissolutions, n/100
A	1969	56.4	0.964	0/100
B	919	26.3	1.000	0/100
C	603	17.3	0.893	0/100

Footnote: Candidate solutions with k = 2, 3, and 4 were evaluated in the 3491-patient phenomapping cohort. Each candidate solution was assessed using 100 bootstrap resamples. A candidate solution was considered eligible if every cluster contained at least 10% of the phenomapping cohort and every cluster had a bootstrap Jaccard similarity of at least 0.75. Among eligible solutions, the number of clusters was selected by maximizing the average silhouette width, with the smaller value of k preferred only in the event of an exact tie. The k = 4 solution satisfied the cluster-size criterion but failed the prespecified stability criterion because one cluster had a bootstrap Jaccard similarity of 0.703. Mortality status and follow-up duration were not used to select the number of clusters, construct the clusters, or assign phenotype labels. After selection, neutral labels A, B, and C were assigned in descending order of cluster size. Bootstrap Jaccard similarities assess stability within the specified Gower–PAM procedure and do not establish agreement with alternative clustering algorithms or reproducibility in external cohorts. **Abbreviations**: PAM, partitioning around medoids.

Supplementary Table S5. Characteristics and post-clustering clinical outcomes of the selected Gower–PAM phenotypes

Panel A. Baseline characteristics used in the clustering analysis

Characteristic	Phenotype A (n = 1969; 56.4%)	Phenotype B (n = 919; 26.3%)	Phenotype C (n = 603; 17.3%)
Age, years	58.01 (12.68)	49.79 (10.70)	64.21 (11.93)
Hemoglobin, g/dL	13.14 (1.98)	14.48 (1.82)	12.89 (2.11)
eGFR, mL/min/1.73 m²	98.50 [83.80–107.20]	97.80 [82.30–107.70]	72.90 [48.00–91.45]
LVEF, %	48.91 (9.91)	50.55 (9.62)	49.15 (11.06)
NLR	3.03 [2.07–4.68]	2.36 [1.49–4.02]	3.13 [1.98–5.20]
SII	723.49 [472.98–1188.28]	586.70 [382.22–1072.13]	807.31 [467.44–1407.22]
Diabetes mellitus, n (%)	89 (4.5)	74 (8.1)	451 (74.8)
Hypertension, n (%)	124 (6.3)	242 (26.3)	545 (90.4)
Atrial fibrillation, n (%)	44 (2.2)	10 (1.1)	35 (5.8)
Current smoking, n (%)	0 (0.0)	919 (100.0)	141 (23.4)

Panel B. Outcomes examined after completion of clustering and phenotype labeling

Outcome	Phenotype A	Phenotype B	Phenotype C
All-cause deaths, n (%)	239 (12.1)	39 (4.2)	116 (19.2)
Follow-up duration, years	2.92 [1.69–4.64]	4.17 [2.14–6.29]	3.90 [1.40–6.41]

Footnote: Values are presented as mean (standard deviation), median [interquartile range], or number (%), as appropriate. The phenotypes were derived exclusively from six continuous variables—age, hemoglobin, eGFR, LVEF, log-transformed NLR, and log-transformed SII—and four binary categorical variables—diabetes mellitus, hypertension, atrial fibrillation, and current smoking. The four binary variables were entered as two-level nominal factors in the Gower calculation, without asymmetric-binary coding, and all ten clustering variables received equal unit weight. NLR and SII are presented on their original scales in this table. Because the phenotypes were constructed directly from the variables shown in Panel A, no inferential p-values are reported for between-phenotype comparisons of these characteristics. Mortality status and follow-up duration were not used for cluster construction, cluster-number selection, or phenotype labeling and were examined only after the clustering procedure had been completed. Phenotype labels were assigned neutrally in descending order of cluster size and do not represent ordered risk categories. The complete separation of current smoking in phenotype B should not be interpreted as evidence of a protective effect of smoking because phenotype membership simultaneously represents multiple correlated characteristics.
Abbreviations: eGFR, estimated glomerular filtration rate; LVEF, left ventricular ejection fraction; NLR, neutrophil-to-lymphocyte ratio; PAM, partitioning around medoids; SII, systemic immune-inflammation index.

Supplementary Table S6. Survival associations, proportional hazards assessments, and sensitivity analyses for the Gower–PAM phenotypes

Panel A. Global tests of association between the Gower–PAM phenotypes and mortality

Analysis	n	Deaths	χ^2	df	p-value
Overall follow-up, unadjusted log-rank test	3491	394	85.00	2	<0.001
First year, unadjusted log-rank test	3491	161	37.65	2	<0.001
Primary age-inclusive model, likelihood-ratio test for phenotype terms	3488	394	23.83	2	<0.001
Expanded age-inclusive clinical model, likelihood-ratio test for phenotype terms	3488	394	0.79	2	0.673

Panel B. Cox proportional hazards models for the Gower–PAM phenotypes

Model	Follow-up interval	n	Deaths	Comparison	HR (95% CI)	p-value	C-index
Unadjusted	Overall follow-up	3491	394	Phenotype B vs. A	0.28 (0.20–0.39)	<0.001	0.622
Unadjusted	Overall follow-up	3491	394	Phenotype C vs. A	1.35 (1.08–1.68)	0.009	0.622
Unadjusted	0–365 days	3491	161	Phenotype B vs. A	0.19 (0.10–0.37)	<0.001	0.620
Unadjusted	0–365 days	3491	161	Phenotype C vs. A	1.28 (0.90–1.83)	0.168	0.620
Unadjusted	>365-day landmark	2976	233	Phenotype B vs. A	0.34 (0.22–0.50)	<0.001	0.625
Unadjusted	>365-day landmark	2976	233	Phenotype C vs. A	1.40 (1.05–1.87)	0.023	0.625
Primary age-inclusive model	Overall follow-up	3488	394	Phenotype B vs. A	0.45 (0.32–0.64)	<0.001	0.766
Primary age-inclusive model	Overall follow-up	3488	394	Phenotype C vs. A	0.92 (0.73–1.16)	0.494	0.766
Primary age-inclusive model	0–365 days	3488	161	Phenotype B vs. A	0.34 (0.18–0.66)	0.001	0.763
Primary age-inclusive model	0–365 days	3488	161	Phenotype C vs. A	0.96 (0.66–1.40)	0.846	0.763
Primary age-inclusive model	>365-day landmark	2973	233	Phenotype B vs. A	0.52 (0.34–0.79)	0.002	0.775

Primary age-inclusive model	>365-day landmark	2973	233	Phenotype C vs. A	0.90 (0.67–1.22)	0.493	0.775
Expanded age-inclusive clinical model	Overall follow-up	3488	394	Phenotype B vs. A	0.97 (0.47–1.98)	0.922	0.770
Expanded age-inclusive clinical model	Overall follow-up	3488	394	Phenotype C vs. A	1.21 (0.78–1.88)	0.385	0.770
Expanded age-inclusive clinical model	0–365 days	3488	161	Phenotype B vs. A	0.67 (0.20–2.28)	0.519	0.767
Expanded age-inclusive clinical model	0–365 days	3488	161	Phenotype C vs. A	1.05 (0.50–2.22)	0.889	0.767
Expanded age-inclusive clinical model	>365-day landmark	2973	233	Phenotype B vs. A	1.14 (0.47–2.78)	0.771	0.781
Expanded age-inclusive clinical model	>365-day landmark	2973	233	Phenotype C vs. A	1.32 (0.77–2.27)	0.312	0.781

Panel C. Proportional hazards assessments based on scaled Schoenfeld residuals

Model	Follow-up interval	Phenotype χ^2 (df)	Phenotype p-value	Global χ^2 (df)	Global p-value
Unadjusted	Overall follow-up	8.16 (2)	0.017	8.16 (2)	0.017
Unadjusted	0–365 days	1.19 (2)	0.553	1.19 (2)	0.553
Unadjusted	>365-day landmark	8.40 (2)	0.015	8.40 (2)	0.015
Primary age-inclusive model	Overall follow-up	4.35 (2)	0.114	18.23 (7)	0.011
Primary age-inclusive model	0–365 days	1.23 (2)	0.541	10.58 (7)	0.158
Primary age-inclusive model	>365-day landmark	5.51 (2)	0.064	12.25 (7)	0.093
Expanded age-inclusive clinical model	Overall follow-up	4.09 (2)	0.130	22.54 (11)	0.021
Expanded age-inclusive clinical model	0–365 days	1.23 (2)	0.542	13.57 (11)	0.258
Expanded age-inclusive clinical model	>365-day landmark	5.23 (2)	0.073	20.36 (11)	0.041

Panel D1. Internal separation obtained with alternative clustering methods

Clustering method	n	Selected k	Distance measure	Average silhouette width
Gower–PAM mixed-data clustering	3491	3	Gower	0.417
Standardized mixed-variable k-means	3491	3	Euclidean	0.192
Standardized continuous-only k-means	3491	3	Euclidean	0.219

Panel D2. Agreement between clustering methods

Comparison	n	Adjusted Rand index
Gower–PAM vs. mixed-variable k-means	3491	0.049
Gower–PAM vs. continuous-only k-means	3491	0.026
Mixed-variable k-means vs. continuous-only k-means	3491	0.495

Abbreviations: ACS, acute coronary syndrome; CI, confidence interval; HR, hazard ratio; PAM, partitioning around medoids.

Phenotype A was used as the reference group. The primary age-inclusive model was adjusted for age, sex, ACS subtype, and history of coronary artery disease. The expanded age-inclusive clinical model additionally included diabetes mellitus, hypertension, atrial fibrillation, and smoking. Because these additional clinical characteristics were also used to construct the phenotypes, the expanded model represents a deliberately conservative, potentially overadjusted sensitivity analysis.

The first-year analysis censored follow-up at 365 days. The >365-day landmark analysis included patients who were alive and under observation at 365 days and evaluated subsequent mortality. Proportional hazards assumptions were assessed using scaled Schoenfeld residuals.

In the primary age-inclusive overall model, nominal covariate-level proportional hazards departures were observed for ACS subtype (p = 0.048) and coronary artery disease history (p = 0.046); the phenotype term itself did not violate the assumption (p = 0.114). The time-stratified models substantially reduced these departures. In the expanded model, nominal departures were observed for ACS subtype, coronary artery disease history, and smoking during overall follow-up; these findings support emphasizing the prespecified first-year and >365-day estimates.

The number of clusters was selected without using mortality outcomes. Average silhouette width quantified internal cluster separation. The adjusted Rand index quantified agreement between alternative clustering assignments; values close to zero indicate minimal patient-level agreement. Thus, the low agreement between Gower–PAM and k-means indicates method-dependent phenotype membership despite the stronger internal separation obtained with Gower–PAM.

Supplementary Table S7. Selected univariable Cox proportional hazards analyses for long-term all-cause mortality

Variable	Scale or comparison	Unadjusted HR (95% CI)	p-value
Age	Per 1-year increase	1.08 (1.07–1.08)	<0.001
Female sex	Female vs. male	1.63 (1.33–2.00)	<0.001
Prior coronary artery disease	Present vs. absent	1.76 (1.45–2.16)	<0.001
Diabetes mellitus	Present vs. absent	1.27 (1.01–1.61)	0.040
Hypertension	Present vs. absent	1.15 (0.93–1.42)	0.190
Atrial fibrillation	Present vs. absent	1.67 (1.04–2.68)	0.034
Current smoking	Current smoker vs. non-smoker	0.27 (0.20–0.36)	<0.001
Hemoglobin	Per 1-g/dL increase	0.76 (0.73–0.79)	<0.001
eGFR	Per 1-mL/min/1.73 m² increase	0.97 (0.97–0.97)	<0.001
LVEF	Per 1-percentage-point increase	0.95 (0.94–0.95)	<0.001
NLR	Per 1-unit increase	1.04 (1.03–1.05)	<0.001
SII	Per 500-unit increase	1.10 (1.08–1.12)	<0.001
AAR-3 burden	Per 1-point increase	2.49 (2.28–2.73)	<0.001
CARE-4 burden	Per 1-point increase	2.43 (2.25–2.63)	<0.001

Footnote: Hazard ratios were estimated using univariable Cox proportional hazards regression with Efron handling of tied event times and were based on the available observations for each variable. The AAR-3 analysis included 3557 patients and 406 deaths, whereas the CARE-4 analysis included 3505 patients and 397 deaths. The SII estimate is presented per 500-unit increase. Univariable associations, particularly that observed for current smoking, should not be interpreted causally.

Abbreviations: AAR-3, Age–Anemia–Renal dysfunction; CARE-4, Cardiac dysfunction–Age–Renal dysfunction–Anemia; CI, confidence interval; eGFR, estimated glomerular filtration rate; HR, hazard ratio; LVEF, left ventricular ejection fraction; NLR, neutrophil-to-lymphocyte ratio; SII, systemic immune-inflammation index.

Supplementary Table S8. Multiple-imputation input missingness, pooled score effects, model performance, incremental tests, and convergence diagnostics

Panel A. Variables imputed and variable-specific imputation methods

Variable	Missing, n (%)	Imputation method
Age	1 (0.03)	Predictive mean matching
ACS subtype	1 (0.03)	Polytomous logistic regression
Diabetes mellitus	3 (0.08)	Binary logistic regression
Hypertension	3 (0.08)	Binary logistic regression
Atrial fibrillation	3 (0.08)	Binary logistic regression
Current smoking	8 (0.22)	Binary logistic regression
Prior coronary artery disease	3 (0.08)	Binary logistic regression
Creatinine	1 (0.03)	Predictive mean matching
eGFR	2 (0.06)	Predictive mean matching
Hemoglobin	3 (0.08)	Predictive mean matching
LVEF	53 (1.49)	Predictive mean matching

Panel B. Complete-case and Rubin-pooled framework associations

Framework	Analysis	n	Deaths	Imputations	HR (95% CI)	p-value	Fraction of missing information
AAR-3, per 1-point increase	Common complete-case	3492	394	–	1.65 (1.45–1.87)	<0.001	–
AAR-3, per 1-point increase	Multiple imputation, Rubin-pooled	3561	407	20	1.65 (1.45–1.88)	<0.001	0.7%
CARE-4, per 1-point increase	Common complete-case	3492	394	–	1.81 (1.63–2.01)	<0.001	–
CARE-4, per 1-point increase	Multiple imputation, Rubin-pooled	3561	407	20	1.81 (1.63–2.01)	<0.001	1.2%
ACEF, per 1-unit increase	Common complete-case	3492	394	–	2.48 (2.14–2.87)	<0.001	–
ACEF, per 1-unit increase	Multiple imputation, Rubin-pooled	3561	407	20	2.42 (2.09–2.81)	<0.001	6.1%

Panel C. Model discrimination in the complete-case and imputed datasets

Model	Complete-case C-index (SE)	Mean C-index across 20 imputed datasets (between-imputation SD)	Range across imputed datasets
Model B: age-inclusive clinical reference	0.7697 (0.0115)	0.7708 (0.0004)	0.7702–0.7713
Model B + AAR-3	0.7927 (0.0108)	0.7938 (0.0003)	0.7933–0.7942
Model B + CARE-4	0.8145 (0.0100)	0.8155 (0.0004)	0.8147–0.8162
Model B + ACEF	0.8122 (0.0104)	0.8121 (0.0006)	0.8112–0.8134

Panel D. Incremental model tests

Framework added to Model B	Complete-case likelihood-ratio χ^2 (df = 1)	Complete-case p-value	Multiple-imputation D1 pooled Wald F (df1, df2)	Pooled p-value
AAR-3	56.77	<0.001	60.22 (1, 394.95)	<0.001
CARE-4	113.35	<0.001	121.93 (1, 394.29)	<0.001
ACEF	116.45	<0.001	138.86 (1, 357.86)	<0.001

Panel E. Multiple-imputation run and convergence summary

Diagnostic	**Result**
Analysis cohort	3561 patients; 407 deaths
Number of imputed datasets	20
Iterations per imputation	20
Variables imputed	11
Initially missing covariate cells imputed	81
Random seed	20260804
Logged imputation events or warnings	None
Maximum absolute autocorrelation at the final iteration	0.375
Trace-plot assessment	No sustained directional drift was evident after the initial iterations; discrete traces for several categorical variables reflected their very small numbers of missing observations
Overall interpretation	Convergence diagnostics and the close agreement between complete-case and imputed estimates supported use of multiple imputation as a sensitivity analysis

Footnote: Multiple imputation by chained equations was performed in the complete survival-analysis cohort. Twenty imputed datasets were generated using 20 iterations and random seed 20260804. Predictive mean matching was used for continuous variables, binary logistic regression for dichotomous variables, and polytomous logistic regression for ACS subtype.
Death status and the Nelson–Aalen cumulative hazard estimate were included as predictors in the imputation models; neither the survival outcomes nor the Nelson–Aalen estimate was imputed. Raw

follow-up time was not used as an imputation predictor. AAR-3, CARE-4, and ACEF were recalculated after completion of each imputed dataset rather than imputed directly.
The reference model was the age-inclusive Model B and contained age, sex, ACS subtype, diabetes mellitus, hypertension, atrial fibrillation, current smoking, and prior coronary artery disease. Each framework was added separately. Regression coefficients and confidence intervals were pooled using Rubin’s rules. Incremental effects after multiple imputation were evaluated using the D1 pooled multivariate Wald test.
Harrell’s C-indices in the multiple-imputation analysis are presented as the mean across the 20 completed datasets with the between-imputation standard deviation and observed range; these C-indices were not Rubin-pooled. Trace plots are presented in Supplementary Figure S1. Multiple imputation was used as a sensitivity analysis and did not replace the primary common complete-case analysis.
Abbreviations: AAR-3, Age–Anemia–Renal dysfunction; ACEF, Age, Creatinine, and Ejection Fraction; ACS, acute coronary syndrome; CARE-4, Cardiac dysfunction–Age–Renal dysfunction–Anemia; CI, confidence interval; eGFR, estimated glomerular filtration rate; HR, hazard ratio; LVEF, left ventricular ejection fraction; SD, standard deviation; SE, standard error.

Supplementary Table S9. Full common-cohort hierarchical comparison of AAR-3, CARE-4, and ACEF, including effect estimates, discrimination, model fit, and incremental tests

Model family	Framework added	Adjusted HR (95% CI)	p-value	Harrell's C-index (95% CI)	Δ C-index	AIC	BIC	Likelihood-ratio χ² (p-value)
Model A: clinical without age	Reference	–	–	0.685 (0.660–0.710)	0.0000	5906.2	5938.0	–
Model A: clinical without age	AAR-3	2.28 (2.06–2.52)	<0.001	0.772 (0.748–0.795)	0.0868	5682.8	5718.6	225.42 (<0.001)
Model A: clinical without age	CARE-4	2.26 (2.07–2.46)	<0.001	0.801 (0.779–0.822)	0.1158	5612.0	5647.8	296.20 (<0.001)
Model A: clinical without age	ACEF	3.33 (2.95–3.75)	<0.001	0.809 (0.789–0.830)	0.1244	5624.8	5660.6	283.41 (<0.001)
Model B: age-inclusive clinical reference	Reference	–	–	0.770 (0.747–0.792)	0.0000	5680.2	5715.9	–
Model B: age-inclusive clinical reference	AAR-3	1.65 (1.45–1.87)	<0.001	0.793 (0.772–0.814)	0.0231 (0.0119–0.0354)	5625.4	5665.2	56.77 (<0.001)
Model B: age-inclusive clinical reference	CARE-4	1.81 (1.63–2.01)	<0.001	0.815 (0.795–0.834)	0.0449 (0.0292–0.0603)	5568.8	5608.6	113.35 (<0.001)
Model B: age-inclusive clinical reference	ACEF	2.48 (2.14–2.87)	<0.001	0.812 (0.792–0.833)	0.0425 (0.0284–0.0567)	5565.7	5605.5	116.45 (<0.001)
Model C: age plus continuous biological components	Reference	–	–	0.828 (0.808–0.847)	0.0000	5500.6	5548.3	–
Model C: age plus continuous biological components	AAR-3	1.05 (0.87–1.26)	0.626	0.828 (0.809–0.847)	0.0003	5502.4	5554.0	0.24 (0.626)

Model C: age plus continuous biological components	CARE-4	1.12 (0.96–1.32)	0.155	0.829 (0.810–0.848)	0.0013	5500.6	5552.3	2.02 (0.156)
Model C: age plus continuous biological components	ACEF	0.90 (0.64–1.26)	0.551	0.827 (0.808–0.847)	−0.0001	5502.2	5553.9	0.36 (0.550)

Footnote: All models were fitted in the same common complete-case cohort of 3492 patients, in whom 394 deaths occurred. Each framework was added separately to its corresponding reference model; AAR-3, CARE-4, and ACEF were not entered simultaneously.

Model A included sex, ACS subtype, diabetes mellitus, hypertension, atrial fibrillation, current smoking, and prior coronary artery disease but omitted chronological age. It represents a historical sensitivity analysis retained for comparison with the originally submitted models and should not be interpreted as the primary analysis. Model B additionally included chronological age and was designated as the primary age-inclusive clinical reference model. Model C included all Model B covariates together with continuous hemoglobin, eGFR, and LVEF and evaluated whether the simplified frameworks contributed information beyond their underlying continuous biological components. Hazard ratios for AAR-3 and CARE-4 are reported per 1-point increase. The ACEF hazard ratio is reported per 1-unit increase. Harrell's C-index is presented with its model-based 95% confidence interval. Δ C-index was calculated relative to the corresponding reference model within each model family. For the primary Model B comparisons, 95% confidence intervals for Δ C-index were obtained from 2000 paired patient-level nonparametric bootstrap resamples; Model A and Model C Δ C-index values are presented as point estimates only. The paired-bootstrap difference between the CARE-4- and ACEF-extended Model B models was 0.0024 (95% CI, −0.0109 to 0.0150). Because this interval included zero, their discrimination estimates were considered numerically close, but the analysis does not establish statistical equivalence. Likelihood-ratio tests compared each framework-extended model with its corresponding reference model and had 1 degree of freedom. Lower AIC and BIC values indicate better model fit.

In the primary Model B comparisons, CARE-4 and ACEF yielded numerically close C-index estimates, and the paired-bootstrap confidence interval for their difference included zero. Both showed larger increases in C-index than AAR-3. CARE-4 had a slightly higher point estimate for the C-index, whereas ACEF had slightly lower AIC and BIC values; these findings do not establish superiority or statistical equivalence of either framework. None of the three frameworks materially improved discrimination or model fit after age, hemoglobin, eGFR, and LVEF were already included continuously in Model C. Because the global proportional hazards assumption was not satisfied throughout complete follow-up in the primary Model B analyses, the full-follow-up hazard ratios should be interpreted as average associations over time. The principal first-year and >365-day landmark estimates are reported in Supplementary Table S11.

Abbreviations: AAR-3, Age–Anemia–Renal dysfunction; ACEF, Age, Creatinine, and Ejection Fraction; ACS, acute coronary syndrome; AIC, Akaike information criterion; BIC, Bayesian information criterion; CARE-4, Cardiac dysfunction–Age–Renal dysfunction–Anemia; CI, confidence interval; eGFR, estimated glomerular filtration rate; HR, hazard ratio; LVEF, left ventricular ejection fraction; SE, standard error.

Supplementary Table S10. Variable-level proportional hazards assessments for the primary full-follow-up common-cohort models

Variable or test	Model B reference, χ^2 (df); p-value	Model B + AAR-3, χ^2 (df); p-value	Model B + CARE-4, χ^2 (df); p-value	Model B + ACEF, χ^2 (df); p-value
Age	2.29 (1); 0.130	2.05 (1); 0.152	2.01 (1); 0.157	3.38 (1); 0.066
Female sex	1.82 (1); 0.178	1.98 (1); 0.159	2.50 (1); 0.114	2.16 (1); 0.142
ACS subtype	6.59 (2); 0.037	6.31 (2); 0.043	7.00 (2); 0.030	7.53 (2); 0.023
Diabetes mellitus	0.37 (1); 0.541	0.34 (1); 0.557	0.16 (1); 0.687	<0.01 (1); 0.952
Hypertension	1.30 (1); 0.254	1.25 (1); 0.264	1.86 (1); 0.172	3.20 (1); 0.074
Atrial fibrillation	1.22 (1); 0.270	1.25 (1); 0.264	1.25 (1); 0.264	1.66 (1); 0.198
Current smoking	5.24 (1); 0.022	5.80 (1); 0.016	6.21 (1); 0.013	5.07 (1); 0.024
Prior coronary artery disease	4.15 (1); 0.042	4.18 (1); 0.041	5.82 (1); 0.016	7.72 (1); 0.005
Added framework term	–	AAR-3: 0.01 (1); 0.940	CARE-4: 1.20 (1); 0.273	ACEF: 0.01 (1); 0.917
**Global test**	**21.96 (9); 0.009**	**23.86 (10); 0.008**	**29.60 (10); 0.001**	**28.57 (10); 0.001**

Footnote: Proportional hazards assumptions were evaluated using tests based on scaled Schoenfeld residuals. All models were fitted in the common complete-case cohort of 3492 patients, in whom 394 deaths occurred.
Model B included age, sex, ACS subtype, diabetes mellitus, hypertension, atrial fibrillation, current smoking, and prior coronary artery disease. AAR-3, CARE-4, and ACEF were added separately to the Model B reference model.
No evidence of non-proportionality was observed for the AAR-3, CARE-4, or ACEF terms themselves. However, the global tests were significant in all full-follow-up Model B analyses. The nominal variable-level departures were primarily associated with ACS subtype, current smoking, and prior coronary artery disease rather than with the vulnerability frameworks.
The p-values in this table are diagnostic and were not adjusted for multiple testing. Consequently, individual values below 0.05 should be interpreted as nominal evidence of time-varying effects rather than as independent confirmatory hypothesis tests. Because the global assumptions were not satisfied throughout complete follow-up, full-follow-up hazard ratios were interpreted as average associations over time, and the first-year and >365-day landmark estimates were designated as the principal effect estimates.
Abbreviations: AAR-3, Age–Anemia–Renal dysfunction; ACEF, Age, Creatinine, and Ejection Fraction; ACS, acute coronary syndrome; CARE-4, Cardiac dysfunction–Age–Renal dysfunction–Anemia.

Supplementary Table S11. First-year and >365-day landmark associations, discrimination, and proportional hazards assessments

Panel A. Time-specific framework associations and model discrimination

Follow-up interval	Framework	n	Deaths	Adjusted HR (95% CI)	p-value	Reference-model C-index	Framework-extended C-index (SE)	Δ C-index
0–365 days	AAR-3	3492	161	1.78 (1.46–2.19)	<0.001	0.767	0.789 (0.016)	0.0220
0–365 days	CARE-4	3492	161	2.08 (1.76–2.46)	<0.001	0.767	0.818 (0.015)	0.0517
0–365 days	ACEF	3492	161	2.66 (2.14–3.30)	<0.001	0.767	0.820 (0.015)	0.0537
>365-day landmark	AAR-3	2977	233	1.56 (1.32–1.85)	<0.001	0.780	0.800 (0.014)	0.0197
>365-day landmark	CARE-4	2977	233	1.66 (1.44–1.91)	<0.001	0.780	0.813 (0.013)	0.0325
>365-day landmark	ACEF	2977	233	2.39 (1.95–2.92)	<0.001	0.780	0.809 (0.014)	0.0286

Panel B. Variable-level proportional hazards test p-values during the first 365 days

Variable or test	Model B reference	Model B + AAR-3	Model B + CARE-4	Model B + ACEF
Age	0.159	0.157	0.151	0.133
Female sex	0.794	0.823	0.804	0.709
ACS subtype	0.218	0.212	0.193	0.216
Diabetes mellitus	0.284	0.287	0.285	0.297
Hypertension	0.434	0.445	0.437	0.399
Atrial fibrillation	0.650	0.642	0.658	0.674
Current smoking	0.362	0.361	0.360	0.356
Prior coronary artery disease	0.034	0.034	0.032	0.031
Added framework term	–	0.924	0.887	0.805
**Global test**	**0.219**	**0.211**	**0.153**	**0.130**

Panel C. Variable-level proportional hazards test p-values in the >365-day landmark analysis

Variable or test	Model B reference	Model B + AAR-3	Model B + CARE-4	Model B + ACEF
Age	0.449	0.522	0.537	0.371
Female sex	0.046	0.033	0.025	0.041
ACS subtype	0.571	0.640	0.640	0.561
Diabetes mellitus	0.629	0.672	0.600	0.337
Hypertension	0.370	0.399	0.328	0.189
Atrial fibrillation	0.172	0.149	0.133	0.103
Current smoking	0.003	0.002	0.002	0.004
Prior coronary artery disease	0.761	0.786	0.632	0.406
Added framework term	–	0.937	0.669	0.671
**Global test**	**0.039**	**0.044**	**0.033**	**0.023**

Footnote: The first-year analyses included all 3492 patients in the common complete-case cohort and censored follow-up at 365 days. The >365-day landmark analyses included the 2977 patients who were alive and remained under observation at 365 days; 233 subsequent deaths occurred during landmark follow-up.

All models were adjusted using the age-inclusive Model B covariates: age, sex, ACS subtype, diabetes mellitus, hypertension, atrial fibrillation, current smoking, and prior coronary artery disease. AAR-3, CARE-4, and ACEF were added separately. Hazard ratios for AAR-3 and CARE-4 are reported per 1-point increase, and the ACEF hazard ratio is reported per 1-unit increase.

Proportional hazards assumptions were evaluated using tests based on scaled Schoenfeld residuals. No evidence of non-proportionality was observed for any of the three framework terms during either follow-up interval. During the first year, all global model tests were nonsignificant, although prior coronary artery disease showed a nominal variable-level departure. In the landmark period, residual global non-proportionality was primarily associated with sex and current smoking; the framework terms themselves remained nonsignificant.

The diagnostic p-values were not adjusted for multiple testing. The time-specific analyses should therefore be interpreted primarily as estimates of the framework associations within clinically defined follow-up intervals rather than as proof that every covariate satisfied proportional hazards throughout each interval.

Abbreviations: AAR-3, Age–Anemia–Renal dysfunction; ACEF, Age, Creatinine, and Ejection Fraction; ACS, acute coronary syndrome; CARE-4, Cardiac dysfunction–Age–Renal dysfunction–Anemia; CI, confidence interval; HR, hazard ratio; SE, standard error.

Supplementary Table S12. Specifications and results of the adjusted restricted cubic spline analyses

Panel A. Spline and figure specifications

Panel and exposure	Analysis cohort	Displayed range	Hazard-ratio reference	Knot locations
A. Age, years	n = 3492; 394 deaths	30.00–86.00	55.4 years	37.62, 50.00, 62.00, 79.30
B. Hemoglobin, g/dL	n = 3492; 394 deaths	8.10–17.51	13.7 g/dL	9.80, 12.90, 14.40, 16.50
C. eGFR, mL/min/1.73 m²	n = 3492; 394 deaths	10.76–123.34	95.7 mL/min/1.73 m²	37.40, 86.30, 102.42, 115.75
D. LVEF, %	n = 3492; 394 deaths	22.00–65.00	50.0%	30.00, 45.00, 55.00, 60.00

Panel B. Overall and nonlinear association tests

Exposure	Overall χ^2 (df)	Overall p-value	Nonlinearity χ^2 (df)	Nonlinearity p-value	Covariate adjustment
Age	207.21 (3)	<0.001	0.48 (2)	0.787	Sex, ACS subtype, diabetes mellitus, hypertension, atrial fibrillation, current smoking, and prior coronary artery disease
Hemoglobin	38.38 (3)	<0.001	13.65 (2)	0.001	Age, sex, ACS subtype, diabetes mellitus, hypertension, atrial fibrillation, current smoking, and prior coronary artery disease
eGFR	118.64 (3)	<0.001	7.96 (2)	0.019	Age, sex, ACS subtype, diabetes mellitus, hypertension, atrial fibrillation, current smoking, and prior coronary artery disease
LVEF	115.66 (3)	<0.001	3.41 (2)	0.182	Age, sex, ACS subtype, diabetes mellitus, hypertension, atrial fibrillation, current smoking, and prior coronary artery disease

Footnote: Restricted cubic spline models were fitted separately for age, hemoglobin, eGFR, and LVEF in the common complete-case cohort of 3492 patients, in whom 394 deaths occurred. Four knots were positioned at the 5th, 35th, 65th, and 95th percentiles of each exposure distribution. Hazard ratios were referenced to the exposure-specific median. Curves were displayed from the 1st to the 99th percentile to limit visual extrapolation in sparsely populated tails.

The age model was adjusted for sex, ACS subtype, diabetes mellitus, hypertension, atrial fibrillation, current smoking, and prior coronary artery disease. Models for hemoglobin, eGFR, and LVEF additionally included chronological age. The four continuous exposures were evaluated in separate spline models rather than included simultaneously as spline terms.

Solid curves in main-text Figure 4 represent adjusted hazard ratios, shaded regions represent 95% confidence intervals, vertical dashed lines indicate exposure-specific reference values, dotted vertical lines indicate knot locations, and rug plots show the observed distributions.
There was strong evidence of an overall association with mortality for all four exposures. The association was approximately linear for age and LVEF because their nonlinearity tests were nonsignificant. Evidence of nonlinearity was observed for hemoglobin and eGFR. These spline analyses characterize adjusted exposure–outcome shapes and should not be interpreted as establishing causal thresholds or optimal clinical cutoffs.
Abbreviations: ACS, acute coronary syndrome; eGFR, estimated glomerular filtration rate; LVEF, left ventricular ejection fraction.

Supplementary Table S13. Internal temporal-period robustness analyses and proportional hazards assessments in the common complete-case cohort

Panel A. Period-specific associations, discrimination, and incremental model tests

Calendar period	Framework	n	Deaths	Adjusted HR (95% CI)	p-value	Reference C-index	Extended C-index	Δ C-index	Likelihood-ratio χ^2 (p-value)
2017–2019	AAR-3	1535	208	1.61 (1.34–1.94)	<0.001	0.703	0.727	0.0241	25.87 (<0.001)
2017–2019	CARE-4	1535	208	1.80 (1.55–2.09)	<0.001	0.703	0.762	0.0585	57.83 (<0.001)
2017–2019	ACEF	1535	208	2.66 (2.16–3.27)	<0.001	0.703	0.767	0.0639	66.35 (<0.001)
2020–2024	AAR-3	1957	186	1.68 (1.39–2.02)	<0.001	0.795	0.816	0.0212	29.06 (<0.001)
2020–2024	CARE-4	1957	186	1.83 (1.56–2.15)	<0.001	0.795	0.830	0.0349	53.58 (<0.001)
2020–2024	ACEF	1957	186	2.30 (1.86–2.84)	<0.001	0.795	0.820	0.0252	48.75 (<0.001)

Panel B. Variable-level proportional hazards test p-values for the 2017–2019 period

Variable or test	Model + AAR-3	Model + CARE-4	Model + ACEF
Age	0.124	0.138	0.096
Female sex	0.285	0.276	0.266
ACS subtype	0.021	0.014	0.029
Diabetes mellitus	0.223	0.219	0.175
Hypertension	0.765	0.676	0.757
Atrial fibrillation	0.565	0.629	0.557
Prior coronary artery disease	0.081	0.069	0.097
Framework term	0.341	0.827	0.947
**Global test**	**0.129**	**0.069**	**0.118**

Panel C. Variable-level proportional hazards test p-values for the 2020–2024 period

Variable or test	Model + AAR-3	Model + CARE-4	Model + ACEF
Age	0.689	0.660	0.401
Female sex	0.037	0.022	0.024
ACS subtype	0.594	0.584	0.589
Diabetes mellitus	0.818	0.938	0.839
Hypertension	0.499	0.409	0.199
Atrial fibrillation	0.522	0.460	0.352
Prior coronary artery disease	0.423	0.227	0.127
Framework term	0.438	0.269	0.906
**Global test**	**0.388**	**0.188**	**0.190**

Footnote: This was an internal temporal robustness analysis within the same single-center cohort and should not be interpreted as temporal external validation. The common complete-case cohort was divided into patients admitted during 2017–2019 and 2020–2024. The 2020 cut-point was chosen pragmatically to create two chronologically distinct subcohorts of broadly comparable size with sufficient observations for stable estimation; it was not selected according to mortality rates, effect estimates, discrimination metrics, statistical significance, or other outcome-related model results. The earlier period included 1535 patients and 208 deaths, with a median follow-up of 2.51 years. The later period included 1957 patients and 186 deaths, with a median follow-up of 4.57 years.
Period-specific models were adjusted for age, sex, ACS subtype, diabetes mellitus, hypertension, atrial fibrillation, and prior coronary artery disease. Current smoking was omitted from both period-specific models because the 2017–2019 common cohort included only 25 current smokers and none experienced the outcome, resulting in complete separation. An identical covariate set was used in both periods to preserve comparability.
AAR-3 and CARE-4 hazard ratios are reported per 1-point increase, and the ACEF hazard ratio is reported per 1-unit increase. Likelihood-ratio tests compared each framework-extended model with its period-specific clinical reference model and had 1 degree of freedom.
Proportional hazards assumptions were assessed using scaled Schoenfeld residuals. No evidence of non-proportionality was observed for any framework term or global model in either calendar period. Nominal variable-level departures were observed for ACS subtype during 2017–2019 and for sex during 2020–2024. Diagnostic p-values were not adjusted for multiple testing.
The direction and magnitude of the framework associations were generally consistent across the two calendar periods. However, the periods differed in sample size, event frequency, follow-up duration, and case mix. These findings therefore support internal temporal consistency but do not establish external validity or transportability.
Abbreviations: AAR-3, Age–Anemia–Renal dysfunction; ACEF, Age, Creatinine, and Ejection Fraction; ACS, acute coronary syndrome; CARE-4, Cardiac dysfunction–Age–Renal dysfunction–Anemia; CI, confidence interval; HR, hazard ratio.

Supplementary Table S14. Cross-validated survival decision-curve results at selected threshold probabilities and distribution of out-of-fold predicted risks

Panel A. Net benefit at selected threshold probabilities

Threshold probability	Treat none	Treat all	Model B	Model B + AAR-3	Model B + CARE-4	Model B + ACEF	Model B + continuous Hb, eGFR, and LVEF
1%	0.0000	0.0378	0.0391	0.0393	0.0393	0.0393	0.0394
1.5%	0.0000	0.0329	0.0353	0.0352	0.0358	0.0361	0.0370
2%	0.0000	0.0280	0.0328	0.0324	0.0329	0.0341	0.0342
2.5%	0.0000	0.0230	0.0284	0.0299	0.0312	0.0315	0.0324
3%	0.0000	0.0180	0.0251	0.0266	0.0300	0.0284	0.0300
5%	0.0000	−0.0027	0.0180	0.0202	0.0227	0.0230	0.0246
10%	0.0000	−0.0584	0.0064	0.0092	0.0111	0.0121	0.0127
15%	0.0000	−0.1206	0.0011	0.0029	0.0063	0.0058	0.0087
20%	0.0000	−0.1907	0.0001	0.0025	0.0044	0.0015	0.0045

Panel B. Incremental net benefit compared with Model B

Threshold probability	Model B + AAR-3	Model B + CARE-4	Model B + ACEF	Model B + continuous Hb, eGFR, and LVEF
1%	0.0002	0.0002	0.0002	0.0004
1.5%	−0.0001	0.0005	0.0008	0.0017
2%	−0.0004	0.0001	0.0013	0.0013
2.5%	0.0015	0.0028	0.0031	0.0039
3%	0.0015	0.0048	0.0033	0.0049
5%	0.0023	0.0047	0.0050	0.0066
10%	0.0028	0.0047	0.0057	0.0063
15%	0.0018	0.0052	0.0047	0.0076
20%	0.0024	0.0044	0.0015	0.0045

Panel C. Distribution of out-of-fold predicted 1-year mortality risks (%)

Model	Minimum	25th percentile	Median	Mean	75th percentile	Maximum
Model B	0.10	1.25	2.94	4.79	6.52	37.22
Model B + AAR-3	0.18	1.28	2.71	4.79	5.52	46.26
Model B + CARE-4	0.20	1.16	2.30	4.76	5.22	64.38
Model B + ACEF	0.13	1.18	2.65	4.73	5.55	90.33
Model B + continuous Hb, eGFR, and LVEF	0.07	0.88	2.05	4.77	5.26	79.36

Footnote: Decision-curve analysis was performed at a 365-day prediction horizon in the common complete-case cohort (n = 3492) using a censoring-aware survival approach. By 365 days, 161 deaths had occurred, 2980 patients were known to be event-free, and 351 patients had been censored before the prediction horizon; the latter patients were retained through the survival decision-curve method. Predictions were generated out of fold using stratified 10-fold cross-validation repeated 20 times, providing 20 out-of-fold predictions per patient, which were averaged before calculating net benefit. The full threshold range was 1%–20% in increments of 0.5%; selected clinically illustrative thresholds are reported above. Model B was the age-inclusive clinical reference model containing age, sex, ACS type, diabetes mellitus, hypertension, atrial fibrillation, smoking status, and history of coronary artery disease. Incremental net benefit was calculated by subtracting the net benefit of Model B from that of

each expanded model at the same threshold probability. These internally cross-validated analyses are exploratory and do not establish clinical utility or define treatment thresholds.
Abbreviations: AAR-3, age–anemia–renal dysfunction; ACEF, age–creatinine–ejection fraction score; ACS, acute coronary syndrome; CARE-4, cardiac dysfunction–age–renal dysfunction–anemia; eGFR, estimated glomerular filtration rate; Hb, hemoglobin; LVEF, left ventricular ejection fraction.

Supplementary Table S15. Internal validation of the framework-specific AAR-3 and CARE-4 models

Framework	n	Deaths	Bootstrap resamples	Apparent C-index	C-index optimism	Optimism-corrected C-index	Optimism-corrected calibration slope	Repeated-CV C-index (empirical 95% range)
AAR-3	3544	403	500	0.773	0.005	0.768	0.972	0.767 (0.764–0.770)
CARE-4	3492	394	500	0.800	0.005	0.796	0.975	0.795 (0.792–0.798)

Footnote: Framework-specific optimism correction used 500 bootstrap resamples, and the corresponding 1-year calibration curves in Supplementary Figure S3 were generated using 300 bootstrap resamples. Repeated cross-validation used 10 folds and 200 repetitions. The reported cross-validation ranges represent the empirical 2.5th and 97.5th percentiles of the 200 repeated C-index estimates and should not be interpreted as conventional model-based confidence intervals. These analyses were supportive internal-validation analyses and do not constitute external validation or establish clinical utility.
Abbreviations: AAR-3, Age–Anemia–Renal dysfunction; CARE-4, Cardiac dysfunction–Age–Renal dysfunction–Anemia; CV, cross-validation.

Supplementary Figures

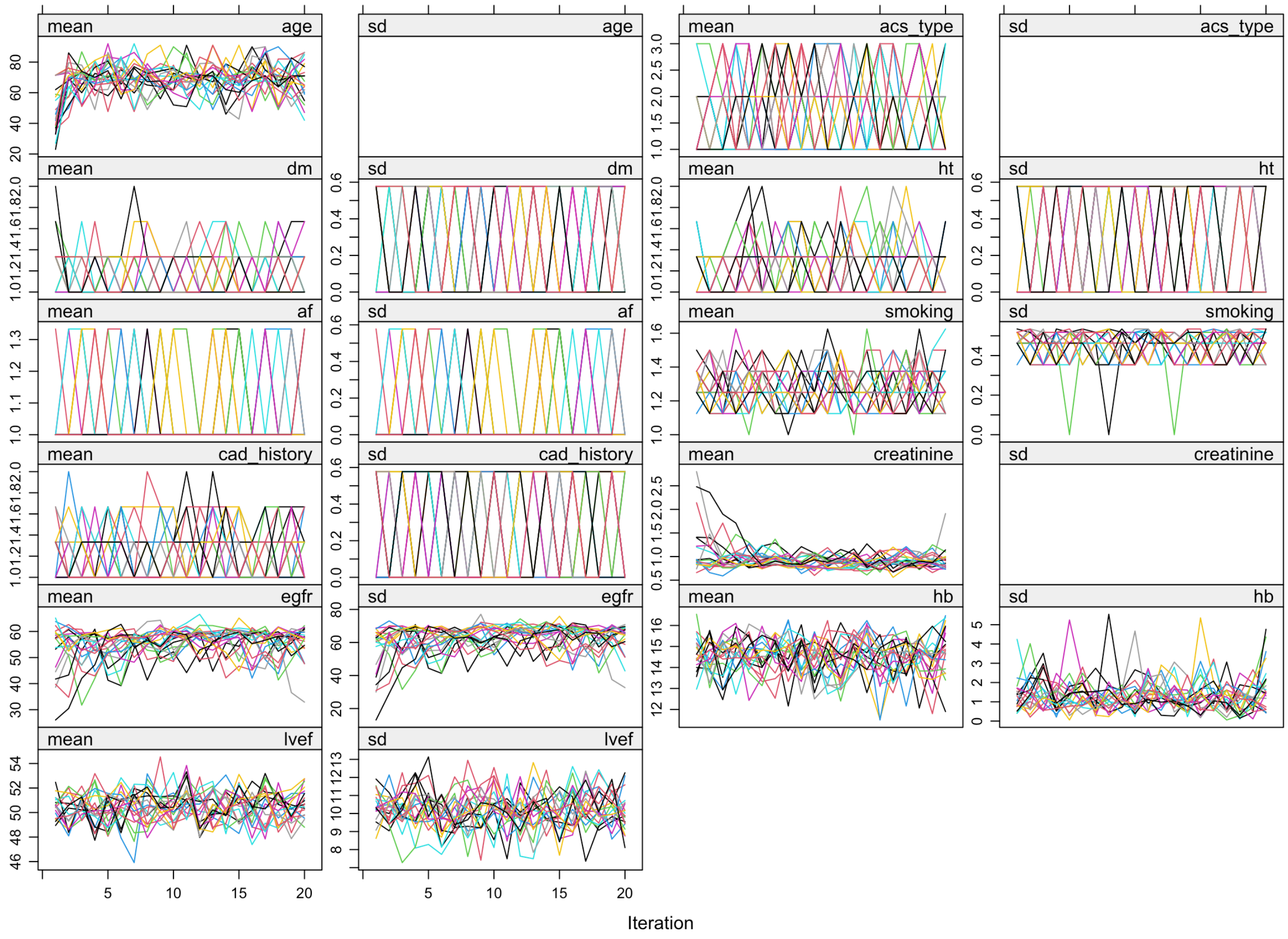

Supplementary Figure S1. Multiple-imputation convergence trace plots

Trace plots show the means and standard deviations of the imputed variables across 20 iterations for each of the 20 imputed datasets. The imputation model included age, ACS type, diabetes mellitus, hypertension, atrial fibrillation, current smoking, history of coronary artery disease, creatinine, estimated glomerular filtration rate, hemoglobin, and left ventricular ejection fraction. Visual inspection did not identify persistent trends or systematic separation among the imputation chains.

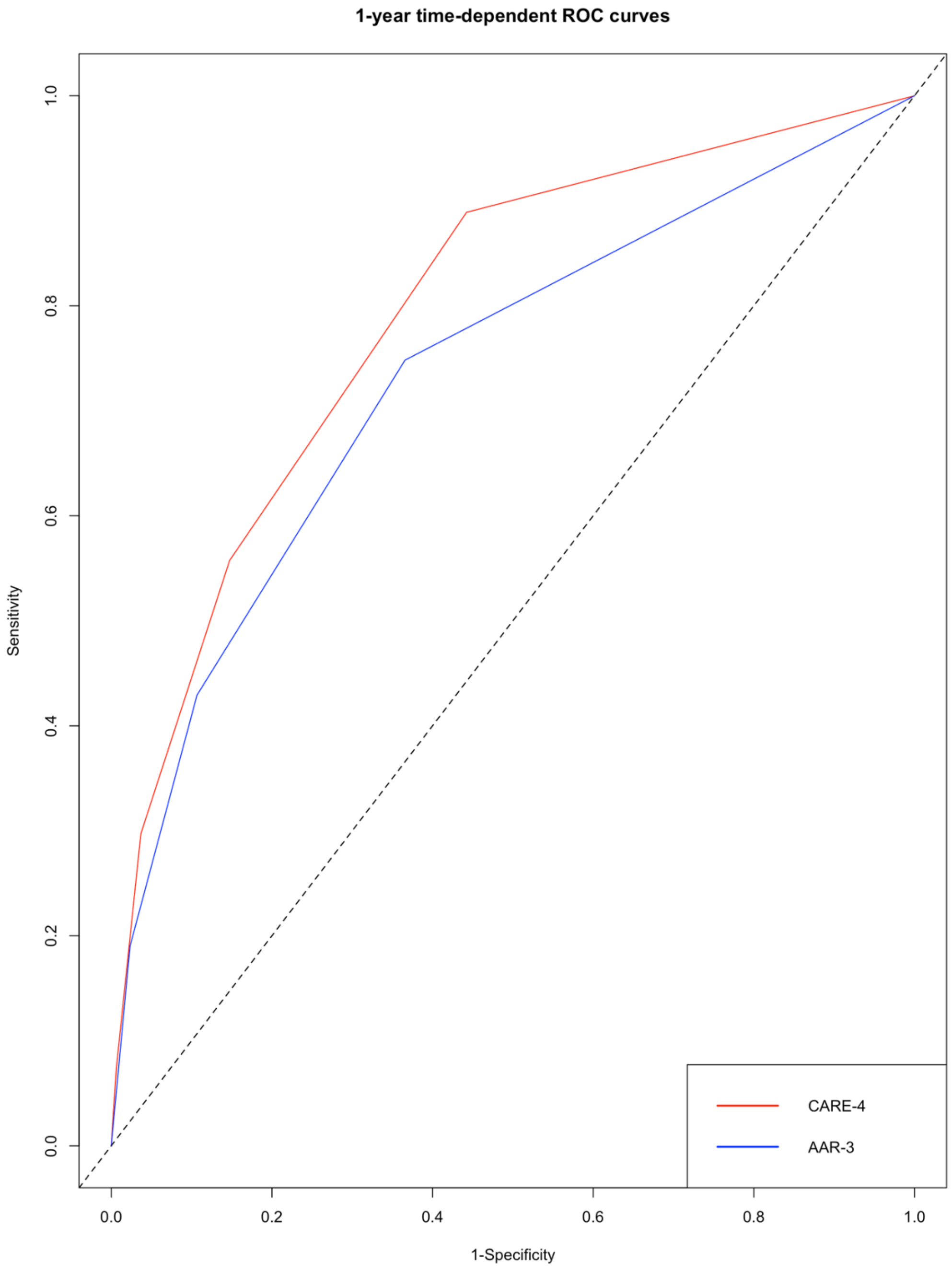

Supplementary Figure S2. Time-dependent ROC curves for 1-year mortality for the framework-specific AAR-3 and CARE-4 models

Time-dependent receiver operating characteristic curves at the 365-day prediction horizon for the framework-specific AAR-3 and CARE-4 models. The curves describe the discrimination of the corresponding models for 1-year all-cause mortality. ROC, receiver operating characteristic.

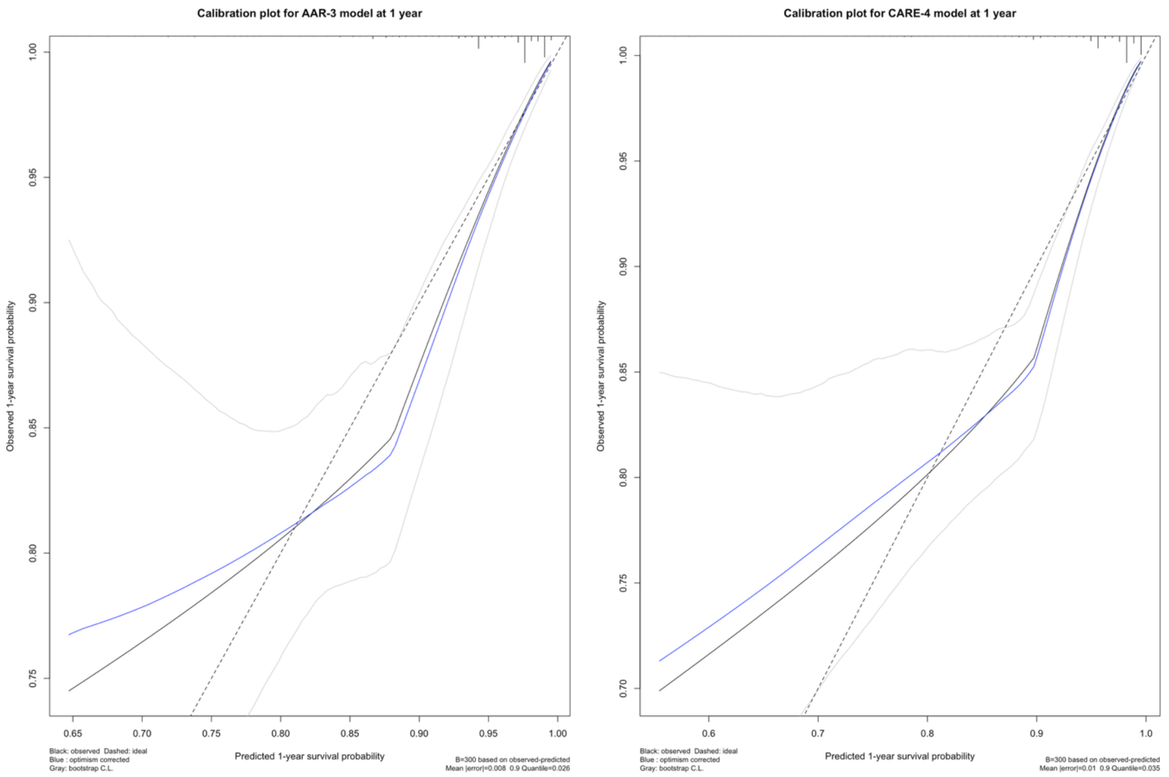

Supplementary Figure S3. Bootstrap-corrected calibration plots for the framework-specific AAR-3 and CARE-4 models

Bootstrap-corrected calibration plots for predicted 1-year survival according to the framework-specific AAR-3 and CARE-4 models based on 300 bootstrap resamples. The solid curves represent the optimism-corrected calibration estimates, and the diagonal line represents perfect agreement between predicted and observed survival probabilities.

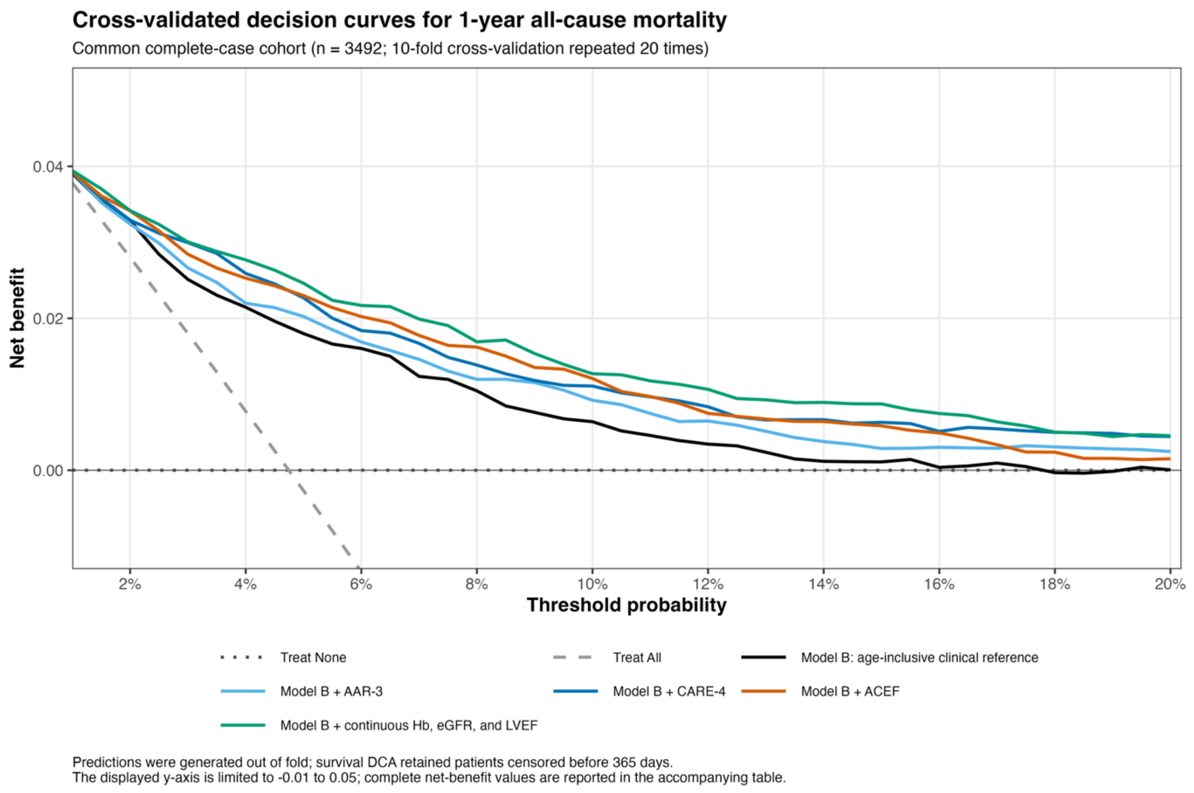

Supplementary Figure S4. Cross-validated survival decision curves for 1-year all-cause mortality

Censoring-aware survival decision curves at the 365-day prediction horizon in the common complete-case cohort (n = 3492). Curves compare the age-inclusive clinical reference model (Model B) with models additionally incorporating AAR-3, CARE-4, ACEF, or continuous hemoglobin, estimated glomerular filtration rate, and left ventricular ejection fraction. Predictions were generated out of fold using stratified 10-fold cross-validation repeated 20 times and were averaged for each patient before calculation of net benefit. The survival decision-curve method retained the 351 patients censored before 365 days; 161 deaths occurred within 365 days. Treat-all and treat-none strategies are shown for comparison. Complete numerical results at selected threshold probabilities are presented in Supplementary Table S14. These internally cross-validated findings are exploratory and do not establish clinical utility or define treatment thresholds. The displayed threshold range was 1%–20% in 0.5% increments.
