# Supplementary material for: Exploratory Mixed-Data Phenomapping and Risk-Enrichment Frameworks for Long-Term Mortality After Acute Coronary Syndrome: A Single-Center Cohort Study
Source: Medicina (Kaunas). 2026 Aug 18;62(8):1586. doi: 10.3390/medicina62081586 (PMC13515590; doi:10.3390/medicina62081586)
Supplement: Supplementary file 1 [file medicina-62-01586-s001.zip › Supplementary File S1 The STROBE reporting checklist.pdf]

The STROBE reporting checklist

For checking that observational epidemiology research articles can be understood and used by everyone

Note

If you have not used a reporting guideline before, read about how and why to use them and check whether STROBE is the most applicable reporting guideline for your work.

Reporting guidelines are most useful when used early in research. When writing a manuscript or application, consider using the Full Guidance where you'll see explanations and examples for each item.

After writing, demonstrate adherence by completing this checklist:

1. Specify where each item is described (see Note 1).
2. Cite this checklist (See Note 2).
3. Include your completed checklist as a supplement when submitting to a journal so that future readers can use it to find information.

	Item Description	Location (or reason for not reporting)
**Title and abstract**		
1a. Indicate the study's design	Indicate the study's design with a commonly used term in the title or the abstract.	**Title; Abstract; Section 2.1.** The title identifies the design, the target population and the outcome: "Exploratory Mixed-Data Phenomapping and Risk-Enrichment Frameworks for Long-Term Mortality After Acute Coronary Syndrome: A Single-Center Cohort Study". Section 2.1 states that this was a retrospective, single-center cohort study of consecutive patients hospitalized with acute coronary syndrome between January 2017 and December 2024.
1b. Abstract	Provide in the abstract an informative and balanced summary of what was done and what was found.	**Abstract.** A structured abstract is provided (Background and Objectives; Materials and Methods; Results;

		Conclusions). It reports each analytic cohort separately (survival cohort n = 3561 after exclusion of 240 patients; Gower–PAM phenomapping cohort n = 3491 after exclusion of 70 patients with incomplete clustering variables; common complete-case cohort n = 3492), the outcome-independent cluster-selection procedure, the age-inclusive clinical reference model, first-year and >365-day landmark hazard ratios with 95% confidence intervals, C-indices with 95% confidence intervals for the reference model and each score, and the negative finding that no score materially improved a model already containing the continuous components. The conclusions state the exploratory nature of the findings and the need for prospective multicenter external validation.
**Introduction**		
2. Background / rationale	Explain the scientific background and rationale for the investigation being reported.	**Introduction, paragraphs 1–3.** The Introduction describes the persisting heterogeneity of long-term outcomes after acute coronary syndrome, the individual associations of routinely measured characteristics with adverse outcomes, and the limited application of data-driven phenotyping to real-world ACS cohorts. It additionally states the methodological rationale for the present work: routine clinical datasets are mixed-type, clustering results depend on the dissimilarity measure and algorithm, and post hoc additive summaries require age-inclusive reference

		models, common-cohort comparison and assessment against models containing the underlying variables continuously.
3. Objectives	State specific objectives, including any prespecified hypotheses.	**Introduction, final paragraph.** Three objectives are stated: (1) to identify outcome-independent patterns of baseline multidomain characteristics using Gower–PAM mixed-data clustering; (2) to describe the associations between the resulting phenotypes and long-term all-cause mortality; and (3) to evaluate the exploratory AAR-3 and CARE-4 frameworks against an age-inclusive clinical reference model and the established ACEF comparator in a common complete-case cohort. It is stated explicitly that AAR-3 and CARE-4 were constructed post hoc and were not prespecified prediction scores; no formal statistical hypotheses were prespecified in a registered protocol.
**Methods**		
4. Study design	Present key elements of study design early in the paper.	**Section 2.1; Title; Abstract.** The study is described in the first sentence of the Methods as a retrospective, single-center cohort study, and the single-center design is also stated in the title and in the Abstract conclusions.
5. Setting	Describe the setting, locations, and relevant dates, including periods of recruitment, exposure, follow-up, and data collection.	**Sections 2.1–2.3.** Setting: a single tertiary cardiovascular center (Izmir Katip Celebi University, Ataturk Training and Research Hospital, Izmir, Türkiye). Accrual: consecutive ACS admissions between January 2017 and December 2024. Data

		collection: institutional electronic medical records; laboratory values were the first measurements obtained at presentation and left ventricular ejection fraction was obtained from transthoracic echocardiography within the first 24–48 h of the index hospitalization. Follow-up: vital status and date of death were ascertained from hospital electronic medical records and the national health information system, with censoring at the last documented follow-up date; median follow-up was 3.35 years.
6a. Eligibility criteria	**Cohort study:** Give the eligibility criteria, and the sources and methods of selection of participants. Describe methods of follow-up. **Case-control study:** Give the eligibility criteria, and the sources and methods of case ascertainment and control selection. Give the rationale for the choice of cases and controls. **Cross-sectional study:** Give the eligibility criteria, and the sources and methods of selection of participants.	**Section 2.1; Section 2.3; Figure 1.**<br>Consecutive patients hospitalized with acute coronary syndrome were eligible. Of 3801 screened patients, 240 were excluded in five mutually exclusive categories: missing mortality status only (n = 102), missing follow-up duration only (n = 37), non-positive follow-up duration only (n = 60), missing mortality status with non-positive follow-up duration (n = 24), and missing mortality status with missing follow-up duration (n = 17). Methods of follow-up and censoring are described in Section 2.3.
6b. Matching criteria	**Cohort study:** For matched studies, give matching criteria and number of exposed and unexposed. **Case-control study:** For matched studies, give matching criteria and the number of controls per case.	**Not applicable.**<br>This was not a matched cohort study; no matching was performed at any stage.
7. Variables	Clearly define all outcomes, exposures, predictors, potential confounders, and effect modifiers. Give diagnostic criteria, if applicable.	**Sections 2.2, 2.3, 2.5, 2.6.**<br>Outcome: all-cause mortality during follow-up. Clustering variables: six continuous (age,

		hemoglobin, eGFR, LVEF, log-transformed NLR, log-transformed SII) and four binary (diabetes mellitus, hypertension, atrial fibrillation, current smoking). Confounders in the primary age-inclusive reference model (Model B): age, sex, ACS subtype, diabetes mellitus, hypertension, atrial fibrillation, current smoking, and prior coronary artery disease; Model C additionally contains continuous hemoglobin, eGFR and LVEF. Diagnostic and definitional criteria are given for ACS subtype (contemporary universal definition and guideline-based classification), eGFR (CKD-EPI), anemia (sex-specific WHO thresholds), renal dysfunction (eGFR <60 mL/min/1.73 m²), systolic dysfunction (LVEF <40%), and the ACEF score. No effect modifiers were formally evaluated.
8. Data sources / measurement	For each variable of interest give sources of data and details of methods of assessment (measurement). Describe comparability of assessment methods if there is more than one group.	**Sections 2.2 and 2.3.** All baseline variables were obtained from institutional electronic medical records. Laboratory values were the first measurements obtained at presentation for the index hospitalization; eGFR was calculated with the CKD-EPI equation; NLR and SII were derived from the admission complete blood count; LVEF was obtained by transthoracic echocardiography within the first 24–48 h. Vital status and date of death were ascertained from two sources, the hospital electronic medical records and the national health information system. Assessment methods

		were identical for all participants, as the cohort was not divided into exposure groups at the time of measurement.
9. Bias	Describe any efforts to address potential sources of bias.	**Sections 2.1, 2.4, 2.6; Section 4.7; Supplementary Tables S1 and S2.**<br>Measures taken to address bias: consecutive inclusion of all eligible admissions; reporting of every exclusion reason separately; reporting of variable-level missingness (Table S1); formal comparison of included versus excluded patients with standardized mean differences (Table S2); multiple imputation by chained equations as a sensitivity analysis for missing covariate data; use of a single common complete-case cohort for all head-to-head score comparisons; use of an age-inclusive model as the primary reference model to avoid confounding by age; construction, cluster-number selection and labeling of the phenotypes without any use of outcome data; bootstrap optimism correction and repeated cross-validation; and out-of-fold cross-validated decision-curve analysis. Residual selection bias, possible informative censoring, survivor selection in the landmark analysis, unmeasured treatment-related variables, and the absence of external validation are acknowledged in Section 4.7.
10. Study size	Explain how the study size was arrived at.	**Sections 2.1 and 3.1.**<br>All consecutive eligible patients available in the institutional database were included; no

		formal sample-size calculation was performed because this was a retrospective observational study. The survival cohort comprised 3561 patients with 407 deaths, the phenomapping cohort 3491 patients with 394 deaths, and the common complete-case cohort 3492 patients with 394 deaths (161 deaths within the first 365 days and 233 deaths after the 365-day landmark among 2977 patients still at risk).
11. Quantitative variables	Explain how quantitative variables were handled in the analyses. If applicable, describe which groupings were chosen, and why.	**Sections 2.2, 2.4, 2.5, 2.6.** NLR and SII were transformed as log(1 + x) because of right-skewed distributions. In the primary Gower–PAM analysis, continuous variables were range-normalized within the Gower dissimilarity, whereas the four binary variables were encoded as two-level nominal factors without asymmetric-binary coding. All ten clustering variables received equal unit weight. Z-score standardization was applied only in the k-means sensitivity analyses. Continuous variables were retained on their original scale in Model C and in the adjusted restricted cubic spline analyses (four knots at the 5th, 35th, 65th and 95th percentiles, with the exposure-specific median as the reference). Dichotomization was used only for the AAR-3 and CARE-4 components, using prespecified clinical thresholds (age ≥75 years; WHO sex-specific anemia; eGFR <60 mL/min/1.73 m²; LVEF <40%) selected for clinical interpretability and not

		optimized against the outcome.
12a. Statistical methods	Describe all statistical methods, including those used to control for confounding.	Kaplan–Meier estimation with log-rank testing; Cox proportional hazards regression with Efron handling of ties; prespecified hierarchical models (Model A, age-omitting and retained only as a historical sensitivity analysis; Model B, the primary age-inclusive reference model; and Model C, additionally containing continuous hemoglobin, eGFR, and LVEF); nested-model comparison by likelihood-ratio testing with Harrell's C-index and model-based 95% confidence intervals, AIC, and BIC; 2000 paired patient-level nonparametric bootstrap resamples to estimate 95% confidence intervals for changes in C-index in the primary Model B comparisons; variable-level and global proportional-hazards assessment using scaled Schoenfeld residuals; first-year and 365-day landmark analyses; adjusted restricted cubic spline analyses; multiple imputation by chained equations with Rubin's rules and D1 tests; bootstrap optimism correction and repeated 10-fold cross-validation; censoring-aware out-of-fold survival decision-curve analysis; and internal temporal-period robustness analyses. Confounding was addressed by multivariable adjustment within the hierarchical models. Descriptive comparisons and the general statistical

		conventions are described in Section 2.7.
12b. Statistical methods – subgroups and interactions	Describe any methods used to examine subgroups and interactions.	**Sections 2.6, 2.7 and 3.6.** No formal subgroup or interaction analyses were performed. Secondary analyses included prespecified time-window analyses (first year versus >365-day landmark) and an internal temporal robustness analysis comparing 2017–2019 with 2020–2024. The 2020 cut-point was chosen pragmatically to create two chronologically distinct subcohorts of broadly comparable size with sufficient observations for stable estimation and was not selected according to outcome or model-performance results. Exploratory inflammatory-marker analyses were also performed. As stated in Section 2.7, these analyses were interpreted as supportive rather than confirmatory and no formal multiplicity correction was applied.
12c. Statistical methods – missing data	Explain how missing data were addressed.	**Sections 2.1, 2.6, 3.1 and 3.6; Supplementary Tables S1, S2 and S8.** Variable-level missingness is reported for both the initial cohort (n = 3801) and the survival cohort (n = 3561) in Table S1. Patients with unascertainable mortality status or invalid follow-up were excluded and were not reintroduced through imputation; their characteristics are compared with those of included patients in Table S2. Primary analyses were complete-case and, for all head-to-head comparisons,

		were restricted to a single common complete-case cohort. Multiple imputation by chained equations (20 datasets, 20 iterations; predictive mean matching for continuous variables, binary and polytomous logistic regression for categorical variables; event indicator and Nelson–Aalen cumulative hazard as auxiliary predictors; outcomes not imputed) was performed as a sensitivity analysis and produced estimates essentially identical to the complete-case results. It is stated explicitly that imputation cannot correct selection bias arising from missing outcome or follow-up information.
12di. Statistical methods – loss to follow-up	**Cohort study:** If applicable, describe how loss to follow-up was addressed.	**Sections 2.3, 3.1 and 4.7.** Patients without a documented death in either data source were confirmed alive at their last available follow-up date and right-censored on that date, with no assumption made about vital status thereafter. Patients whose mortality status or follow-up duration could not be ascertained were excluded and are characterized in Supplementary Table S2. The possibility of informative censoring, and survivor-selection effects in the 365-day landmark analysis, are acknowledged in Section 4.7.
12dii. Statistical methods – matching cases and controls	**Case-control study:** If applicable, explain how matching of cases and controls was addressed.	**Not applicable.** This was not a case-control study; no matching of cases and controls was performed.
12diii. Statistical methods – sampling	**Cross-sectional study:** If applicable, describe analytical methods taking account of sampling	**Not applicable.** This was not a cross-sectional

strategy	strategy.	study; no complex sampling strategy was used, as all consecutive eligible patients were included.
12e. Statistical methods – sensitivity analyses	Describe any sensitivity analyses.	**Sections 2.4, 2.6, 3.3 and 3.6; Supplementary Tables S6, S8, S9, S11, S13, S14 and S15.** Sensitivity analyses comprised: standardized mixed-variable and continuous-only k-means clustering compared with the primary Gower–PAM solution using adjusted Rand indices; an expanded clinical adjustment model for the phenotypes; the historical age-omitting reference model (Model A); the continuous-component model (Model C); multiple imputation by chained equations; first-year and 365-day landmark time-window analyses; internal temporal-period analyses (2017–2019 versus 2020–2024); bootstrap optimism correction and repeated 10-fold cross-validation; exploratory inflammatory-marker analyses; and cross-validated survival decision-curve analysis.
**Results**		
13a. Participant numbers	Report the numbers of individuals at each stage of the study—e.g., numbers potentially eligible, examined for eligibility, confirmed eligible, included in the study, completing follow-up, and analysed; Consider use of a flow diagram.	**Section 3.1; Figure 1.** 3801 patients were screened and 240 were excluded, leaving 3561 in the survival cohort. Subsequent analytic cohorts were: Gower–PAM phenomapping cohort, n = 3491; AAR-3 descriptive cohort, n = 3557; CARE-4 descriptive cohort, n = 3505; and the common complete-case cohort used for all head-to-head comparisons, n = 3492. In the landmark analysis, 2977 patients remained alive and

		under observation beyond 365 days.
13b. Participants – non-participation	Give reasons for non-participation at each stage.	**Section 2.1; Section 3.1; Figure 1.**<br>The 240 exclusions are reported as five mutually exclusive categories with individual counts (n = 102, 37, 60, 24 and 17), both in the text and in the flow diagram. A comparison of included versus excluded patients, including standardized mean differences, is provided in Supplementary Table S2 and summarized in Section 3.1; excluded patients were older, more frequently female, had a higher comorbidity burden and lower eGFR.
13c. Participants – flow diagram	Consider use of a flow diagram.	**Figure 1.**<br>A study flow diagram is provided showing screening, the five mutually exclusive exclusion categories, the survival cohort, the framework-specific descriptive cohorts, the outcome-independent Gower–PAM phenomapping cohort, and the common complete-case cohort used for the primary head-to-head comparison.
14a. Descriptive data – participant characteristics	Give characteristics of study participants (e.g., demographic, clinical, social) and information on exposures and potential confounders. Present the information in a table.	**Section 3.2; Table 1; Supplementary Tables S3 and S5.**<br>Baseline demographic, clinical, laboratory, inflammatory and echocardiographic characteristics are presented overall and across CARE-4 categories (Table 1), across AAR-3 categories (Supplementary Table S3) and by Gower–PAM phenotype (Supplementary Table S5). Comparisons of variables that

		structurally define the scores or the phenotypes are explicitly labeled as descriptive rather than inferential, and no p-values are reported for the phenotype-defining variables.
14b. Descriptive data – missing data	Indicate the number of participants with missing data for each variable of interest.	**Section 3.1; Supplementary Table S1.**<br>Variable-level missingness, with counts and percentages, is reported for the initial cohort (n = 3801) and the survival cohort (n = 3561); for example, mortality status was missing in 143 patients (3.76%), follow-up duration in 54 (1.42%) and LVEF in 238 (6.26%) of the initial cohort. The resulting differences in analytic cohort size are reported in Section 3.1 and in Figure 1.
14c. Descriptive data – follow-up time	**Cohort study:** Summarise follow-up time—e.g., average and total amount.	**Sections 3.1, 3.2 and 3.5; Table 1; Supplementary Table S5.**<br>Median follow-up was 3.35 years overall and is additionally reported by CARE-4 category (Table 1) and by phenotype (Supplementary Table S5). The populations and event counts for the first-year and >365-day landmark analysis windows are reported in Section 3.5.
15. Outcome data	**Cohort study:** Report numbers of outcome events or summary measures over time. **Case-control study:** Report numbers in each exposure category, or summary measures of exposure. **Cross-sectional study:** Report numbers of outcome events or summary measures.	**Sections 3.1, 3.3 and 3.5.**<br>A total of 407 all-cause deaths occurred in the survival cohort. Event counts are reported for every analytic cohort: 394 deaths in the phenomapping cohort (12.1%, 4.2% and 19.2% in phenotypes A, B and C), 406 in the AAR-3 cohort, 397 in the CARE-4 cohort, and 394 in the common complete-case cohort, of which 161 occurred within 365 days and 233 after the landmark.
16a. Main results	Give unadjusted estimates and, if applicable,	**Sections 3.4 and 3.5; Table 2;**

	confounder-adjusted estimates and their precision (e.g., 95% confidence intervals). Make clear which confounders were adjusted for and why they were included.	**Supplementary Tables S6, S7, S9, S10 and S11.**<br>Unadjusted and adjusted hazard ratios with 95% confidence intervals and p-values are reported for the Gower–PAM phenotypes and for AAR-3, CARE-4 and ACEF. The primary reference model (Model B) is adjusted for age, sex, ACS subtype, diabetes mellitus, hypertension, atrial fibrillation, current smoking and prior coronary artery disease; the age-omitting Model A is retained only as a labeled historical sensitivity analysis, and Model C additionally contains continuous hemoglobin, eGFR and LVEF. Because the global proportional hazards assumption was not satisfied over full follow-up, the first-year and 365-day landmark estimates are presented as the principal effect estimates and the full-follow-up hazard ratios are labeled as average associations over time. Harrell's C-indices are reported with 95% confidence intervals, and the primary changes in C-index are accompanied by 95% paired-bootstrap confidence intervals based on 2000 patient-level resamples.
16b. Main results – category boundaries	Report category boundaries when continuous variables were categorised.	**Section 2.5; Table 1; Supplementary Table S3.**<br>AAR-3 ranges from 0 to 3 and CARE-4 from 0 to 4. Each component contributes one point using prespecified clinical thresholds: age ≥75 years; WHO sex-specific anemia (hemoglobin <13.0 g/dL in men, <12.0 g/dL in women); eGFR <60 mL/min/1.73 m²; and

		LVEF <40%. The rationale for each threshold and for equal weighting is stated, together with the explicit statement that no regression coefficients, outcome-derived weights or data-dependent cutoffs were used. The corresponding continuous variables are additionally analysed on their original scale in Model C and in the restricted cubic spline analyses.
16c. Main results – risk	If relevant, consider translating estimates of relative risk into absolute risk for a meaningful time period.	**Section 3.2; Table 1; Figures 2 and 3; Supplementary Tables S3 and S5.** Absolute mortality is reported across CARE-4 categories (3.4% to 51.4%), across AAR-3 categories (4.8% to 42.4%) and across the Gower–PAM phenotypes (12.1%, 4.2% and 19.2%). Kaplan–Meier curves with numbers at risk are presented in Figures 2 and 3. The CARE-4 = 4 category contained only 35 patients and is flagged for cautious interpretation in the text, in the Table 1 footnote and in the Figure 2 legend.
17. Other analyses	Report other analyses done—e.g., analyses of subgroups and interactions, and sensitivity analyses.	**Section 3.6; Figure 4; Supplementary Tables S8, S12, S13, S14 and S15; Supplementary Figures S1–S4.** Other analyses reported: adjusted restricted cubic spline analyses with overall and nonlinearity p-values; bootstrap optimism correction, bootstrap-corrected calibration and repeated 10-fold cross-validation; the multiple-imputation sensitivity analysis with pooled estimates and convergence diagnostics; cluster bootstrap stability and cross-method agreement

		(adjusted Rand indices); the expanded clinical adjustment model for the phenotypes; exploratory inflammatory-marker analyses; internal temporal-period analyses; and censoring-aware cross-validated survival decision-curve analysis across threshold probabilities of 1%–20% in 0.5% increments.
**Discussion**		
18. Key results	Summarise key results with reference to study objectives.	**Section 4.1.**<br>The key results are summarized in two short paragraphs with reference to the study objectives: outcome-independent Gower–PAM analysis identified three internally stable but method-dependent phenotypic patterns whose effect was attenuated after expanded clinical adjustment; and AAR-3, CARE-4 and ACEF were each associated with mortality and improved fit relative to the age-inclusive reference model, but none materially improved a model already containing the underlying continuous domains.
19. Limitations	Discuss limitations of the study, taking into account sources of potential bias or imprecision. Discuss both direction and magnitude of any potential bias.	**Section 4.7.**<br>Nine numbered limitations are discussed: the retrospective single-center design and residual confounding; selection bias arising from the 240 exclusions, supported by the included-versus-excluded comparison; censoring at the last documented follow-up with possible informative censoring, and survivor selection in the landmark analysis; changes in treatment practice across 2017–2024

		together with unavailable treatment-related variables; the all-cause endpoint without cause-specific mortality; single-timepoint early LVEF measurement variability and the absence of direct frailty, functional, nutritional, cognitive and sarcopenia measures; the exploratory and method-dependent nature of the phenomapping, including the influence of smoking on the selected partition; the post hoc construction and partial circularity of AAR-3 and CARE-4 with the resulting possibility of optimism, and the impossibility of direct GRACE comparison; and the absence of any independent external validation cohort.
20. Interpretation	Give a cautious overall interpretation considering objectives, limitations, multiplicity of analyses, results from similar studies, and other relevant evidence.	**Sections 4.2–4.6.**<br>The results are interpreted as demonstrating statistical co-occurrence and association rather than mechanism. The established biological literature is explicitly separated from what the present data can support, the attenuation of the phenotype effect after expanded adjustment and the absence of incremental value beyond the continuous components are emphasized, and the clinical implications are restricted to hypothesis generation. Objectives, limitations, multiplicity of secondary analyses and evidence from similar studies are taken into account throughout.
21. Generalisability	Discuss the generalisability (external validity) of the study results.	**Sections 4.5 and 4.7; Conclusions.**<br>Generalizability is explicitly limited by the single-center

		Turkish tertiary-care setting, the retrospective design, the method-dependence of the phenotype assignments and the absence of external validation. Prospective multicenter external validation, comparison with established acute coronary syndrome risk models, and treatment-informed clinical utility studies are stated as prerequisites before any clinical application.
**Other information**		
22. Funding	Give the source of funding and the role of the funders for the present study and, if applicable, for the original study on which the present article is based.	**Funding statement.** This research received no external funding. Because no funder was involved, the role of the funding source in the design, conduct, analysis, interpretation or reporting of the study is not applicable.

1 How to specify where content is

Tell the reader where they can find information. E.g.,

- Results; paragraph 2
- Methods, Participants; paragraphs 1 & 2.
- Table 3
- Supplement B, para. 4

If you have chosen not to describe an item, explain why. You can do this in the checklist, or as a note below it.

You can describe items in the article body, or in tables, figures, or supplementary materials, and should prioritize items you feel are most important to your intended audience. The order of items in your manuscript does not need to match the order of items in this checklist. You can decide how best to structure your work.

2 How to cite

Describe how you used STROBE at the end of your Methods section, referencing the resources you used e.g.,

> ‘We used the STROBE reporting guideline(1) to draft this manuscript, and the STROBE reporting checklist(2) when editing, included in supplement A’

If you use a reporting checklist, remember to include it as a supplement when publishing so that readers can easily find information and see how you have interpreted the guidance.

1. Elm E von, Altman DG, Egger M, Pocock SJ, Gøtzsche PC, Vandenbroucke JP, et al. The strengthening the reporting of observational studies in epidemiology (STROBE) statement: Guidelines for reporting observational studies. Annals of Internal Medicine [Internet]. 2007 Oct;147(8):573–7. Available from: https://www.acpjournals.org/doi/10.7326/0003-4819-147-8-200710160-00010

2. Elm E von, Altman DG, Egger M, Pocock SJ, Gøtzsche PC, Vandenbroucke JP, et al. The STROBE reporting checklist. In: Harwood J, Albury C, Beyer J de, Schlüssel M, Collins G, editors. The EQUATOR network reporting guideline platform [Internet]. The UK EQUATOR Centre; 2025. Available from: https:/resources.equator-network.org/reporting-guidelines/strobe/strobe-checklist.docx
