# Supplementary material for: Exploratory Mixed-Data Phenomapping and Risk-Enrichment Frameworks for Long-Term Mortality After Acute Coronary Syndrome: A Single-Center Cohort Study
Source: Medicina (Kaunas). 2026 Aug 18;62(8):1586. doi: 10.3390/medicina62081586 (PMC13515590; doi:10.3390/medicina62081586)
Supplement: Supplementary file 1 [file medicina-62-01586-s001.zip › Supplementary File S2 TRIPOD-AI checklist.pdf]

TRIPOD+AI

This study was not designed as a formal de novo multivariable clinical prediction model development study or an external validation study. TRIPOD+AI items were considered where applicable to improve transparent reporting of data-driven phenotyping, vulnerability framework operationalization, and prognostic performance assessment.

Section/Topic	Item	Development / evaluation[1]	Checklist item	Reported on page
**TITLE**				
Title	1	D;E	Identify the study as developing or evaluating the performance of a multivariable prediction model, the target population, and the outcome to be predicted	**Title.** "Exploratory Mixed-Data Phenomapping and Risk-Enrichment Frameworks for Long-Term Mortality After Acute Coronary Syndrome: A Single-Center Cohort Study". The title identifies the target population (patients hospitalized with acute coronary syndrome), the outcome (long-term mortality) and the exploratory nature of the work. The study is not presented as the development or external validation of a multivariable clinical prediction model: AAR-3 and CARE-4 were constructed post hoc as transparent additive summaries and were evaluated internally, and the phenotypes are descriptive statistical partitions rather than a prediction algorithm.
**ABSTRACT**				
Abstract	2	D;E	See TRIPOD+AI for Abstracts checklist	**Abstract.** A structured abstract reports the design, the accrual period, each analytic cohort with its exclusions (survival cohort n = 3561; Gower–PAM phenomapping cohort n = 3491; common complete-case cohort n = 3492), the outcome-independent cluster-selection procedure, the age-inclusive clinical reference model, first-year and >365-day landmark hazard ratios with 95% confidence intervals, C-indices with 95% confidence intervals for the reference model and each score, the finding that no score materially improved a model containing the continuous components, and the conclusion that prospective multicenter external validation is required before clinical application.
**INTRODUCTION**				
Background	3a	D;E	Explain the healthcare context (including whether diagnostic or prognostic) and rationale for developing or evaluating the prediction model, including references to existing models	**Introduction, paragraphs 1–3.** The Introduction sets out the prognostic (not diagnostic) context of residual long-term risk after acute coronary syndrome, the individual associations of routinely measured characteristics with outcome, and the limited application of data-driven phenotyping to real-world ACS cohorts. Existing approaches are referenced, including the GRACE score and heart-failure phenomapping studies, and ACEF is introduced as the established comparator that could be calculated in the available data.
	3b	D;E	Describe the target population and the intended purpose of the prediction model in the context of the care pathway, including its intended users (e.g., healthcare professionals, patients, public)	**Introduction, final paragraph; Sections 4.5 and 4.7.** Target population: adults hospitalized with acute coronary syndrome at a tertiary cardiovascular center. Intended purpose in this study: exploratory description of co-occurring baseline characteristics and evaluation of two post hoc additive summaries against an age-inclusive reference model. The manuscript states explicitly that no management strategy based on the scores or phenotypes was evaluated, that no score

				threshold was linked to a clinical action, and that the frameworks are not intended for use by clinicians or patients in their current form.
	3c	D;E	Describe any known health inequalities between sociodemographic groups	**Not formally assessed.** The study was not designed to investigate health inequalities between sociodemographic groups, and variables such as socioeconomic status, education, ethnicity and insurance status were not available in the retrospective records. Sex was recorded as a biological variable from electronic health records and was included in all multivariable models; gender identity was not available. This limitation is acknowledged in Section 2.1.
Objectives	4	D;E	Specify the study objectives, including whether the study describes the development or validation of a prediction model (or both)	**Introduction, final paragraph.** The objectives were: (1) to identify outcome-independent patterns of baseline multidomain characteristics using Gower–PAM mixed-data clustering; (2) to describe the associations between the resulting phenotypes and long-term all-cause mortality; and (3) to evaluate the exploratory AAR-3 and CARE-4 frameworks against an age-inclusive clinical reference model and the ACEF comparator in a common complete-case cohort. The manuscript states that this was neither a formal de novo prediction-model development study nor an external validation study; the frameworks were constructed post hoc and evaluated within the same cohort.
**METHODS**				
Data	5a	D;E	Describe the sources of data separately for the development and evaluation datasets (e.g., randomised trial, cohort, routine care or registry data), the rationale for using these data, and representativeness of the data	**Section 2.1.** Routine-care data from a retrospective, single-center cohort of consecutive patients hospitalized with acute coronary syndrome at a tertiary cardiovascular center. Routine-care data were used because the objective was to examine characteristics that are actually available at the bedside during an index ACS hospitalization. Representativeness is limited: the cohort derives from one tertiary center in Türkiye, and Section 4.7 states that the findings may not generalize to hospitals with different patient characteristics, treatment practices, follow-up systems or resources.
	5b	D;E	Specify the dates of the collected participant data, including start and end of participant accrual; and, if applicable, end of follow-up	**Sections 2.1 and 2.3.** Participant accrual: consecutive admissions from 1 January 2017 to 31 December 2024. Follow-up: from the index hospitalization to death or the last documented follow-up date, ascertained from hospital electronic medical records and the national health information system; median follow-up was 3.35 years. The cohort was additionally divided into two non-overlapping calendar periods (2017–2019 and 2020–2024) for the internal temporal robustness analysis.
Participants	6a	D;E	Specify key elements of the study setting (e.g., primary care, secondary care, general population) including the number and location of centres	**Section 2.1.** Secondary and tertiary care; one center: Izmir Katip Celebi University, Ataturk Training and Research Hospital, Izmir, Türkiye.
	6b	D;E	Describe the eligibility criteria for study participants	**Section 2.1; Figure 1.** Consecutive patients hospitalized with acute coronary syndrome were eligible. Of 3801

				screened patients, 240 were excluded in five mutually exclusive categories: missing mortality status only (n = 102), missing follow-up duration only (n = 37), non-positive follow-up duration only (n = 60), missing mortality status with non-positive follow-up duration (n = 24), and missing mortality status with missing follow-up duration (n = 17). A further 70 patients lacked at least one clustering variable and were not included in the phenomapping cohort.
	6c	D;E	Give details of any treatments received, and how they were handled during model development or evaluation, if relevant	**Sections 2.2 and 4.7.** Detailed treatment-related variables—discharge pharmacotherapy, completeness of revascularization, procedural complexity, longitudinal medication adherence, lipid-lowering intensity, cardiac rehabilitation participation and serial biomarker trajectories—were not systematically retrievable across the study period and therefore could not be included in any model. Because treatment practices may also have changed across 2017–2024, the limitations state that unmeasured treatment differences and secular changes may have contributed to the observed associations, and that the findings describe associations rather than treatment-independent effects.
Data preparation	7	D;E	Describe any data pre-processing and quality checking, including whether this was similar across relevant sociodemographic groups	**Sections 2.1, 2.2, 2.4; Supplementary Tables S1 and S2.** NLR and SII were transformed as log(1 + x) because of right-skewed distributions. In the primary Gower–PAM analysis, continuous variables were range-normalized within the Gower dissimilarity, whereas diabetes mellitus, hypertension, atrial fibrillation, and current smoking were encoded as two-level nominal factors without asymmetric-binary coding. No custom variable weights were specified, and all ten clustering variables therefore received equal unit weight. Z-score standardization was used only in the k-means sensitivity analyses. Quality checking comprised reporting of variable-level missingness for the initial and survival cohorts (Table S1), exclusion of records with non-positive follow-up duration, and a formal comparison of included versus excluded patients with standardized mean differences (Table S2). Data pre-processing was identical for all patients; it was not stratified by, or differentiated across, sociodemographic groups.
Outcome	8a	D;E	Clearly define the outcome that is being predicted and the time horizon, including how and when assessed, the rationale for choosing this outcome, and whether the method of outcome assessment is consistent across sociodemographic groups	**Sections 2.3, 2.6 and 3.5.** Outcome: all-cause mortality. Time horizon: full follow-up (median 3.35 years), with prespecified time-window analyses for the first 365 days and for the period beyond a 365-day landmark; 1-year absolute risk was used for the decision-curve analysis. All-cause mortality was chosen because it is objective, complete in the national health information system and not subject to adjudication bias; cause-specific mortality was not systematically available, which is stated as a limitation. Outcome ascertainment used the same two sources for all patients and was therefore consistent across sociodemographic groups.

	8b	D;E	If outcome assessment requires subjective interpretation, describe the qualifications and demographic characteristics of the outcome assessors	**Not applicable.** All-cause mortality was ascertained from hospital electronic medical records and the national health information system and did not require subjective interpretation or adjudication by assessors.
	8c	D;E	Report any actions to blind assessment of the outcome to be predicted	**Not applicable / partially addressed by design.** Outcome ascertainment was registry-based and required no interpretation, so blinding was not relevant. However, the clustering procedure itself was blinded to outcome by design: mortality status and follow-up duration were excluded from the clustering input and were not used to select the number of clusters, to construct the clusters, to order them or to assign phenotype labels; outcomes were examined only after the clustering and labeling had been finalized.
Predictors	9a	D	Describe the choice of initial predictors (e.g., literature, previous models, all available predictors) and any pre-selection of predictors before model building	**Introduction, paragraph 3; Sections 2.2 and 2.5.** The ten clustering variables were selected a priori on the basis of biological plausibility, routine availability during an index ACS hospitalization and prior literature, and not by outcome-driven screening. AAR-3 and CARE-4 were then constructed post hoc, after inspection of the domains represented in the phenomapping analysis, using clinically familiar thresholds; they were not prespecified, not algorithmically derived from the cluster assignments and not optimized against mortality. The manuscript states that this creates partial circularity and increases the possibility of optimistic performance estimates.
	9b	D;E	Clearly define all predictors, including how and when they were measured (and any actions to blind assessment of predictors for the outcome and other predictors)	**Section 2.2.** All variables are defined with their measurement timing: laboratory values were the first measurements obtained at presentation for the index hospitalization; eGFR was calculated with the CKD-EPI equation; NLR was the absolute neutrophil count divided by the absolute lymphocyte count and SII was platelet count × neutrophil count / lymphocyte count, both from the admission complete blood count; LVEF was obtained by transthoracic echocardiography within the first 24–48 h. Comorbidities were taken from the electronic record at the index admission. Because all predictors were recorded during routine care before the outcome occurred, assessment of predictors was inherently blinded to the outcome.
	9c	D;E	If predictor measurement requires subjective interpretation, describe the qualifications and demographic characteristics of the predictor assessors	**Partially applicable.** Laboratory and demographic variables were measured objectively. Left ventricular ejection fraction, however, is derived from transthoracic echocardiography performed and interpreted by cardiologists during routine clinical care; individual operator qualifications and demographic characteristics were not recorded in the retrospective dataset. Section 4.7 acknowledges that LVEF may have been influenced by acute loading conditions, image quality and interobserver or intraobserver

				variability.
Sample size	10	D;E	Explain how the study size was arrived at (separately for development and evaluation), and justify that the study size was sufficient to answer the research question. Include details of any sample size calculation	**Sections 2.1 and 3.1.** No formal sample-size calculation was performed; all consecutive eligible patients in the institutional database were included. The resulting numbers are: survival cohort 3561 patients with 407 deaths; phenomapping cohort 3491 patients with 394 deaths; common complete-case cohort 3492 patients with 394 deaths, comprising 161 deaths within the first 365 days and 233 deaths after the landmark among 2977 patients. The number of events relative to the number of covariates in the reference models is comfortably above conventional events-per-variable recommendations for the full-follow-up analyses, although the first-year window is smaller and its estimates are correspondingly less precise.
Missing data	11	D;E	Describe how missing data were handled. Provide reasons for omitting any data	**Sections 2.1, 2.6, 3.1 and 3.6; Supplementary Tables S1, S2 and S8.** Variable-level missingness is reported for the initial and survival cohorts (Table S1). Patients whose mortality status or follow-up duration could not be ascertained were omitted and were not reintroduced by imputation; their characteristics are compared with those of included patients using standardized mean differences (Table S2). Primary analyses were complete-case, with all head-to-head comparisons restricted to a single common complete-case cohort. Multiple imputation by chained equations was performed as a sensitivity analysis (20 datasets, 20 iterations; predictive mean matching for continuous variables, binary and polytomous logistic regression for categorical variables; event indicator and Nelson–Aalen cumulative hazard as auxiliary predictors; survival outcomes not imputed; Rubin's rules and D1 tests for pooling). Pooled estimates were essentially identical to the complete-case results, and it is stated that imputation cannot correct selection bias arising from missing outcome or follow-up information.
Analytical methods	12a	D	Describe how the data were used (e.g., for development and evaluation of model performance) in the analysis, including whether the data were partitioned, considering any sample size requirements	**Sections 2.1, 2.6 and 3.6.** The data were not partitioned into separate development and evaluation sets, because no model coefficients were estimated for AAR-3 or CARE-4 and no independent external cohort was available. All head-to-head comparisons were performed in a single common complete-case cohort of 3492 patients. Internal performance was assessed by bootstrap optimism correction (500 resamples), repeated 10-fold cross-validation (200 repetitions) and out-of-fold cross-validated decision-curve analysis. In addition, the common cohort was divided into two chronologically distinct calendar periods (2017–2019 and 2020–2024) as an internal temporal robustness analysis. The 2020 cut-point was selected pragmatically to obtain two subcohorts of broadly comparable size with sufficient observations for stable estimation and

				was not selected according to outcome or model-performance results. This analysis was not considered a formal derivation–validation split or external validation.
	12b	D	Depending on the type of model, describe how predictors were handled in the analyses (functional form, rescaling, transformation, or any standardisation).	**Sections 2.4, 2.5 and 2.6.** In the primary Gower–PAM clustering, continuous variables were range-normalized within the Gower dissimilarity and binary variables were treated as categorical factors, with equal weight for all ten variables; NLR and SII were log(1 + x)-transformed. In the Cox models, age, hemoglobin, eGFR and LVEF were retained on their original continuous scale in Model C, and their functional forms were examined using adjusted restricted cubic splines with four knots at the 5th, 35th, 65th and 95th percentiles, referenced to the exposure-specific median. Dichotomization occurred only within AAR-3, CARE-4 and the creatinine component of ACEF, using prespecified clinical thresholds; ACEF was calculated as age divided by LVEF with one additional point for creatinine >2.0 mg/dL.
	12c	D	Specify the type of model, rationale[2], all model-building steps, including any hyperparameter tuning, and method for internal validation	**Sections 2.4 and 2.6.** Clustering: partitioning around medoids applied to a Gower dissimilarity matrix. Candidate solutions with k = 2, 3 and 4 were evaluated using a prespecified, outcome-independent rule—eligibility required every cluster to contain at least 10% of the cohort and to have a bootstrap Jaccard coefficient of at least 0.75 across 100 resamples, after which the eligible solution maximizing the average silhouette width was selected, with smaller k preferred only in the event of an exact tie. No hyperparameter tuning was performed against the outcome. Standardized mixed-variable and continuous-only k-means were run as method-sensitivity analyses with k fixed to the primary selection and 50 random initializations. Survival modelling used Cox proportional hazards regression with Efron tie handling in prespecified hierarchical models (Model A historical, Model B primary, Model C continuous-component). Internal validation comprised bootstrap optimism correction, bootstrap-corrected calibration, repeated 10-fold cross-validation, cross-validated decision-curve analysis, multiple imputation and internal temporal-period analyses.
	12d	D;E	Describe if and how any heterogeneity in estimates of model parameter values and model performance was handled and quantified across clusters (e.g., hospitals, countries). See TRIPOD-Cluster for additional considerations[3]	**Not applicable as a multicenter analysis.** The data originate from a single center, so no between-center or between-country heterogeneity could be quantified. As the closest available analogue, the common complete-case cohort was divided into two non-overlapping calendar periods (2017–2019 and 2020–2024) and models were fitted separately within each period, with results reported in Supplementary Table S13.
	12e	D;E	Specify all measures and plots used (and their rationale) to evaluate model performance (e.g., discrimination, calibration, clinical utility) and, if relevant, to compare multiple models	**Section 2.6.** Performance and comparison measures included

				adjusted hazard ratios with 95% confidence intervals; likelihood-ratio tests for nested models; Harrell's C-index with model-based 95% confidence intervals; ΔC-index relative to the corresponding reference model; Akaike and Bayesian information criteria; bootstrap-corrected calibration curves and calibration slopes at 1 year; time-dependent receiver operating characteristic curves at 365 days; and censoring-aware survival decision-curve analysis using out-of-fold predictions. For the primary Model B comparisons, 95% confidence intervals for changes in C-index were estimated using 2000 paired patient-level nonparametric bootstrap resamples. Decision-curve analysis was evaluated over threshold probabilities from 1% to 20% in 0.5% increments and was interpreted as exploratory decision-analytic performance rather than evidence of clinical utility or treatment-guiding thresholds.
	12f	E	Describe any model updating (e.g., recalibration) arising from the model evaluation, either overall or for particular sociodemographic groups or settings	**Not applicable.** No model updating, recalibration or revision was performed; AAR-3 and CARE-4 contain no estimated parameters that could be updated.
	12g	E	For model evaluation, describe how the model predictions were calculated (e.g., formula, code, object, application programming interface)	**Not applicable.** This study did not externally evaluate a previously developed prediction model. Where absolute risk predictions were required for the decision-curve analysis, they were generated out of fold from Cox models fitted within the same cohort, as described in Section 2.6.
Class imbalance	13	D;E	If class imbalance methods were used, state why and how this was done, and any subsequent methods to recalibrate the model or the model predictions	**Not applicable.** No class-imbalance correction was applied. Analyses were time-to-event models rather than binary classifiers, and the observed event proportion (407 deaths in 3561 patients) did not warrant resampling or reweighting.
Fairness	14	D;E	Describe any approaches that were used to address model fairness and their rationale	**Not formally assessed.** No formal algorithmic fairness assessment was performed. Sex was included as a covariate in all multivariable models, but performance was not compared across sex, age or other sociodemographic strata, and the variables required for such an assessment (including socioeconomic and ethnicity data) were not available. Because the frameworks are not proposed for clinical deployment in their present form, fairness evaluation is identified as a requirement for any future prospective validation.
Model output	15	D	Specify the output of the prediction model (e.g., probabilities, classification). Provide details and rationale for any classification and how the thresholds were identified	**Sections 2.4 and 2.5.** Outputs are: an AAR-3 burden of 0–3; a CARE-4 burden of 0–4; a continuous ACEF value; and assignment to one of three Gower–PAM phenotypes labelled neutrally as A, B and C in descending order of cluster size. The classification thresholds within AAR-3 and CARE-4 (age ≥75 years; WHO sex-specific anemia; eGFR <60 mL/min/1.73 m²; LVEF <40%) were chosen for clinical familiarity and interpretability and were not derived from the data. The manuscript states explicitly that the phenotype labels are not ordered risk categories

				and that no probability threshold was linked to a clinical action.

[1] D=items relevant only to the development of a prediction model; E=items relating solely to the evaluation of a prediction model; D;E=items applicable to both the development and evaluation of a prediction model
[2] Separately for all model building approaches.
[3] TRIPOD-Cluster is a checklist of reporting recommendations for studies developing or validating models that explicitly account for clustering or explore heterogeneity in model performance (eg, at different hospitals or centres). Debray et al, BMJ 2023; 380: e071018 [DOI: 10.1136/bmj-2022-071018]

Training versus evaluation	16	D;E	Identify any differences between the development and evaluation data in healthcare setting, eligibility criteria, outcome, and predictors	**Not applicable as external validation; Sections 2.1, 2.6, 3.6 and 4.7.** No separate independent evaluation dataset was used. Phenomapping, framework construction and framework evaluation were all performed within the same single-center retrospective cohort, and the manuscript states that this creates partial circularity. The closest internal analogue is the temporal-period analysis, in which the common complete-case cohort was split into 2017–2019 and 2020–2024 subcohorts; differences between the periods are reported, including a smaller number of current smokers with no events in the earlier period, which caused complete separation and required omission of that covariate from both period-specific models. These analyses are explicitly stated not to replace independent external validation.
Ethical approval	17	D;E	Name the institutional research board or ethics committee that approved the study and describe the participant-informed consent or the ethics committee waiver of informed consent	**Section 2.1; Institutional Review Board Statement.** The study was approved by the Ethics Committee of Izmir Katip Celebi University, Ataturk Training and Research Hospital (protocol code 0507; date of approval 11 June 2026) and was conducted in accordance with the Declaration of Helsinki. The requirement for informed consent was waived by the institutional ethics committee because of the retrospective observational design.
**OPEN SCIENCE**				
Funding	18a	D;E	Give the source of funding and the role of the funders for the present study	**Funding statement.** This research received no external funding. No funder was involved in the design, conduct, analysis, interpretation or reporting of the study.
Conflicts of interest	18b	D;E	Declare any conflicts of interest and financial disclosures for all authors	**Conflicts of Interest statement.** All authors declare no conflict of interest and report no financial disclosures relevant to this work.
Protocol	18c	D;E	Indicate where the study protocol can be accessed or state that a protocol was not prepared	**Not applicable.** No prospective study protocol was prepared or published, because this was a retrospective analysis of routinely collected clinical data. The analysis plan for the revised manuscript, including the outcome-independent cluster-selection rule and the hierarchical model structure, is described in full in Sections 2.4 and 2.6.
Registration	18d	D;E	Provide registration information for the study, including register name and registration number, or state that the study was not registered	**Not applicable.** This retrospective observational study was not prospectively registered in a trial or study registry.
Data sharing	18e	D;E	Provide details of the availability of the study data	**Data Availability Statement.** The de-identified data supporting the findings are not publicly available because of institutional and ethical restrictions governing patient-level clinical data. The data may be made available by the corresponding author upon reasonable request, subject to institutional approval and compliance with applicable data-protection regulations.
Code sharing	18f	D;E	Provide details of the availability of the analytical code[4]	**Data Availability Statement.**

				The analytical code may be made available by the corresponding author upon reasonable request under the same institutional and data-protection conditions. All analyses were performed in R version 4.6.0 within RStudio 2026.04.0, and the methods sections specify the procedures, resampling counts and random-initialization settings used.
**PATIENT & PUBLIC INVOLVEMENT**				
Patient & Public Involvement	19	D;E	Provide details of any patient and public involvement during the design, conduct, reporting, interpretation, or dissemination of the study or state no involvement.	**Section 2.8.** Patients or members of the public were not involved in the design, conduct, reporting, interpretation or dissemination plans of this retrospective study.
**RESULTS**				
Participants	20a	D;E	Describe the flow of participants through the study, including the number of participants with and without the outcome and, if applicable, a summary of the follow-up time. A diagram may be helpful.	**Section 3.1; Figure 1.** Of 3801 screened patients, 240 were excluded in five mutually exclusive categories, leaving a survival cohort of 3561 patients with 407 all-cause deaths over a median follow-up of 3.35 years. The Gower–PAM phenomapping cohort comprised 3491 patients with 394 deaths, the AAR-3 and CARE-4 descriptive cohorts 3557 and 3505 patients with 406 and 397 deaths, and the common complete-case cohort 3492 patients with 394 deaths. In the landmark analysis, 2977 patients remained alive and under observation beyond 365 days.
	20b	D;E	Report the characteristics overall and, where applicable, for each data source or setting, including the key dates, key predictors (including demographics), treatments received, sample size, number of outcome events, follow-up time, and amount of missing data. A table may be helpful. Report any differences across key demographic groups.	**Section 3.1 and 3.2; Table 1; Supplementary Tables S1, S2, S3, S5 and S13.** Baseline demographic, clinical, laboratory, inflammatory and echocardiographic characteristics are reported overall and across CARE-4 categories (Table 1), AAR-3 categories (Table S3) and Gower–PAM phenotypes (Table S5), together with follow-up duration, event counts and analytic cohort sizes. Variable-level missingness is reported in Table S1, and differences between included and excluded patients, including by sex and age, are quantified with standardized mean differences in Table S2. Period-specific characteristics and results are reported in Table S13. Treatment-related variables were not available and are therefore not reported.
	20c	E	For model evaluation, show a comparison with the development data of the distribution of important predictors (demographics, predictors, and outcome).	**Not applicable.** No separate external evaluation dataset was used, so a comparison of predictor and outcome distributions against an original development dataset is not applicable.
Model development	21	D;E	Specify the number of participants and outcome events in each analysis (e.g., for model development, hyperparameter tuning, model evaluation)	**Sections 3.1, 3.3, 3.5 and 3.6.** Phenomapping: 3491 patients, 394 deaths. Descriptive framework analyses: AAR-3 in 3557 patients with 406 deaths; CARE-4 in 3505 patients with 397 deaths. Primary head-to-head comparison: 3492 patients with 394 deaths, of which 161 deaths occurred within the first 365 days and 233 after the landmark among 2977 patients. Internal validation of the framework-specific models: 3544 patients with 403 deaths for AAR-3 and 3492 patients with 394 deaths for CARE-4 (Supplementary Table S15). Temporal periods: 1535 patients with

				208 deaths (2017–2019) and 1957 patients with 186 deaths (2020–2024). Decision-curve analysis: 3492 patients, 161 one-year deaths, with 351 patients censored before 365 days retained through the survival endpoint.
Model specification	22	D	Provide details of the full prediction model (e.g., formula, code, object, application programming interface) to allow predictions in new individuals and to enable third-party evaluation and implementation, including any restrictions to access or re-use (e.g., freely available, proprietary)[5]	**Sections 2.4, 2.5 and 2.6.** AAR-3 = [age ≥75 years] + [WHO-defined anemia: hemoglobin <13.0 g/dL in men, <12.0 g/dL in women] + [eGFR <60 mL/min/1.73 m²], range 0–3. CARE-4 = AAR-3 + [LVEF <40%], range 0–4. ACEF = age (years) / LVEF (%) + 1 point if serum creatinine >2.0 mg/dL. The reference models are fully specified: Model B contains age, sex, ACS subtype, diabetes mellitus, hypertension, atrial fibrillation, current smoking and prior coronary artery disease; Model C adds continuous hemoglobin, eGFR and LVEF. The scores can therefore be reproduced exactly by any third party from routinely available variables. By contrast, phenotype assignment depends on the Gower–PAM medoids derived in this cohort; because agreement with alternative clustering algorithms was low, the manuscript states that individual phenotype assignments should not be regarded as transportable to other datasets.
Model performance	23a	D;E	Report model performance estimates with confidence intervals, including for any key subgroups (e.g., sociodemographic). Consider plots to aid presentation.	**Section 3.5 and 3.6; Table 2; Supplementary Tables S9, S11, S13, S14 and S15; Supplementary Figures S2–S4.**<br><br>Against the age-inclusive reference model in the common complete-case cohort, Harrell's C-index was 0.770 (95% CI, 0.747–0.792) and increased to 0.793 (95% CI, 0.772–0.814) with AAR-3, 0.815 (95% CI, 0.795–0.834) with CARE-4, and 0.812 (95% CI, 0.792–0.833) with ACEF. The corresponding increases in C-index were 0.0231 (95% paired-bootstrap CI, 0.0119–0.0354), 0.0449 (0.0292–0.0603), and 0.0425 (0.0284–0.0567), respectively. The direct CARE-4 versus ACEF difference was 0.0024 (95% paired-bootstrap CI, −0.0109 to 0.0150), indicating numerically close estimates but not statistical equivalence. Adjusted hazard ratios with 95% confidence intervals, likelihood-ratio p-values, AIC and BIC are reported for every model.
	23b	D;E	If examined, report results of any heterogeneity in model performance across clusters. See TRIPOD Cluster for additional details[3].	**Sections 3.3 and 3.6; Supplementary Tables S4, S6 and S13.** No multicenter heterogeneity could be assessed in this single-center study. Within-method cluster stability was quantified using bootstrap Jaccard similarities (0.964, 1.000 and 0.893 with no dissolution across 100 resamples), whereas agreement with alternative clustering algorithms was low (adjusted Rand indices of 0.049 and 0.026), indicating substantial sensitivity of individual phenotype assignments to analytical choices. Score performance across the two calendar periods is reported in Table S13, with C-indices of 0.703–0.767 in the earlier period and 0.795–0.830 in the later period.

Model updating	24	E	Report the results from any model updating, including the updated model and subsequent performance	**Not applicable.** No model updating, recalibration or external revision was performed.
**DISCUSSION**				
Interpretation	25	D;E	Give an overall interpretation of the main results, including issues of fairness in the context of the objectives and previous studies	**Sections 4.1–4.6.** The results are interpreted as demonstrating statistical co-occurrence and association rather than mechanism or ordered biological risk classes. The Discussion separates the established biological literature from what the present data can support, emphasizes the attenuation of the phenotype effect after expanded clinical adjustment and the absence of incremental value beyond the continuous components, and notes that the lower observed mortality in phenotype B must not be read as a protective effect of smoking. Issues of fairness were not evaluated; this is stated rather than implied.
Limitations	26	D;E	Discuss any limitations of the study (such as a non-representative sample, sample size, overfitting, missing data) and their effects on any biases, statistical uncertainty, and generalizability	**Section 4.7.** Nine numbered limitations are discussed: retrospective single-center design and residual confounding; selection bias from the 240 exclusions, quantified by the included-versus-excluded comparison; censoring at last documented follow-up with possible informative censoring, and survivor selection in the landmark analysis; changing treatment practice and unavailable treatment-related variables; the all-cause endpoint without cause-specific mortality; single-timepoint early LVEF measurement variability and the absence of direct frailty, functional, nutritional, cognitive and sarcopenia measures; exploratory and method-dependent phenomapping, including the dominant influence of smoking on the selected partition; post hoc construction and partial circularity of AAR-3 and CARE-4 with the resulting possibility of optimism, and the impossibility of direct GRACE comparison; and the absence of any independent external validation cohort.
Usability of the model in the context of current care	27a	D	Describe how poor quality or unavailable input data (e.g., predictor values) should be assessed and handled when implementing the prediction model	**Sections 2.5 and 4.7.** AAR-3 and CARE-4 can be calculated only when all component variables are available; in this study they were therefore evaluated in complete-case cohorts rather than being computed from partially imputed components in the primary analysis. Implausible or poor-quality values should prompt source-data verification rather than automatic score calculation. Because LVEF was the component with the highest missingness (238 of 3801 records; 6.26%) and is also the most measurement-dependent, it is the input most likely to limit calculation in routine practice. No imputation-based fallback rule is proposed, and the frameworks are not recommended for clinical use in their present form.
	27b	D	Specify whether users will be required to interact in the handling of the input data or use of the model, and what level of expertise is required of users	**Sections 2.5 and 4.5.** The scores are simple additive counts that can be computed manually from age, hemoglobin, eGFR and LVEF, and require no specialized

				computational or machine-learning expertise. However, the manuscript states that they should not currently be used to direct clinical decisions, so no user workflow, software implementation or interface is proposed. Phenotype assignment, by contrast, would require the original Gower–PAM medoids and is not proposed for use outside this analysis.
	27c	D;E	Discuss any next steps for future research, with a specific view to applicability and generalizability of the model	**Sections 4.5 and 4.7; Conclusions.** Prospective multicenter studies should externally evaluate discrimination, calibration and clinical net benefit; compare the frameworks directly with established acute coronary syndrome risk models such as GRACE, using cohorts in which the required variables are collected; determine whether performance is stable across different populations, treatment practices and healthcare systems; and examine fairness across sociodemographic groups. Alternative mixed-data clustering approaches should be evaluated given the low cross-method agreement observed here. Only after such validation should interventional studies test whether score-informed follow-up pathways improve patient outcomes or resource use.

From: Collins GS, Moons KGM, Dhiman P, et al. *BMJ* 2024;385:e078378. doi:10.1136/bmj-2023-078378

[4] This relates to the analysis code, for example, any data cleaning, feature engineering, model building, evaluation.
[5] This relates to the code to implement the model to get estimates of risk for a new individual.
